# Application of the 3C Method to Study the Developmental Genes in *Drosophila* Larvae

**DOI:** 10.3389/fgene.2022.734208

**Published:** 2022-07-15

**Authors:** Oleg V. Bylino, Airat N. Ibragimov, Filomena Anna Digilio, Ennio Giordano, Yulii V. Shidlovskii

**Affiliations:** ^1^ Department of Gene Expression Regulation in Development, Institute of Gene Biology, Russian Academy of Sciences, Moscow, Russia; ^2^ Center for Precision Genome Editing and Genetic Technologies for Biomedicine, Institute of Gene Biology, Russian Academy of Sciences, Moscow, Russia; ^3^ Research Institute on Terrestrial Ecosystems (IRET), UOS Naples-CNR, Naples, Italy; ^4^ Department of Biology, Università di Napoli Federico II, Naples, Italy; ^5^ Department of Biology and General Genetics, I.M. Sechenov First Moscow State Medical University, Moscow, Russia

**Keywords:** chromatin conformation capture, distal interaction, larvae, chromatin conformation capture, chromatin, enhancer, promoter, *Drosophila*

## Abstract

A transition from one developmental stage to another is accompanied by activation of developmental programs and corresponding gene ensembles. Changes in the spatial conformation of the corresponding loci are associated with this activation and can be investigated with the help of the Chromosome Conformation Capture (3C) methodology. Application of 3C to specific developmental stages is a sophisticated task. Here, we describe the use of the 3C method to study the spatial organization of developmental loci in *Drosophila* larvae. We critically analyzed the existing protocols and offered our own solutions and the optimized protocol to overcome limitations. To demonstrate the efficiency of our procedure, we studied the spatial organization of the developmental locus *Dad* in 3rd instar *Drosophila* larvae. Differences in locus conformation were found between embryonic cells and living wild-type larvae. We also observed the establishment of novel regulatory interactions in the presence of an adjacent transgene upon activation of its expression in larvae. Our work fills the gap in the application of the 3C method to *Drosophila* larvae and provides a useful guide for establishing 3C on an animal model.

## 1 Introduction

The study of the basis of changes in the repertoire of active genes associated with the implementation of specific development programs is an important task of modern developmental biology. One of the basic changes of gene expression lies in the events that occur with a chromatin template. Current views on gene activity suggest that significant changes in chromatin conformation of corresponding loci accompany the developmental processes regardless of whether the development is discrete, like in *Drosophila*, or continuous, like in mammals, and can be investigated using Chromosome Conformation Capture (3C) methods (Du et al., 2017; Flyamer et al., 2017; Ke et al., 2017; Ogiyama et al., 2018; Sun et al., 2019; Collombet et al., 2020).

Cell cultures and embryos are the focus of many studies performed on *Drosophila* using C-methods. 3C protocols suitable for cell cultures are widespread, while experiments with embryos are less common (Sexton et al., 2012; Webber et al., 2013; Stadler et al., 2017; Hug and Vaquerizas, 2018; Erceg et al., 2019), and only a few experiments have been performed with individual tissues, for example, salivary glands of wandering 3^rd^ instar larvae (Eagen et al., 2015), wing imaginal discs (Li, 2016; Vizcaya-Molina et al., 2018), eye–antennal imaginal discs (Loubiere et al., 2020), imaginal discs (not specified) (Bernardo et al., 2014), fat body (Bernardo et al., 2014), and larval brain (Tolhuis et al., 2011). The same situation is observed in studies where whole larvae are used. There is a significant gap regarding the 3C procedure for the whole 3rd instar *Drosophila* larvae. This stage is of special interest since 3rd instar larvae have the largest and best developed imaginal discs and histoblast nests, which can be considered as non-specialized precursors of terminally differentiated adult cells that give origin to tissues of an adult fly during metamorphosis (Gilbert, 2010). Thus, understanding the differences in the state of genes between the last larval stage and adult flies, including the level of the spatial organization of chromatin, is very important for understanding how one stage of development turns into another, the terminal stage of an adult insect.

The study of the larval stages is a separate and rather difficult task. For example, *Drosophila* larvae of the 1st, 2nd, and middle 3rd instars live in fly food, and larvae of the late 3rd instar appear on the walls of fly tubes only transiently, before pupation. It is, therefore, difficult to collect a large amount of 3rd instar larvae. It is also difficult to collect large amounts of 1st and 2nd instar larvae, which are rather small in size.

A common feature of the abovementioned experimental works with *Drosophila* tissues is that the step of tissue fixation and homogenization yields a cellular material (cell suspension), which can further be processed using any type of the 3C protocol, including an *in situ*/in-nucleus ligation protocol (Comet et al., 2011; Rao et al., 2014; Nagano et al., 2015a, Nagano et al., 2015b, Nagano et al., 2017; Flyamer et al., 2017; Stadler et al., 2017; Bylino et al., 2021; Ulianov et al., 2021), a tethered ligation protocol (Kalhor et al., 2012; Eagen et al., 2015, Eagen et al., 2017; Gabdank et al., 2016), or a dilution ligation protocol (Dekker et al., 2002; Tolhuis et al., 2002; Lieberman-Aiden et al., 2009; Comet et al., 2011; Stadhouders et al., 2013; Ulianov et al., 2016; El-Sharnouby et al., 2017; Vermeulen et al., 2020). Therefore, it is of primary interest, first, to carefully consider the initial stages of the 3C procedure, including extraction of larvae from fly food, sorting them by age, and preparing a cell material from them.

Here, we described for the first time a detailed procedure of the 3C experiment with whole *Drosophila* larvae. Important initial steps of handling larvae, selecting the developmental stages, and preparing a cell material, were thoroughly considered. Next, the subsequent stages of preparation of experimental 3C and control BAC libraries, preparation of a calibration curve, and analysis of interactions are carefully described. To validate the results, electrophoretic pictures of the resulting 3C libraries are shown and raw qPCR data are provided to demonstrate that the libraries are well-amplified. Finally, to prove the efficiency of our procedure, we provided the experimental results obtained to determine the interactions between regulatory elements of the developmental locus *Dad* in S2 cells and wild-type (WT) and transgenic larvae. Our work fills the gap in the application of the 3C method to *Drosophila* larvae and provides a useful guide for establishing 3C on an animal model.

## 2 Results

### 2.1 Collection, Sorting, and Homogenization of *Drosophila* Larvae and Preparation of a Larval Cell Material

#### 2.1.1 Extraction of Total Larvae From Fly Food

Individual collection of late 3rd instar larvae from the walls of fly cultivation tubes is laborious and requires a large number of tubes with flies of the same developmental stage. It is more rational to extract all larvae from food with the help of 20% (585 mM) or 0.8 M sucrose. Upon adding 20% sucrose, the larvae float up with the liquid part of the food and can be collected individually from the surface after stirring the contents with a spatula with a groove to facilitate the process.

**FIGURE 1:**
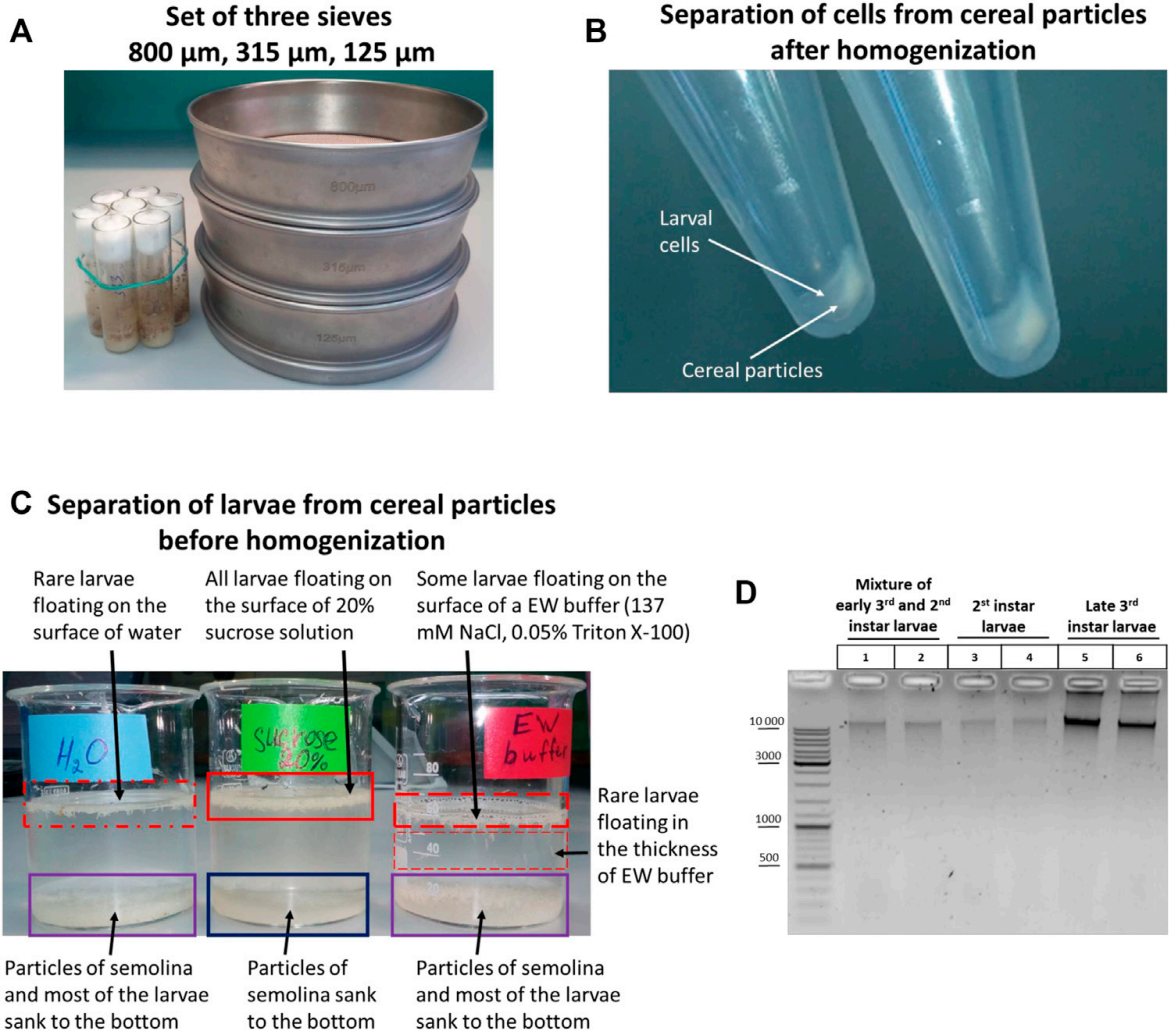
Initial stages of the 3C protocol: collection, sorting, and separation of larvae from fly food before and after homogenization. **(A)** TEST SIEVE Retsch GmbH 200 mm × 50 mm, 800 μm, 315 μm, 125 µm in comparison with standard vials for *Drosophila* cultivation. **(B)** Separation of larval cell material from cereal particles after homogenization. Total larvae were extracted from fly food as described in Section 2.1.1. Then the sucrose solution containing the liquid part of the food with the larvae was poured from fly vials into a set of 3 sieves depicted in Figure 1A, and the larvae were processed to obtain a cell material as described in *Processing of larvae extracted from fly food and preparation of cellular material* in *Materials and Methods*. After dissolving the cell material in ice-cold 1X PBS, the larval cell material was either i) applied onto a discontinuous sucrose gradient (0.8, 1.6, and 2.3 M sucrose steps) and centrifuged at 1,000 g at +4°С or ii) centrifuged through a 20% (0.584 M)/0.8 M sucrose cushion at 5,000 g. **(C)** Separation of larvae from fly food before homogenization. Larvae were extracted from fly food, as shown in **(B)**, and collected as described in Section 2.1.2. Then the larvae were transferred with a paintbrush from a 100-µm cell strainer into 3 different solutions: water, 20% sucrose, and EW buffer. The larvae floating on the surface of a liquid or deep in the solution are marked with a red rectangle; the density of the rectangle shading symbolizes the number of larvae. A blue rectangle shows the particles of cereal that sunk to the bottom. A purple rectangle shows a mixture of larvae and particles of cereal. In this experiment, the separation of larvae and cereal particles was performed by letting the solutions with larvae and cereal particles stand at RT for 30 min. **(D)** Chromatin integrity control obtained from WT Canton S larvae at different stages of development. Larvae were extracted from fly food, as shown in **(B)**. Then 3^rd^ instar larvae were individually collected and washed as described in *Individual collection of 3*rd *instar larvae for homogenization*. A mixture of early 3rd and 2nd instar larvae was collected, as shown in **(C)**. The 2nd instar larvae were collected individually with tweezers from the mixture of 3rd and 2nd instar larvae from a 315-µm sieve. After that, the larvae were processed, as shown in **(B)**. Larval cell material (25 mg) was taken and centrifuged, the supernatant was removed, and the 3C protocol was performed until obtaining the chromatin integrity control (see *Protocol of the 3C experiment with Drosophila larvae* in the Supplementary Material). 1/10 of the sample volume (2.5 mg) was taken as a control, and the control was processed according to the 3C protocol except that, to isolate DNA, 500 µl of an extraction buffer (EB) (see *Materials and Methods*) containing 30 mM EDTA and 0.2 mg/ml Proteinase K was added in each tube instead of 1X T4 DNA ligase buffer and the DNA was dissolved in 25 µl of 10 mM Tris-HCl, pH 8.0. Five µl of the DNA preparation was examined by electrophoresis. Two biological replicates were performed for each type of larvae.

If a large amount of larvae is required, a system of three sieves of different sizes can be used (Figure 1A). The sieves make it possible to separate larvae of different stages and, most importantly, to isolate the largest larvae of the 3rd instar, which precedes the adult stage. These larvae are the principal focus of this study. A 125-µm sieve retains 1st instar larvae, embryos, and water. An 800-µm sieve retains raisins, pieces of agarized food, dead adult flies, and the largest 3rd instar larvae. A 315-μm sieve retains early 3rd instar larvae and 2nd instar larvae.

#### 2.1.2 Mass collection of 3rd and 2nd Instar Larvae for Homogenization

If it is not necessary to separate the 3rd and 2nd instar larvae, larvae can be picked up from a 315 μm sieve using a spatula with a groove into a little glass with 20% sucrose. Then the larvae are transferred from the glass into a 100-µm nylon cell strainer (Corning Falcon, cat. no. 352360) and washed with the EW buffer. After washing, the entire mass of the 3rd and 2nd instar larvae are transferred using a paintbrush to a Dounce homogenizer of an appropriate volume and homogenized with pestle A to produce a cell material.

A disadvantage of this approach is that some amount of semolina or corn meal particles (depending on what cereal is used to prepare fly food) is collected together with the larvae when collection is carried out with a spatula with a groove from a 315-μm sieve. The particles sediment with cell material after homogenization to produce a uniform mixture of fixed cells and cereal particles. Experiments end in failure when performed with such a mixture. We concluded that it is necessary to separate fixed cell material from cereal particles after homogenization or, alternatively, to cleanly separate larvae from fly food before homogenization. Accordingly, we tried first to separate cell material from cereal particles after homogenization and found at the post-homogenization step that this method is inefficient, be it performed with a sucrose cushion (not shown) or a discontinuous sucrose gradient (Figure 1B); that is, the larval cell material and homogenized cereal particles co-sedimented simultaneously in both cases. Thus, it is necessary to separate larvae from fly food at an earlier stage before homogenization.

Next, we tried to separate larvae from fly food before homogenization and observed that when a mixture of larvae and cereal particles was left to stand for 20–30 min in a glass with 20% sucrose, but not the EW buffer or water, the cereal particles sank to the bottom, while live larvae float on the surface of 20% sucrose (Figure 1C). Then the larvae can be collected from the surface of 20% sucrose with a spatula with a groove in a 100-µm nylon cell strainer and washed with EW before homogenizing. In order to speed up the separation process, the larvae in 20% sucrose can be transferred from a glass into 50-ml tubes and centrifuged at 2,500 g for 1 min. In this case, the larvae slightly sink into the thickness of 20% sucrose and quickly float back to the surface after centrifugation, while the precipitated cereal particles do not. We concluded that the approach of separating larvae and cereal particles before the stage of larva homogenization proved to be efficient. Another option that would help to separate larvae from cereal particles is using fly food cooked without semolina or corn meal and, preferably, without raisins (the addition of cereals and raisins to fly food does not affect the total number of flies produced nor their rate of development, unpublished data).

#### 2.1.3 Individual collection of 3rd Instar Larvae for Homogenization

In the case where only 3rd instar larvae are required, but not a mixture of 2nd and 3rd instar larvae, 3rd instar larvae are individually collected with tweezers from an 800-μm sieve in a 100-ml beaker glass with 20% sucrose. The solution with larvae was poured into a 100-µm cell strainer, and the larvae were washed with EW buffer. Approximately 100 larvae or a little more (no more than 150) were necessary to collect. This number of larvae is easy to homogenize in a 7-ml Dounce homogenizer with pestle A, which disrupts tissues into individual cells. Filtration through a 40-µm cell strainer yields ∼50 mg of cell material, which can serve to make two replicates of 25 mg each. We observed that 200 or more larvae were hardly homogenized in a 7-ml Dounce homogenizer (there was risk of breaking the homogenizer or pestle), and a 15-ml Dounce homogenizer should be used with this number of larvae.

As shown in Figure 1D, 50 mg of cell material obtained from 2nd instar larvae contained a lower amount of DNA than 50 mg of cell material obtained from 3rd instar larvae; a mixture of 2nd and 3rd instar larvae contained an intermediate amount of DNA. Since larval growth and an increase in cell size are achieved primarily *via* endoreplication in *Drosophila* larvae, that is, cell growth and DNA replication occur in the absence of cell division, most larval tissues are composed mainly of polyploid cells (Edgar and Orr-Weaver, 2001; Edgar and Nijhout, 2004; Lee et al., 2009; Zielke et al., 2013; Ren et al., 2020). Therefore, a higher degree of cell polytenization in 3rd instar larvae is apparently responsible for the extraction of a greater DNA amount from the same amount of cell material. We used only 3rd instar larvae in subsequent experiments.

Alternatively, embryos can be grown in large jars of a large volume, for example, 200 ml, and 3rd instar larvae that have crawled onto the walls can be collected with a paintbrush or tweezers or rinsed off the walls with water. Extraction with sucrose will be especially convenient for those who want to get larvae of earlier instars and separate them into size fractions by age.

### 2.2 Processing of Larvae Extracted From Fly Food and Preparation of Cell Material

This chapter is given in the beginning of the Materials and Methods section (see Supplementary Material).

Two important methodological observations were made while preparing 3С libraries from wild-type (Canton S) and mutant (transgenic) larvae:

(i) The DNA yield from 10 mg of WT larval cell material pellet was approximately 10–15 times lower than from the same quantity of S2 cells. Although most *Drosophila* larval tissues are composed mainly of polyploid cells (Edgar and Orr-Weaver, 2001; Edgar and Nijhout, 2004; Lee et al., 2009; Zielke et al., 2013; Ren et al., 2020), we suppose that the pellet that forms after filtration through a 40-µm cell strainer and that is analyzed may contain a sufficiently large number of cells of the imaginal disc and histoblast nest (mitotic cells), diploid in nature, as well as extracellular material, such as remnants of the milled cuticle. This may be one of the reasons that cell material obtained from larvae contains less DNA than the same quantity of S2 cells. Another reason is that S2 cells have an altered karyotype and their ploidy varies from 1 to more than 8, with an average karyotype of 2X; 4A corresponding to tetraploids (Zhang et al., 2010; Lee et al., 2015). Therefore, it is very likely that a tetraploid cell culture of the same quantity contains more DNA than milled larval tissues.
(ii) The amount of DNA isolated from the same quantity of cell material obtained from mutant lines carrying transgenic insertions significantly varied from line to line and was generally reduced (at least by a factor of 2–3) as compared with the WT Canton S strain. This may be due to the poor genetic background in the mutant lines or impaired expression of the *Dad* gene. *Dad* expression is required for inhibiting the BMP/Dpp/TGF-β signal transduction pathway (Tsuneizumi et al., 1997; Marquez et al., 2001; Sharifkhodaei and Auld, 2021). Direct suppression of endomitosis and endoreplication has been found to occur upon stimulation of the BMP/Dpp/TGF-β pathway (Kuter et al., 1992), although an opposite situation is observed, for example, in nematodes, where the BMP/Dpp/TGF-β pathway stimulates endoreplication (Nystrom et al., 2002; Lozano et al., 2006). Nevertheless, these data taken together implicate the BMP/Dpp/TGF-β pathway in controlling endoreplication

### 2.3 Processing of the Cell Material Prepared From *Drosophila* Larvae

Taking into account the abovementioned observations, the amount of starting cell material to prepare a 3C library should be at least 25 mg in the case of WT larvae and at least 50 mg in the case of mutant larvae. It is necessary to check the DNA yield for each particular mutant line since the yield may be significantly lower than that for WT larvae. A comprehensive protocol of the 3C experiment with *Drosophila* larvae, from the processing of cell material to obtain a purified 3C library to statistical analysis of the 3C experiment results, is presented in the Supplementary Material. The part devoted to processing cell material covers steps 1 through 25 of the protocol and comprises the following sections:

I. Cell lysis
II. Nucleoplasm release and chromatin treatment with heat
III. Digestion of DNA in nuclei
IV. Ligation of DNA in nuclei
V. Reversion of cross-links and isolation of a 3C library
VI. Treatment of the 3C library with RNase
VII. Purification of the 3C library on magnetic beads and DNA analysis

The stages of reversion of cross-links and isolation of a 3C library, treatment of 3C library DNA with RNase, and purification of the 3C library on magnetic beads and DNA analysis can be used as independent protocols and have been thoroughly discussed by Bylino et al. (2021).

### 2.4 Preparation of a Control Library (Random Ligation Library) for Constructing a Calibration Curve

The next step in the 3C procedure is to obtain a control library consisting of Sau3A (BspI)-digested and then randomly ligated DNA fragments of BAC(s) overlapping the locus (loci) of interest. If the locus contains transgenic sequences, the BAC can be mixed in an equimolar ratio with a plasmid carrying such sequences. A control library is necessary for constructing a calibration curve, which is used to calculate the relative ligation frequencies (RLFs) of the fragments of interest in 3C samples. We studied the conditions of BAC cultivation, isolation, purification, restriction digestion, and random ligation of the resulting restriction fragments. These stages are described in detail in section 2 of the Supplementary Material (subsections 2.1, 2.2, 2.3, and 2.4). This section can be used as an independent protocol to grow and purify BAC and to prepare a random ligation library. The part devoted to the preparation of a random ligation BAC library covers steps 26 through 34 of the protocol in the Supplementary Material and comprises the following sections:

VIII. Preparation of a random ligation BAC library for constructing a calibration curve

The main conclusions from our experiments and optimization of the above stages of the protocol are as follows:

(i) Induction of BAC replication (CHORI321 library pCC1BAC series) occurs in the presence of L-arabinose in the culture medium but not in the presence of inactivated tetracycline hydrochloride or chlortetracycline.
(ii) In the absence of a replication inducer, the BAC behaves as an ordinary multicopy plasmid despite the presence of a single-copy origin of the F-factor, increasing in copy number in the presence of chloramphenicol (Cm). The BAC copy number rapidly decreases when Cm is absent or consumed in the medium, and the BAC yield becomes very low.
(iii) The BAC can be efficiently isolated with a usual plasmid isolation kit.
(iv) Purification of the BAC on AMPure XP beads appears to be more efficient than standard EtOH precipitation (without the addition of glycogen).
(v) Equimolar mixing of the BAC and the plasmid leads to an equally represented ligation of the transgene and the BAC fragments in the resulting control library.

### 2.5 Construction of a Calibration Curve and Analysis of the 3C Library

The next step of the 3C procedure is analyzing the ligation frequencies in the experimental 3C library (described in detail for S2 cells by Bylino et al. (2021) and for larvae in the Supplementary Material). Measurement of the number of ligation events between spatially adjacent DNA regions in genome-wide 3C (Hi-C) (Lieberman-Aiden et al., 2009; Rao et al., 2014; Belaghzal et al., 2017; Akgol Oksuz et al., 2021; Lafontaine et al., 2021) or in 3C covering a few (Capture-C) (Hughes et al., 2014; Davies et al., 2016; Golov et al., 2020a; Hua et al., 2021) or more (promoter capture Hi-C) (Mifsud et al., 2015; Schoenfelder et al., 2018a) regions of interest is based on the results of sequencing on the Illumina platform (Davies et al., 2017; Grob and Cavalli, 2018). Its prohibitive cost means that such datasets are reasonable to obtain when a large number of interactions is to be analyzed or when interaction partners are unknown. However, when a limited number of interactions between several DNA regions is of interest, the ligation frequency can be determined using real-time qPCR. To achieve a greater sensitivity of the method, TaqMan probes are used instead of SYBR Green–based detection. A qPCR analysis of the ligation frequencies utilizes a calibration curve, which is obtained from 10X dilutions of a random ligation mixture of control DNA digested with the same restriction endonuclease (RE) or a RE that has the same cleavage site. The calibration curve can be quantitative, that is, based on purified PCR products, which are used in known concentrations, overlap the restriction sites of interest, and are mixed in equimolar amounts before random ligation, or semi-quantitative, that is, based on random ligation of a digested BAC that covers the region of interest (relative ligation frequencies) (Gavrilov et al., 2013). When a transgenic construct is present in a region of interest, a plasmid(s) containing the cloned region(s) or PCR amplicons of transgenic regions can be combined with the BAC in equimolar amounts before preparing a random ligation library mix (Shidlovskii et al., 2021).

Before analyzing the ligation frequencies between restriction fragments of interest in experimental 3C samples, several important preliminary experiments should be performed: 1) to optimize the conditions of qPCR with TaqMan probes, which will be used to determine the ligation frequencies in experimental 3C libraries (optional); 2) to study the ligation frequencies in random ligation libraries (optional); 3) to determine the linear range of amplification by testing several dilutions of a random ligation library; and 4) to establish the amount of 3C DNA libraries required to fit the calibration curve (in our case, this was done with a model of S2 cells and then with a model of WT larvae). These stages are described in detail in section 3 of the Supplementary Material (subsections 3.1, 3.2, 3.3, and 3.4). The part devoted to the preparation and preliminary testing of the calibration curve covers steps 26 through 35 of the protocol and comprises the following sections:

IX. Preparation of the calibration curve based on a random ligation library
X. Preliminary testing of dilutions of 3C samples against the calibration curve

The main conclusions that can be drawn from our experiments and optimization of the above stages of the protocol are as follows:

(i) The detection threshold of the PCR product in the reaction increases in direct proportion to the amount of the TaqMan probe added to the reaction. The greater the probe amount, the earlier occurs the threshold of 3C product detection (an increase in probe concentration from 0.05 to 1 pM/µl leads to the appearance of a signal 4.5 ± 0.5 Ct earlier). Therefore, if the DNA content in the 3C library is low and a limited amount of the starting material is available for preparing the 3C library, then increasing the concentration of the TaqMan probe in the reaction up to 1 pM/μl makes it possible to detect the product without generating additional amounts of the 3C library.
(ii) When a plasmid containing the transgene and a BAC containing a genomic locus of interest are combined in equimolar proportion, a mixture of restriction fragments is obtained, in which the ligation products of different regions are equally represented.
(iii) A calibration curve prepared in a range from 1 ng/μl to 100 fg/μl from 10X dilutions of a random ligation library as described in section 4 and in the protocol for larvae in the Supplementary Material (step 35) can be routinely used to determine the cross-linking frequency in an experimental 3C library. Each dilution is used in 4 technical replicates in an amount of 5 μl per PCR mixture (then the template DNA concentrations will be 5 ng–500 pg–50 pg–5 pg–500 fg per PCR mixture, respectively). A linear amplification region corresponds to a 10X dilution range from 10 ng/μl to 100 fg/μl. The step between two calibration dilutions is 3.5–4.0 Ct. It is convenient to select 4 dilutions to be used in the experiment in parallel with the 3C samples.
(iv) 3C libraries prepared from 10 mg of S2 cells according to the protocol described by Bylino et al. (2021) are possible to dilute by a factor of 10–15 (to a concentration of 1.24–6.23 ng/μl) without impairing the reliability of contact determination. With such a concentration of 3C libraries in the PCR mixture, the calibration curve is built from dilutions of 100 pg–10 pg–1 pg–100 fg per μl (the product appears between the 10 pg/μl and 1 pg/μl dilutions of the calibration curve). The quantity of S2 cells required for the preparation of a 3C library is 10 mg and may be diminished even to 5 mg if necessary. A twofold increase in the DNA concentration of the 3C library in PCR gives an increase of 1.75–2.0 Ct for S2 cells and for WT larvae.
(v) At least 5–10 ng of the 3C library per PCR mixture prepared from WT larvae is sufficient for detecting the ligation products between DNA regions of interest at 100 pg–10 pg–1 pg–100 fg dilutions of the calibration curve per µl (the product appears between the 1 pg/μl and 100 fg/μl dilutions). For additional depth of the calibration curve in the case of a limited amount of 3C library DNA from larvae, a 10 fg/μl dilution can be introduced into the range of the calibration curve. The dilution is set in 8, rather than 4, technical replicates. However, the dilution does not always fall within the linear range of amplification.
(vi) At least 12–46 ng of 3C library DNA prepared from mutant larvae is necessary to take in a PCR mixture for reliable detection of ligation products (the product appears between the 1 pg/μl and100 fg/μl or between the 10 and 1 pg/μl calibration curve dilutions, depending on the mutant line and the amount of DNA taken into the PCR mixture).
X. The stages of preparation of the qPCR master mix and the general arrangement of a qPCR experiment with 3C samples, normalization of ligation frequencies, calculation, and statistical analysis of the 3C experiment results are described in detail in section 4 of the Supplementary Material (subsections 4.1, 4.2, and 4.3). The part devoted to the preparation and preliminary testing of the calibration curve covers steps 36 through 39 of the protocol in the Supplementary Material and comprises the following sections of the protocol: Preparation of the PCR master mix and the general arrangement of a qPCR experiment with 3C samples.
XI. Normalization of ligation frequencies
XII. Calculation and statistical analysis of the 3C experimental results

### 2.6 Critical Analysis of the Existing 3C Protocols for Whole *Drosophila* Larvae. Optimal Parameters, Peculiarities, and Advice on Preparing and Processing Cell Material Obtained From Larvae

Although a sort of universal 3C protocol has been previously published to describe the processing of collected adult flies, pupae, and embryos and to prepare cell material from them (Comet et al., 2011), no detailed procedure has been reported yet to allow 3C investigations in *Drosophila* whole larvae. Prior to our study, only one study was published to describe 3C with whole *Drosophila* larvae (Bieli et al., 2015). A critical analysis of the very brief procedure described by Bieli et al. (2015) revealed several issues that would require significant changes. Based on our own experience with larva processing and treatment of the cell material obtained from them and from S2 cells (Bylino et al., 2021; Shidlovskii et al., 2021), we suggest the following important improvements to the previously published procedure:

(i) Fix larvae simultaneously with homogenization in a Dounce homogenizer with FA used at a concentration not exceeding 0.5% for 10 min (we found that fixation with 1.8% FA for 20 min is excessive). We observed that tissues of larvae are over-fixed even when fixation is performed with 0.5% FA for 25 min and that DNA of over-fixed cells is poorly cut by a restriction endonuclease (RE) and is not extracted with Ph/Chl, remaining in the non-lysed cells (Bylino et al., 2021).
(ii) Quench FA with glycine used at an equimolar concentration or in a slight excess to FA (Comet et al., 2011; Sexton et al., 2012), keeping in mind two reactive groups of FA vs. one group of glycine. For example, we used glycine at 666 (equimolar) and 800 (slight excess) mM for 1% FA (666 mM molarity by the number of reactive groups). Do not quench with a strong molar deficiency of glycine to FA (with 0.125 M glycine) since quenching is not likely to be complete in these conditions (Splinter et al., 2012) or, if doing so, immediately proceed to the next step after quenching without storing the material (even after flash freezing since cell fixation may still proceed during thawing).
(iii) Do not store fixed larval cell material inactivated with glycine and washed with ice-cold 1X PBS on ice for several days, for example, until controls #1 (chromatin integrity) and #2 (chromatin restriction digestion) are ready to understand if chromatin is not degraded and well digested. Fixation followed by keeping the cell material on ice does not allow maintaining the DNA integrity. Degraded DNA was isolated from the cell material and formed a smear, which disappeared after treatment with bovine RNase A, which digests DNA, especially in a degraded form, according to our results (discussed by Bylino et al. (2021)). Instead, proceed to the cell lysis stage immediately without storing the cell material.
(iv) Do not use a too high RCF (10,000 g and higher), but centrifuge larvae and cells at an RCF not exceeding 5,000–7,500 g. It has been shown that centrifugation at more than 8,000 g leads to broken, sheared nuclei (Louwers et al., 2009).
(v) Do not use mechanical force when treating the nuclei, and handle the nuclei gently. For example, do not pass the nuclei through a syringe needle because this can affect the integrity of the nuclei and DNA. Instead, use a 40-µm cell strainer to filter the homogenate to obtain a cellular material (Bylino et al., 2021).
(vi)Use chromatin treatment modes at 65°C (65°C with SDS for 5–10 min and 37°C with Triton X-100 for 15 min) as it is preferable for larval cells. We observed that the regimens of chromatin treatment with SDS at 37°C (37°C with SDS for 10 min and Triton X-100 for 15 min or 37°C with SDS for 1 h and Triton X-100 for 1 h), which provide for more efficient ligation in the case of S2 cells (Bylino et al., 2021), did not give the same improvement in the case of larval cell material.
(vii) After chromatin heat treatment at 37/65°C in the presence of SDS/Triton X-100, wash the nuclei with 1X restriction buffer (RB) before restriction digestion (Flyamer et al., 2017) since, even sequestrated with Triton X-100, SDS is able to hamper the RE function at high concentrations (Louwers et al., 2009) and only a few REs can tolerate the conditions of 0.3% SDS sequestrated with 1.8–2% Triton X-100 (Splinter et al., 2012; van de Werken et al., 2012).
(viii) Omit the step of inactivation of RE at 65°C for 10 min in the presence of high SDS concentrations (∼1.2–1.3%) as it negatively affects the structure of nuclei (Nagano et al., 2015b). Instead, wash off the nuclei from RE with 1X T4 DNA ligase buffer before DNA ligation (Nagano et al., 2013; Nagano et al., 2015a; Nagano et al., 2015b, Nagano et al., 2017).
(ix) Do not ligate chromatin in the presence of 0.1% SDS even sequestered with 1% Triton X-100. We observed that 0.1% SDS even sequestered with 1% Triton X-100 gives strong inhibition during in-nucleus DNA ligation (Bylino et al., 2021), although ligation in a solution of plasmid DNA cleaved by 6 bp cutter in the presence of 0.1% SDS sequestered with 1% Triton X-100 appear to be efficient (Gavrilov et al., 2013). It has previously been proposed to wash the nuclei before DNA ligation (Flyamer et al., 2017; Golov et al., 2020b). We examined this important issue and found that at least a triple washing of larvae nuclei suspension with 1X T4 ligase buffer at this stage efficiently prevents inhibition and does not lead to DNA degradation (Bylino et al., 2021).
(x) After Ph/Chl extraction, process 3C library preparations with RNase I and then purify them on AMPure XP paramagnetic beads (SPRI technology) in order to remove RNase and to additionally purify the libraries before PCR determination of ligation frequencies. We observed that RNase I is efficient in removing RNA impurities from DNA preparations and the «safest» RNase for DNA treatment (Bylino et al., 2021). Use a T4 DNA ligase concentration of at least 0.025 WeissU/µl. We determined that the T4 DNA ligase concentration of 0.0025 WeissU/μl is inefficient during in-nucleus ligation and that an efficient concentration range is from 0.025 to 0.25 WeissU/μl (Bylino et al., 2021).

The abovementioned steps were tested in our previous studies (Bylino et al., 2021; Shidlovskii et al., 2021) and in this work and were found to ensure the preservation of DNA integrity at all stages. Our improvements allow the stable detection of distant DNA site interactions in 3C libraries prepared from at least 25 (WT larvae) or 50 (transgenic mutant larvae) mg of starter material. For a more detailed discussion of the steps described earlier, see the main part and the supplementary in the study by Bylino et al. (2021).

### 2.7 Analysis of Distal Interactions in the Developmental Locus *Dad* in *Drosophila* Larvae

#### 2.7.1 Detection of the *Dad* Enhancer Interactions in *Drosophila* Wild-type Larvae. Comparison of the 3C Profiles in Wild-type Larvae and S2 Cells

**FIGURE 2:**
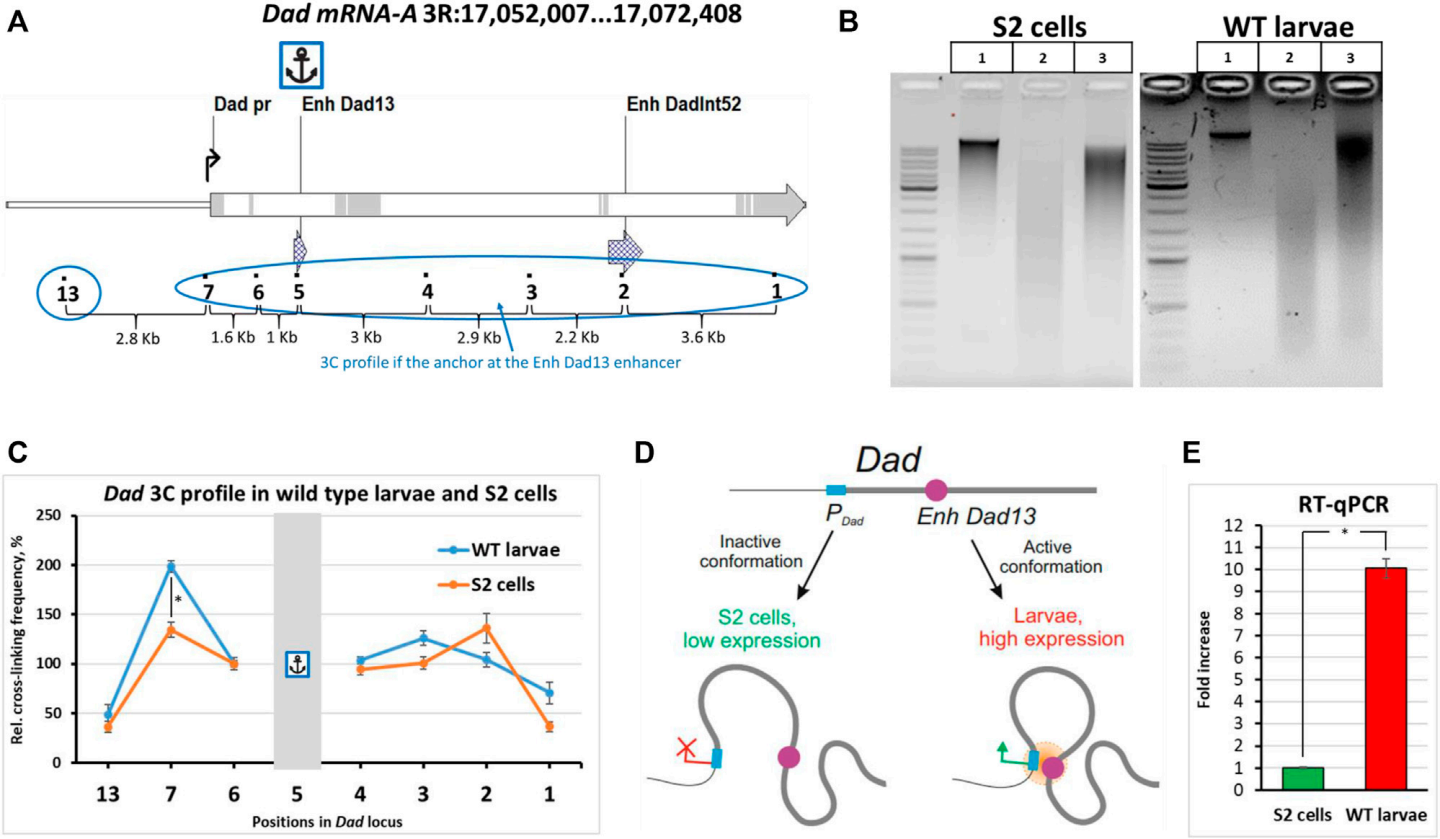
Comparison of the 3C profiles of live wild-type *Drosophila* larvae and S2 cells. **(A)** The model locus *Dad* with an anchor on the enhancer *Dad13* in WT *Drosophila* larvae and S2 cells is shown. Designations are as shown in Supplementary Figures S3A. Exons are highlighted grey; introns are shown white. **(B)** A representative example of the 3C libraries prepared from WT larvae and S2 cells. Larvae were extracted from fly food, as shown in Figure 1B. One hundred WT larvae were individually collected and washed, as shown in Figure 1D, and then processed to obtain a cell material, as shown in Figure 1B. Larval cell material (25 mg) was taken for 3C library preparation. The cell material was centrifuged, the supernatant was removed, and a 3C library and controls #1 and 2 were prepared as described in *Protocol of the 3C experiment with Drosophila larvae* in the Supplementary Material. The 3C libraries and controls of S2 cells were prepared as shown in Supplementary Figure S6 except that control #1 and the ligation mixture were purified using a 1.5X volume of AMPure XP beads and control #2 was purified using a 2X volume of AMPure XP beads. For electrophoresis, in the case of S2 cells, 200 ng of DNA was used for controls #1,2 and the ligation mixture. In the case of larvae, the DNA amount resolved in the gel was 25 ng for control #1, 50 ng for control #2, and 75 ng for the ligation mixture. Lane 1, control #1 (chromatin integrity control); lane 2, control #2 (chromatin digestion control); lane 3, ligation mixture (3C sample). **(C)** Comparison of the 3C profile between live WT Canton S larvae and S2 embryonic cells. Six independent biological replicates of the 3C library were analyzed for larvae and for S2 cells. The concentrations of all 3C libraries were made equal according to Qubit readings before measurements. The frequency of ligation of the anchor fragment with the adjacent fragment (point #6) was arbitrarily taken as 100%, and the values of all other experimental points were calculated proportionally. The relative ligation frequencies (RLFs) of experimental samples were normalized to RLFs within the constitutively expressed *RpII* locus. Error bars indicate SEMs from six independent biological replicates of the 3C library. Each experimental point for each 3C library was studied in 4 technical PCR replicates, and the data were averaged. One-tailed Student’s t-test was used for comparison between groups to calculate the reliability of the revealed differences. An asterisk indicates the significance level: *p < 0.05, n = 12. **(D)** A model illustrating spatial differences in the location of regulatory elements of the developmental *Dad* gene in cultured cells and live *Drosophila* larvae. **(E)** Evaluation of the expression level of the *Dad* gene in S2 cells and wild-type larvae. Total RNA was prepared, and the mRNA level was measured as described in the Isolation of total RNA and RT-qPCR section of Materials and Methods. For reverse transcription, the same amount of RNA was taken from S2 cells and wild-type Canton S larvae. The *Dad* gene expression level was normalized to the *β-tubulin 56D* gene expression level. The two genes showed similar amplification efficiencies. The *Dad* mRNA content was calculated using the ΔΔCt method. In addition to the ΔΔCt, a calibration curve prepared from S2 cell DNA as described in Materials and Methods was used in the experiment. The results obtained using the calibration curve and ΔΔCt were similar. The *Dad* expression level in S2 cells was arbitrarily taken as 1. Three independent total RNAs were prepared from S2 cells and from larvae, and each RT was studied in 4 PCR technical replicates. Error bars indicate SDs of 4 PCR technical measurements from three independent biological replicates of total RNA. One-tailed Student’s *t*-test was used to evaluate the reliability of differences in between-group comparisons. An asterisk indicates the significance level: *p < 0.001, *n* = 6.

To validate our optimizations of the 3C procedure for *Drosophila* larvae, we chose the developmental locus *Dad* (daughters against *decapentaplegic*) as a model (Figure 2A). The *Dad* gene is a regulatory response gene in the BMP/Dpp/TGF-β pathway and encodes the receptor-binding protein important for negative feedback in the transmission of the signal from receptors activated by the Dpp ligand (Tsuneizumi et al., 1997; Marquez et al., 2001; Sharifkhodaei and Auld, 2021). The BMP/Dpp/TGF-β signal transduction pathway is important for the growth, proliferation, and differentiation of cells of imaginal discs (Peterson and O’Connor, 2014; Upadhyay et al., 2017). The *Dad* gene has two enhancers, the proximal *Dad13* enhancer (the main enhancer) and the distal *DadInt52* enhancer (shadow enhancer) (Weiss et al., 2010; Neal et al., 2019). Both enhancers are located in the introns of the gene (Figure 2A). We studied the interactions of the enhancer *Dad13* with the regions inside the *Dad* locus in WT larvae and compared the resulting 3C profile with the profile obtained for S2 embryonic cells. Individually collected 3^rd^ instar Canton S larvae were used. 3C libraries were prepared and analyzed as described earlier. Electrophoretic analysis of the 3C libraries is given in Figure 2B.

When we addressed the 3C profile, we found that the profile of *Dad13* enhancer interactions in larvae differed from that obtained for cultured cells. Although the upstream and downstream regions interacted with the enhancer weakly in both larvae and S2 cells, the promoter region was significantly closer to the enhancer in larvae than in S2 cells (Figure 2C, Supplementary Figures S10 and S11). The situation did not differ when a smaller amount of larval cell material was used to prepare the 3C library (Supplementary Figure S12C). This means that our data fall within the linear sensitivity range of the 3C method. Thus, our data suggest that there are differences in the proximity of the enhancer to the promoter between WT larval cells and cultured embryonic S2 cells (Figure 2D). The differences may be associated with the *Dad* expression level. Indeed, *Dad* is expressed at a low level in cultured cell lines and is active in 3rd instar larvae, according to databases (Chintapalli et al., 2007; modENCODE Consortium et al., 2010; Larkin et al., 2021). To check this experimentally, we performed RT-qPCR using equal amounts of S2 and WT 3rd instar larval cell material and found that the *Dad* expression level in cultured S2 cells was approximately 10 folds lower than in larval cells. Thus, the differences in the enhancer–promoter proximity within the *Dad* locus correlate with the gene activity. The inactive state of the *Dad* locus in S2 cells can be accounted for by its localization in the B compartment in S2 cells and embryos (Sexton et al., 2012; Li et al., 2015; Cubeñas-Potts et al., 2017; Eagen et al., 2017).

#### 2.7.2 3C Profile of the *Dad* Locus in the Presence of an Adjacent Transgene

**FIGURE 3:**
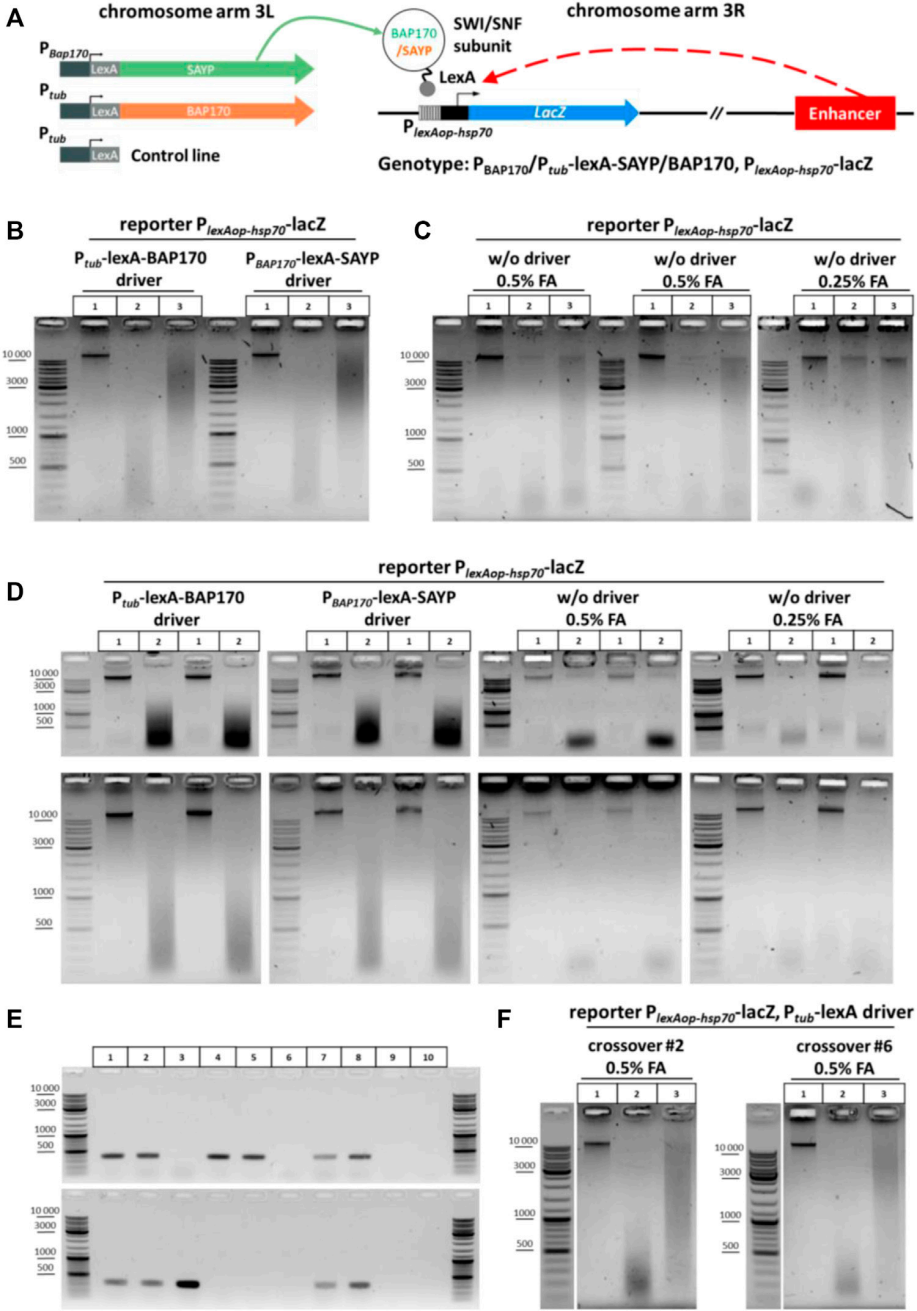
General scheme of the experiment with transgenic larvae, screening of crossovers, and 3C libraries obtained from the transgenic larvae. **(A)** Genetic structure of transgenic flies and a general arrangement of the experiment with transgenic larvae used to determine the role of SAYP or the BAP170 Brahma/SWI/SNF subunits in enhancer–promoter interactions. The SWI/SNF subunits BAP170 and SAYP tethered to the reporter promoter induce activation of the reporter in an adjacent enhancer-dependent manner. **(B)** 3C libraries prepared from larvae carrying the reporter P_*lexAop-hsp70*_-lacZ transgene in the presence of either P_*tub*_-lexA-BAP170 or P_*BAP170*_-lexA-SAYP driver. The combinations of transgenes are indicated above the electrophoretic pictures. The experiment was performed as shown in Figure 2B for larvae except that 150 larvae were collected and 50 mg of larval cell material was taken for 3C library preparation. Lane 1, chromatin integrity control; lane 2, chromatin digestion control; lane 3, ligation mixture (3C sample). At least seven independent 3C libraries were prepared for each genotype. A representative example is shown for two 3C libraries. **(C)** 3C libraries prepared from larvae carrying the P_*lexAop-hsp70*_-lacZ reporter only. 3C libraries and controls were prepared as shown in **(A)**. Lane 1, chromatin integrity control; lane 2, chromatin digestion control; lane 3, ligation mixture (3C sample). At least seven independent 3C libraries were prepared. Three biological replicates obtained after fixation with different FA concentrations are presented. **(D)** Electrophoretic analysis of chromatin integrity controls and restriction digestion controls prepared from larvae carrying the P_*lexAop-hsp70*_-lacZ reporter with or without the P_*tub*_-lexA-BAP170 or P_*BAP170*_-lexA-SAYP driver. The controls were prepared as shown in **(B)** except that they were not treated with RNase. The top row of pictures shows the same samples but resolved electrophoretically for a shorter time. Lane 1, chromatin integrity control; lane2, chromatin digestion control. Two independent replicates are shown for each genotype. **(E)** PCR analysis of the crossover lines carrying the P_*tub*_-lexA control driver and P_*lexAop-hsp70*_-lacZ reporter transgene in the same chromosome. A representative analysis is given in comparison with other lines. Genomic DNA was extracted from flies as described in the section *Genomic DNA isolation from fly lines for screening of crossover flies* of *Materials and Methods*. Lane 1, P_*tub-lexA*_-BAP170 driver + P_*lexAop-hsp70*_-lacZ reporter; lane 2, P_*BAP170-lexA*_-SAYP driver + P_*lexAop-hsp70*_-lacZ reporter; lane 3, reporter transgene P_*lexAop-hsp70*_-lacZ only; lane 4, control driver transgene P_*tub*_-lexA (insertion in chromosome 3 #1); lane 5, control driver transgene P_*tub*_-lexA (insertion in chromosome 3 #2); lane 6, WT Oregon R; lane 7, crossover #2 (insertion from lane 4 was combined with the reporter transgene from lane 3); lane 8, crossover #6 (insertion from lane 5 was combined with the reporter transgene from lane 3); lane 9, control PCR with water instead of DNA (negative control #1); lane 10, control reaction with 50 mM Tris-HCl, pH 8.0 instead of DNA (negative control #2). **(F)** Representative examples of 3C libraries prepared from crossover lines. Larvae, 3C libraries, and controls were processed as shown in **(B)**. Designations of lanes are as shown in Figure 2B. One replicate is shown for each crossover line.

***Description of the experimental reporter system***. We have previously described the enhancer-trap system in flies that contains a reporter transgene P_*lexAop-hsp70*_-lacZ activated by a nearby enhancer in a Brahma complex (SWI/SNF)-dependent manner (Shidlovskii et al., 2021). The flies carry a P-element insertion of the reporter *lacZ* gene into the 5′ region of the *Dad* gene in the 3R arm of the third chromosome (Figure 3A). The reporter was placed under the control of the minimal *hsp70* promoter fused with the operator sites for the DNA-binding domain (DBD) of the LexA protein (P_*lexAop-hsp70*_-lacZ). The flies also have a second transgene, which is integrated in the attP2 site in the 3L arm of the same chromosome (Markstein et al., 2008; Pfeiffer et al., 2008, Pfeiffer et al., 2010), thus expressing a fusion protein that consists of one of the signature subunits of the Brahmа complex (SAYP or BAP170) and DBD of the LexA protein (Figure 3A). These subunits are conservative (SAYP is a homologue of PHF10 in humans; BAP170 is a homologue of ARID2 in humans and RSC9 in yeast) and specify the PBAP/PBAF subtype of the Brahma (SWI/SNF) chromatin remodeling complex. The driver transgene in 3L was placed under the control of P_*tub*_ (BAP170 fusion) or P_*BAP170*_ (SAYP fusion) promoter. As was previously shown, targeted recruitment of the SAYP-lexA or BAP170-lexA fusion to the LexA operator sites in the *lacZ* promoter was accompanied by reporter gene activation in an enhancer-dependent manner with a pattern similar to that of the endogenous *Dad* gene (Shidlovskii et al., 2021). In this regard, it is of interest to elucidate whether such activation is accompanied by spatial convergence of the captured enhancer and the reporter gene promoter. For this purpose, a 3C library was prepared from 3^rd^ instar larvae of fly lines carrying the P_*lexAop-hsp70*_-lacZ reporter transgene with any of the two other transgenes that coded for a fusion of either SAYP or BAP170 with the LexA DBD (P_*tub*_-lexA-BAP170 or P_*BAP170*_-lexA-SAYP driver transgenes). Larvae that carried only the P_*lexAop-hsp70*_-lacZ reporter transgene were used as a control.

***Genetic background affects digestion by restriction enzyme in 3C procedure***. The chromatin of the P_*tub*_-lexA-BAP170- and P_*BAP170*_-lexA-SAYP-containing lines was digested normally with DpnII and efficiently ligated after that (Figure 3B), whereas the chromatin of the line containing only the P_*lexAop-hsp70*_-lacZ transgene was inefficiently digested with DpnII in parallel experiments, leading to poor ligation in the case of this line (Figure 3C, left and central panels). Halving the concentration of the fixing agent did not significantly improve digestion and ligation (Figure 3C, right panel). Moreover, the yields of DNA and especially total RNA were significantly reduced in the case of the P_*lexAop-hsp70*_-lacZ-only line as compared with the driver-containing lines (compare Figure 3B, two left and two right panels). We concluded that the genetic background prevents efficient digestion and ligation of chromatin in the case of the P_*lexAop-hsp70*_-lacZ-only line. It is possible that the RE site is methylated at A or C in this line and its digestion is thus blocked (Kunert et al., 2003; Zhang et al., 2015; Deshmukh et al., 2018), but this assumption requires further study. Thus, these important observations suggest that the genetic background can influence the success of the 3C procedure with different fly lines. To overcome the difficulties with the genetic background of the control P_*lexAop-hsp70*_-lacZ line, we used meiotic crossing over between chromosomes as a powerful genetic technique for separating harmful mutations and diluting the genetic background (see a recombination scheme in Supplementary Figure S13). Two independent lines carrying the P_*lexAop-hsp70*_-lacZ reporter transgene in the presence of the P_tub_-lexA control driver were obtained. The presence of both transgenes in crossover flies was confirmed by PCR (Figure 3E). Then 3C libraries were prepared in parallel for each new control line. Representative examples of the resulting 3C libraries are given in Figure 3F.

**FIGURE 4:**
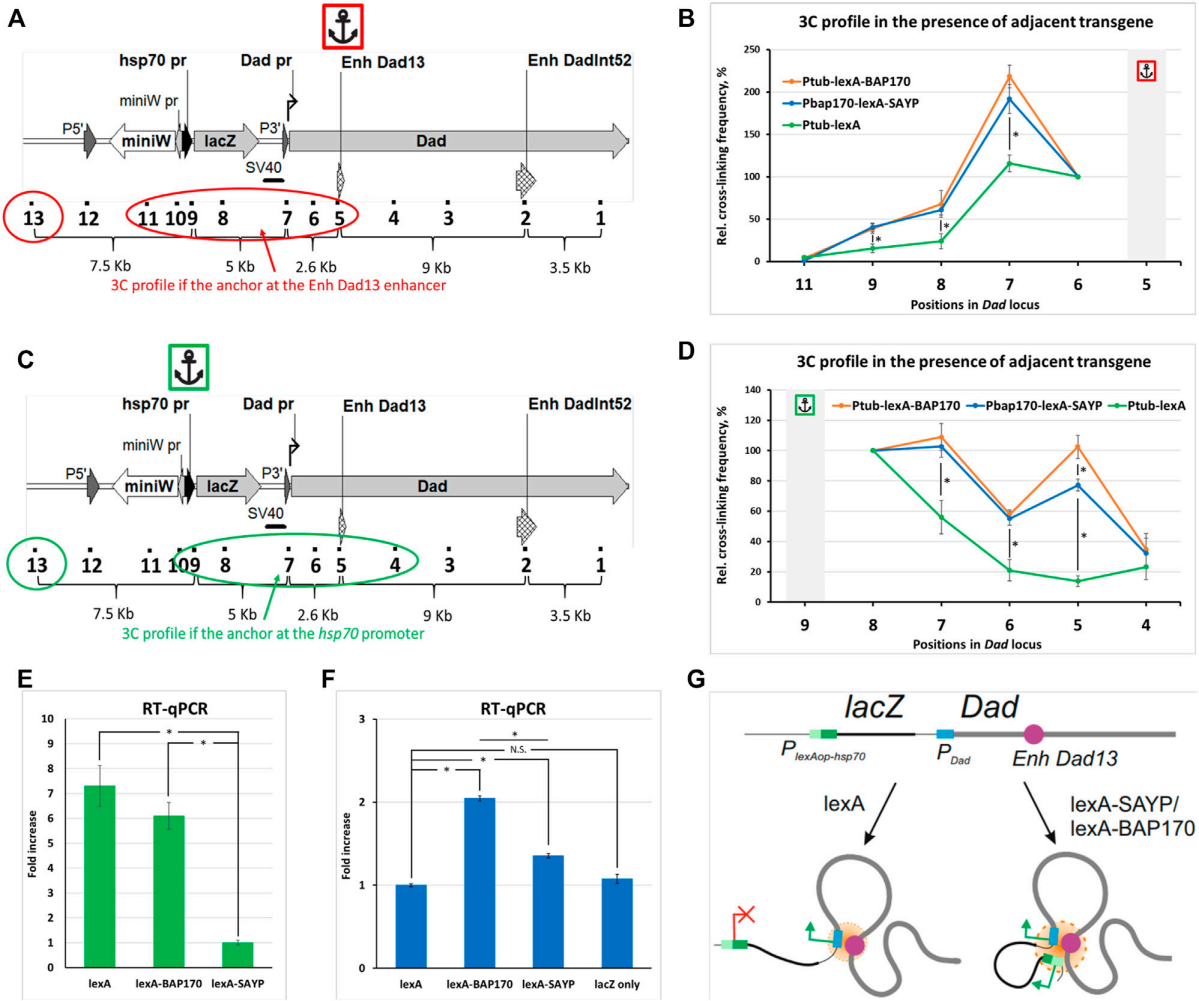
Comparison of the 3C profiles in the *Dad* locus in the presence of an adjacent transgene. **(A)** The model locus *Dad* with the adjacent transgene is shown. Designations are as shown in Supplementary Figures S3A and S6. A red circle and oval encompass the points under study. An anchor icon indicates the location of the anchor primer at the *Dad13* enhancer. **(B)** Interaction profiles for indicated positions in *Dad* locus with the adjacent reporter transgene were obtained in the presence of the P_*tub*_-lexA-BAP170, P_*BAP170*_-lexA-SAYP, or control P_*tub*_-lexA driver transgene with the anchor at the *Dad13* enhancer. Extraction, collection, and processing of larvae and 3C library preparation were done as shown in Figure 2B for larvae except that 150 larvae were collected and 50 mg of larval cell material was taken for 3C library preparation. Seven independent 3C libraries were prepared and analyzed for P_*tub*_-lexA-BAP170 and P_*BAP170*_-lexA-SAYP larvae and 4, for control P_*tub*_-lexA larvae (two for each crossover line #2 and #6, respectively). The 3C experiment was done as shown in Figure 2C except that error bars indicate SEMs from 7 or 4 independent biological replicates of the 3C library for each case, respectively. Each experimental point for each 3C library was studied in 2–4 technical PCR replicates, and the data were averaged. Point 10 was excluded from the analysis since points 9 and 10 are adjacent. The RLFs of experimental samples were normalized to RLFs within the constitutively expressed *RpII* locus and to point #13 (intergenic spacer). The values between the two normalizations were averaged. An asterisk indicates the significance level: *p < 0.05, *n* = 12. **(C)** The same as shown in **(A)**, but the anchor is at the P_*lexAop-hsp70*_-lacZ promoter and the points under study are encompassed in green. **(D)** The same as shown in **(B)**, but the anchor is at the P_*lexAop-hsp70*_-lacZ promoter. The number of the prepared and analyzed 3C libraries is the same as shown in **(B)**. The frequency of ligation of the anchor fragment with the adjacent fragment (point #8) was arbitrarily taken as 100%, and the values of all other experimental points were calculated proportionally. An asterisk indicates the significance level: *p < 0.05, *n* = 12. **(E)** RT–qPCR analysis of the expression level of the driver transgenes in mutant larvae. mRNA expression of P_*tub*_-lexA-BAP170, P_*BAP170*_-lexA-SAYP, and control driver P_*tub*_-lexA was studied using primers annealing to the LexA domain-coding sequence. The following genotypes were studied: P_*lexAop-hsp70*_-lacZ, P_*tub*_-lexA-BAP170; P_*lexAop-hsp70*_-lacZ, P_*BAP170*_-lexA-SAYP; P_*lexAop-hsp70*_-lacZ, P_*tub*_-lexA. RT–qPCR was carried out as shown in Figure 2E except that only the ΔΔCt method was used for calculation and four independent total RNAs were prepared for each driver line. The expression level in the line carrying the P_*lexAop-hsp70*_-lacZ reporter and the P_*BAP170*_-lexA-SAYP driver was arbitrarily taken as 1. An asterisk indicates the significance level: *p < 0.05, *n* = 8. **(F)** RT–qPCR analysis of the expression level of the reporter transgene *lacZ* in mutant larvae. RT–qPCR was done as shown in **(E)**. The following genotypes were studied: P_*lexAop-hsp70*_-lacZ, P_*tub*_-lexA; P_*lexAop-hsp70*_-lacZ, P_*tub*_-lexA-BAP170; P_*lexAop-hsp70*_-lacZ, P_*BAP170*_-lexA-SAYP, and P_*lexAop-hsp70*_-lacZ only. RT–qPCR was done as shown in Figure 2E except that only the ΔΔCt method was used for calculation and 4 independent total RNAs were prepared for each line. The expression level in the line carrying the P_*lexAop-hsp70*_-lacZ reporter and P_*tub*_-lexA driver was arbitrarily taken as 1. N.S., non-significant (*p* > 0.05). An asterisk indicates the significance level: *p < 0.05, *n* = 8. **(G)** A model illustrating the role of the tethered (t) SAYP/BAP170 in distal interactions in the *Dad* locus carrying an adjacent transgene. The endogenous *Dad* promoter is regulated by the endogenous Dad enhancer, the two elements always forming a contact. In the absence of tSAYP/BAP170, the transgene does not establish contact with the *Dad* gene. Recruitment of SAYP/BAP170 to the reporter promoter induces its recruitment into a joint regulatory hub with the *Dad* enhancer and *Dad* promoter.

***Recruitment of SAYP and BAP170 to the reporter promoter induces its convergence to the endogenous enhancer***. To verify that LexA-BAP170/LexA-SAYP-mediated activation of the *lacZ* reporter is accompanied by spatial convergence of the captured enhancer and the reporter gene promoter, we compared the 3C profiles of the *Dad* locus with the adjacent P_*lexAop-hsp70*_-lacZ transgene in the control line, in the presence of the P_*tub*_-lexA driver, with the 3C profiles obtained for the two experimental lines, in the presence of the P_*tub*_-lexA-BAP170 or P_*BAP170*_-lexA-SAYP driver. We observed that the ligation frequency between the *lacZ* reporter promoter and *Dad* enhancer in the control line in the presence of P_*tub*_-lexA driver transgene was significantly lower than in lines with the LexA-SAYP/BAP170 drivers, either in direct (the anchor primer was located on the *Dad* enhancer) (Figures 4A and B) or in reciprocal (the anchor primer was located on the reporter transgene promoter) (Figures 4C and D) experiments. We concluded that transcription activation mediated by the tethering of the Brahma complex subunit SAYP or BAP170 to the reporter promoter is accompanied by spatial convergence of the captured enhancer and the reporter *lacZ* gene promoter. This correlates well with a *Dad*-like expression pattern of *lacZ* in the presence of the LexA-BAP170 or LexA-SAYP (Shidlovskii et al., 2021).

***Increased expression of the driver is accompanied by an increase in the ligation frequency of the enhancer with the promoter and enhanced reporter expression***. When comparing the 3C profiles of the P_*tub*_-lexA-BAP170- and P_*BAP170*_-lexA-SAYP-containing driver lines in a direct experiment, no significant differences were found between the lines and, in both the lines, the strongest interaction of the *Dad13* enhancer was observed with the endogenous *Dad* promoter, which is closer to the *Dad13* enhancer, than the *lacZ* promoter is (Figures 4A and B), much the same as in WT larvae (Figure 2C). When comparing the 3C profiles of the P_*tub*_-lexA-BAP170 and P_*BAP170*_-lexA-SAYP lines in a reciprocal experiment, a significant difference in the ligation frequency of the *lacZ* promoter and *Dad13* enhancer was found between the lines (point #5). The difference might result from different expression levels of the driver under the control of different promoters, P_*tub*_ (constitutive strong promoter of the *α1-tubulin* gene) or P_*BAP170*_ (−373/+135 bp). To check, we measured the abundance of the driver mRNA in the P_*tub*_-lexA-BAP170 and P_*BAP170*_-lexA-SAYP lines and the control P_*tub*_-lexA line by RT-qPCR. We observed that the expression of P_*tub*_-lexA-BAP170 was indeed significantly higher than that of P_*BAP170*_-lexA-SAYP and was comparable to that in the control P_*tub*_-lexA line (Figure 4E). Thus, there is a direct relationship between the level of lexA-SAYP/BAP170 drivers and the ligation frequency of the *lacZ* promoter with the *Dad13* enhancer.

To verify that the difference in expression of the P_*tub*_-lexA-BAP170 and P_*BAP170*_-lexA-SAYP transgenic constructs results in differences in expression of the *lacZ* reporter gene, we measured the *lacZ* mRNA level in the P_*tub*_-lexA-BAP170/P_*BAP170*_-lexA-SAYP lines, the control P_*tub*_-lexA-containing line, and the P_*lexAop-hsp70*_-lacZ only line. Indeed, transcriptional activation of *lacZ* correlated with the driver type: significant differences in *lacZ* mRNA expression level were found between the P_*tub*_-lexA-BAP170 and P_*BAP170*_-lexA-SAYP lines (Figure 4F). Both P_*tub*_-lexA-BAP170 and P_*BAP170*_-lexA-SAYP driver lines differed in *lacZ* expression from the control P_*tub*_-lexA-containing line, and no significant difference was found between the P_*tub*_-lexA-containing line and the P_*lexAop-hsp70*_-lacZ only line. The control line, which carried only the P_*lexAop-hsp70*_-lacZ transgene, showed almost no activation of transcription (Figure 4F). We concluded that greater expression of the driver results in more pronounced upregulation of reporter gene expression. At the same time, low expression of the P_*BAP170*_-lexA-SAYP driver was enough to induce interactions between the *lacZ* reporter promoter and the *Dad13* enhancer.

Thus, recruitment of the SAYP/BAP170 subunit to the reporter gene promoter mediates its interaction with the enhancer, and this is accompanied by changes in chromatin fiber conformation.

We hypothesized that the activation of expression is apparently accompanied by the association of compatible (co-regulated in our case) regulatory elements into a complex (chromatin hub), which probably shares transcription factors and other common components of the transcription machinery (Figure 4G). Our data also suggest that recruitment of the PBAP chromatin remodeling complex to the promoter may be a prerequisite for establishing the interaction with enhancers in *Drosophila*.

## 3 Discussion

The principles of developmental gene functioning are an important problem in current biology. The expression of developmental genes is tightly linked to their conformation. Conformational changes in developmental genes can proceed simultaneously with the activation of their expression (Schoenfelder et al., 2015a; Bonev et al., 2017; Freire-Pritchett et al., 2017; Rubin et al., 2017; Schoenfelder et al., 2018b; Novo et al., 2018), or a specific conformation can pre-exist and serves as a scaffold to facilitate the activation (Jin et al., 2013; Ghavi-Helm et al., 2014; Schoenfelder et al., 2015b; Bonev et al., 2017; Cruz-Molina et al., 2017; Rubin et al., 2017; Comoglio et al., 2018; Ing-Simmons et al., 2021). Interactions between *cis*-regulatory elements in both cases can be investigated using 3C-based methods. In the present work, we investigated the 3C procedure for whole *Drosophila* larvae using a developmental gene *Dad* (daughters against *decapentaplegic*) as a model. We give a complete description of the 3C experiment on *Drosophila* larvae, including isolation of larvae from fly food, their sorting by age, fixation, homogenization, preparation of cellular material, preparation of experimental 3C and control libraries, analysis of interactions, and calculation of the experimental results.

### 3.1 The 3C Procedure for Whole Larvae and a *Dad* Gene Model

The 3C protocol has previously been optimized with S2 cells (Bylino et al., 2021). Here, we applied the procedure to *Drosophila* 3^rd^ instar larvae. The steps that we adapted from the protocols previously developed for cultured cells and zygotes of mammals (Rao et al., 2014; Flyamer et al., 2017; Golov et al., 2020b) (see Supplementary Figure S6 legend) also proved applicable to *Drosophila* primary cells obtained from whole 3rd instar larvae. The quality of the resulting 3C libraries was acceptable, as demonstrated by electrophoresis (Figure 2B and Figures 3B and F). To further validate our procedure, we used it to study the conformation and distal interactions between regulatory elements in the developmental locus *Dad* in WT larvae. We observed that the conformation differed between cultured S2 cells (a repressive conformation) and live larvae (an active conformation) (Figure 2D), and this observation correlated well with *Dad* expression level in larvae and S2 cells (Figure 2E). The *Dad* gene codes for a negative feedback regulator of the BMP/Dpp/TGF-β signaling pathway. Its low expression in S2 cells is in good agreement with the literature data that S2 cells produce a weak response to treatment with the Dpp ligand since these cells do not express some components of the BMP/Dpp/TGF-β pathway (Cherbas et al., 2011; Neal et al., 2019). Low or altered expression of developmental genes in cultured cells is a general problem typical not only for *Drosophila* but also for mammals and can be overcome at least partly by using primary cell cultures (Antequera et al., 1990; Kitsis and Leinwand, 1992; Zaitseva et al., 2006).

### 3.2 Enhancers of the *Dad* Locus

The *Dad* locus contains two functional enhancers: *Dad13* is the first enhancer in the chain (a primary enhancer) and the *DadInt52* enhancer downstream (a shadow enhancer) (Weiss et al., 2010). The *Dad13* enhancer is thought to be the primary element that drives the expression of the gene (Weiss et al., 2010; Neal et al., 2019). According to our data, a two-humped curve is absent in the graph in Figure 2C, when an anchor is on the *Dad13* enhancer, indicating that the *Dad13* enhancer is not in complex with the *Dad* promoter and *DadInt52* enhancer. This is in good agreement with the leading role that the first enhancer in a chain of gene enhancers plays in regulating expression in higher eukaryotes and their evolutionary precursors (Song et al., 2019; Bylino et al., 2020). The finding additionally correlates well with observations by Weiss et al. (2010) and Neal et al. (2019), who showed that the *Dad13* enhancer provides a proper transcriptional response and drives the expression of the reporter gene in a pattern virtually identical to that of endogenous *Dad*, while *DadInt52*-driven expression is weak and only partially overlaps that of *Dad13*. Altogether, our data emphasize that the *DadInt52* enhancer is a shadow enhancer and plays an auxiliary or redundant role in comparison with the *Dad13* enhancer. However, it cannot be ruled out that *DadInt52* is involved in the regulation at a developmental stage other than embryos (Weiss et al., 2010) or 3rd instar larvae (this work). Our results concerning the leading role of the first enhancer are in good agreement with the results by Bieli et al. (2015), who have studied the enhancer–promoter interactions in whole larvae for the developmental gene *apterous.* They have similarly found that the *apterous* enhancer closest to the promoter (the first in the chain) interacts with the promoter more strongly than the second enhancer (the second in the chain). Moreover, the leading enhancer interacted with the promoter more strongly than with the control region situated downstream of the *apterous*, like in our case (point #13). This suggests that the mode of regulation is universal for developmental genes and emphasizes the leading role of the first enhancer in a chain of enhancers.

### 3.3 3C Experiment With WT and Mutant Larvae

Having studied the interactions in cultured cells and WT larvae, we applied our procedure to the mutant fly lines that carried a reporter transgene adjacent to the *Dad* gene and demonstrated conformational changes in the *Dad* locus upon activation of transgene expression (Figures 4B and D). The induction of expression was achieved through the recruitment (targeted tethering) of SAYP or BAP170 subunits of the SWI/SNF remodeling complex to the reporter promoter and subsequent assembly of the SWI/SNF complex (Shidlovskii et al., 2021). Recruitment of SAYP/BAP170 to the *lacZ* promoter leads to the discrimination of this promoter among the other promoters of the transgene (P-element promoter and *mini-white* promoter) and specific activation of *lacZ* expression following a *Dad*-like pattern (Shidlovskii et al., 2021), thus, providing an example of specificity of enhancer–promoter communication (Galouzis and Furlong, 2022). Accordingly, we hypothesized that the targeted recruitment of the specific SWI/SNF subunits to the promoter is accompanied not only by an increase in gene expression but also by spatial convergence of the regulatory elements. We observed that this was the case (Figures 4B and D). At the same time, surprisingly, the recruitment of the SAYP/BAP170 resulted in more pronounced interactions of the *Dad13* enhancer not only with the reporter promoter but also with the *Dad* promoter (Figure 4B) and enhanced interaction of the two promoters with each other (Figure 4D). We hypothesized that both promoters and the *Dad13* enhancer combine to form a ternary complex (chromatin hub), which possesses a more stable conformation compared with a dual *Dad* promoter-enhancer complex and provides a basis for a higher ligation frequency (Figure 4F). Alternatively, increased ligation between regulatory elements may be interpreted as an activation chromatin hub, where enhancement of both promoter activation might take place due to the looser chromatin structure induced by targeted recruitment of SAYP/BAP170 and subsequently of Brahma complex. However, we do not know whether the *Dad13* enhancer activates both promoters simultaneously or sequentially or whether this activation occurs in different cells [discussed in (Peng and Zhang, 2018)]. The first scenario is supported by many observations of the co-localization of regulatory elements of co-expressed genes within the same transcription factory (Osborne et al., 2004; Mitchell and Fraser, 2008; Sutherland and Bickmore, 2009; Rieder et al., 2012). Such genes usually share the components of the transcription apparatus, and co-expression of two transgenes/endogenes closely located in the genome and regulated with the same enhancer and competition between two promoters for one enhancer is well-documented in the literature (Raj et al., 2006; Papantonis et al., 2010; Bartman et al., 2016; Fukaya et al., 2016; Lim et al., 2018; Stavreva et al., 2019).

### 3.4 Competition Between Two Promoters for the Same Enhancer

Competition between the *lacZ* and *Dad* promoters can also occur in order to establish separate contacts with the enhancer. Indeed, mutually inhibitory co-expression of genes has been demonstrated when two different sequentially located globin genes are regulated by one enhancer (LCR) *in cis* (Deng et al., 2014; Bartman et al., 2016). It strongly resembles the organization of the *Dad* locus under study. In this model, one would expect an “enhancer hijacking” phenomenon. In the case of hijacking of the *Dad13* enhancer by the transgenic promoter, the 3C profile would show a decrease in interaction with the endogenous *Dad* promoter, which would be accompanied by a corresponding increase in interaction with the transgenic promoter. However, it was not detected. Moreover, in our case, *lacZ* is apparently expressed at a significantly lower level than the *Dad* gene (data not shown). Due to this, it is not possible to reliably determine by RT-qPCR whether an increase in *lacZ* expression in the presence of SAYP/BAP170 is accompanied by a corresponding decrease in *Dad* endogene expression. The presence of the *lacZ* transgene has just a slightly negative effect on *Dad* expression (data not shown) and may be caused by disruption of *Dad* promoter structure (hypomorphic mutation) rather than by enhancer hijacking. Thus, our data indicate that, even if the reporter promoter hijacks some of the *Dad13* enhancer activity, it does so in a very weak manner.

### 3.5 Activation of RNAPII Promoter by RSC/PBAP/PBAF Complex Depends on Natural Genomic Context

Our previous data showed that targeted recruitment of the SAYP/PHF10 PBAP signature subunit fused with GAL4 DBD to the *hsp70* promoter [−259/+198, containing GAF and six HSF sites (Ingolia et al., 1980; Steller and Pirrotta, 1984, 1985; Weber et al., 1997; Wu et al., 2001; Kwak et al., 2013)] under the control of UAS strongly induces reporter gene expression in a PBAP-dependent manner (Vorobyeva et al., 2009). These experiments were performed with a transient transfection model and a model of a reporter transgene integrated into the genome in multiple copies in a *Drosophila* S2 cell culture. In our experiments described in this work and in the study by Shidlovskii et al. (2021), the upstream LexA binding sites were combined with the minimal *hsp70b* core promoter (−45/+207 bp), lacking upstream GAF and HSF binding sites, and this reporter transgene was placed in genomic context in a single copy. Targeted recruitment of the SAYP/BAP170 subunits to the promoter was insufficient for reporter activation, required an active enhancer to occur in the genomic vicinity, and was accompanied by the convergence of the enhancer and the *hsp70* promoter. Although the *hsp70* promoters used in the previous and current models differ in length, our data may point to the importance of the genomic context: a reporter located in a plasmid or in the genome may show different behaviour. Apparently, the chromatin environment and its 3D conformation make a significant contribution to the reporter gene activity.

Previously, the effect of targeted Brahma subunit recruitment on gene activity was tested in yeast and human cells. Recruitment of the RSC/PBAP/PBAF complex subunits Sth1 or Swh3/Rsc8 (MOR/BAF155/170) to the core promoter did not induce expression of the RNAPII promoter (Laurent et al., 1992; Treich and Carlson, 1997), whereas recruitment of the ySWI/SNF/BAP/BAF complex subunits SNF2, SNF5, SNF6, and SNF11 to the same promoter sufficed to induce expression (Laurent et al., 1990, 1991; Laurent et al., 1992; Treich et al., 1995). Thus, these data may indicate that different roles are played by the ySWI/SNF and RSC complexes in RNAPII promoter regulation in yeast. Subunits of the human BAF complex, hBRM and hDPF3a (D4/TTH/BAF45B/C/D), induced expression of several RNAPII promotes upon their targeted recruitment in human cell cultures (Muchardt and Yaniv, 1993; Trouche et al., 1997; Cui et al., 2016). All of these promoters possess a long enhancer/promoter region, and their BAF-driven activation depends on cell activators. However, the reporter was in a plasmid in all of these cases; therefore, the data cannot provide direct evidence that the test proteins act similarly in the natural genomic context.

Genome-wide data show that BAF occupies enhancer regions, while PBAF is found on both enhancers and promoters (Nakayama et al., 2017; Carcamo et al., 2022). We also found that PBAP occupies both *Dad* enhancer and promoter (Shidlovskii et al., 2021). We hypothesized that, in addition to the catalytic function, PBAP/PBAF mediates interactions between enhancers and promoters in a non-catalytic mode (Kwok et al., 2015; Jordan-Pla et al., 2018).

### 3.6 Role of SWI/SNF in Establishing Long-Range Contacts Between Enhancers and Promoters

Since the targeted recruitment of the SWI/SNF subunits to the reporter promoter and its activation is accompanied by changes in the conformation of the chromatin fiber, we hypothesized that SWI/SNF may facilitate the organization of long-range regulatory gene contacts in the interphase in *Drosophila*. This mechanism of gene regulation might be basic, more ancient, and more universal than the regulation *via* cohesin-dependent loop extrusion, which is absent in *Drosophila* (Rowley et al., 2017; Eagen, 2018; Gambetta and Furlong, 2018; Matthews and White, 2019; Bylino et al., 2020; Kaushal et al., 2021). The hypothesis about the role of SWI/SNF in the spatial regulation of gene expression is strongly supported in the literature. For example, loop formation mediated by the SWI/SNF complexes was found in microscopic (Bazett-Jones et al., 1999), real-time *in vitro* (Lia et al., 2006; Zhang et al., 2006; Sirinakis et al., 2011), biochemical, and genetic studies (Ni et al., 2008; Kim et al., 2009a, Kim et al., 2009b; Shi et al., 2013; Jégu et al., 2014). Even in mammals, where cohesin brings together distant regions of immunoglobulin genes, regulating the switching of types of antibodies by the loop extrusion mechanism (Thomas-Claudepierre et al., 2013; Zhang et al., 2019), it has been shown that SWI/SNF assists this process (Kwon et al., 2000; Bossen et al., 2015). The interplay between SWI/SNF and CTCF on their DNA binding sites has also been detected (Euskirchen et al., 2011; Barutcu et al., 2016; Wood et al., 2016); similar data have been reported for condensin II and SWI/SNF (Wu et al., 2019). Thus, in organisms where loop extrusion appears to be the main regulatory mechanism of distant interactions, SWI/SNF also contributes to the organization of this type of regulation. Our data indicate that SWI/SNF is necessary for establishing enhancer–promoter communication in *Drosophila*.

## 4 Conclusion

(i) The 3C procedure optimized using S2 cells as a model can be successfully applied to studying the chromatin conformation and interactions between regulatory elements of developmental genes in living *Drosophila* larvae.
(ii) Whole larvae can be used to study not only ubiquitous intragenomic interactions but also tissue-specific ones. Our results indicate that, even if an interaction is specific for a limited set of cells in larvae, the 3C procedure using whole larvae allows its quantitative measurement and comparison in different lines.
(iii) The genetic background may affect the overall DNA yield and digestion with a restriction enzyme in the 3C procedure.
(iv) The model locus *Dad* exists in different conformations in 3rd instar larvae and cultured S2 embryonic cells. The active conformation correlates with the transcriptional activity of the gene in living larvae.
(v) Targeted recruitment of SAYP and the BAP170 subunits (SWI/SN and PBAP) to a reporter promoter induces the convergence of the promoter and endogenous enhancer. This is accompanied by an increase in reporter gene expression.

## Data Availability

The raw data supporting the conclusion of this article will be made available by the authors, without undue reservation.

## Abbreviations

3С, Chromosome Conformation Capture; BAC, bacterial artificial chromosome; BAP170, Brahma-associated protein 170 kD; BMP, bone morphogenic protein; DTT, dithiothreitol; DBD, DNA binding domain; EB, extraction buffer; EDTA, ethylenediaminetetraacetic acid; FA, formaldehyde; qPCR, quantitative polymerase chain reaction; PBS, phosphate-buffered saline; FA, formaldehyde; PFA, paraformaldehyde; Ph/Chl, Phenol/Chloroform; PHF10, PHD finger protein 10; PIC, protease inhibitor cocktail; RB, restriction buffer; RE, restriction enzyme; RLF, relative ligation frequency; RNAPII, RNA polymerase II; RT, room temperature; RT-qPCR, reverse transcription-quantitative PCR; S2 cells, Schneider 2 cells; SAYP, supporter of activation of yellow protein; SDS, sodium dodecylsulfate; TGF-β, Transforming growth factor beta; WT, wild type.
