# Supplementary material for: Application of the 3C Method to Study the Developmental Genes in Drosophila Larvae
Source: Front Genet. 2022 Jul 15;13:734208. doi: 10.3389/fgene.2022.734208 (PMC9335292; doi:10.3389/fgene.2022.734208)
Supplement: Supplementary file 1 [file DataSheet1.pdf]

Supplementary Materials for the paper

Application of the 3C Method to Study the Developmental Genes in Drosophila Larvae

by Oleg V. Bylino, Airat N. Ibragimov, Filomena Anna Digilio, Ennio Giordano, Yulii V. Shidlovskii

1 Materials and methods

Drosophila cell culture and strains

Cultured S2 *Drosophila melanogaster* embryonic cells (Sg4 clone) were kindly provided by Dr. N.E. Vorobyeva and were maintained in the SFX-Insect medium (HyClone) in the presence of penicillin/streptomycin (HyClone). The Canton S isogenic wild-type fly strain was from the Bloomington collection (USA). The lines containing $P_{lexAop\text{-}hsp70}$-lacZ reporter transgene in the presence of either P_{tub}-lexA-BAP170 or P_{BAP170}-lexA-SAYP transgene were described in (Shidlovskii et al., 2021). The control line containing $P_{lexAop\text{-}hsp70}$-lacZ combined with P_{tub}-lexA was obtained by recombination for this work.

Processing of larvae extracted from fly food and preparation of cell material

The sieves with larvae were extensively washed with tap water of RT, and 3rd and 2nd instar larvae were picked up with a spatula with a groove from the 315-µm sieve into 100-µm Cell Strainer Nylon (Corning Falcon, cat. no. 352360). The larvae were washed on Cell Strainer with the EW buffer (137 mM NaCl (0.8%), 0.05% Triton X-100) and transferred with a paintbrush from Cell Strainer into a 7-ml Dounce homogenizer containing 0.5 ml of 1X NU-1 supplemented with 2 mM DTT and 2X PIC (Roche Complete EDTA-Free Protease Inhibitor Cocktail). Then, 0.5 ml of 1% FA in 1X NU-1 was added to the homogenizer and the countdown was started (it was found in preliminary experiments that the FA concentration is much more important than the fixation time; fixation with 0.5% PFA for 10 min provided the best digestion with DpnII in 3C (Bylino et al., 2021)). Homogenization was carried out simultaneously with fixation (Comet et al., 2011). To make a proper homogenate, 30 strokes with pestle A were done (until homogenization with the pestle is easy). The homogenate was poured into 40-µm Cell Strainer Nylon (Corning Falcon, cat. No. 352340) placed in a 50-ml tube, and the pestle and homogenizer were washed inside first with 0.5 ml of 1X NU-1 with 2 mM DTT and 2X PIC and then with 0.5 ml of 1% PFA in 1X NU-1 (when a 15-ml Dounce is used, the volumes of all solutions should be doubled). The washing was poured into another 40-µm Cell Strainer placed in a 50-ml tube. Both tubes were centrifuged at 3,220 g for 1 min. The pellet in one 50-ml tube was resuspended by vortexing and poured into the second 50-ml tube, and the pellet in the

second tube was also resuspended by vortexing. Then, the volume of 1X NU-1 supplemented with 1 mM DTT, 1X PIC, and 0.5% FA was adjusted to 5 ml (+~3.5 ml), and larval cell material was incubated in the presence of 0.5% PFA with tumbling for up to 10 min. Glycine was added in an equimolar amount or a slight excess relative to FA (Comet et al., 2011; Sexton et al., 2012) (but no more than 400 mM glycine vs. 333 mM FA reactive groups). We proceeded from the fact that FA has two reactive groups and glycine has one. The cell material was incubated with tumbling at RT for 5 min. An equal volume of ice-cold 1X PBS was added, and the cell material was centrifuged at 2,500 g at +4 °C for 3 min (no dense pellet will form without 1X PBS addition). The supernatant was discarded, and the pellet was resuspended by vortexing in 10 ml of ice-cold 1X PBS and centrifuged 2,500 g at +4 °C for 2 min. The washing was discarded, and the pellet was resuspended in 1 ml of ice-cold 1X PBS, transferred to a 1.5-ml tube, and centrifuged at 5,000 g for 2 min. The supernatant was removed, and the pellet was weighed to determine the amount of starting cell material and resuspended in 1 ml of ice-cold 1X PBS.

Genomic DNA isolation from fly lines for screening of crossover flies

For screening, one fly from each line was homogenized at RT using Eurostar power-b, IKA-WERKE with 1.5 ml Pellet Pestle Rod using Eurostar power-b, IKA-WERKE with 1.5 ml Kontes microtube pellet pestle rod (#EF2486B749521-1500) in 300 µl of a buffer of the following composition: 10 mM Tris-HCl, pH 8.0, 25 mM NaCl, 1 mM EDTA. PrK was added up to 200 µg/ml, and the samples were incubated at 37 °C for 30 min. To inactivate PrK, the samples were incubated at 95 °C for 2 min, and DNA was extracted with Ph/Chl, precipitated, and washed with ethanol as described in Fig. 1D legend. Then, DNA was dissolved in 50 µl of 50 mM Tris-HCl, pH 8.0. 0.25 µl of the DNA solution was taken for PCR. PCR conditions were as follows: number of PCR steps, 3 (94° 10 sec, 60° 20 sec, 72° 15 sec); 30 cycles; concentration of primers was 0.2 pM/µl each; 2 mM $MgCl_2$ in the PCR reaction mixture.

Genomic DNA isolation from S2 cells and flies for calibration curve preparation

Thirty flies were homogenized as in the previous section in 300 µl of the EB buffer (50 mM Tris-HCl, pH 8.0, 50 mM NaCl, 1% SDS) (Hug et al., 2017). Proteinase K was added to 0.2 mg/ml; the samples were incubated at 56 °C for 30 min; and DNA was extracted, precipitated (w/o glycogen), and washed with ethanol as described in the Fig. 1D legend. DNA was dissolved in 150 µl of Tris-HCl, pH 7.9, treated with 50 U of RNase I (Thermo) at RT for 30 min, purified using 1.5X AMPure XP beads, and eluted with 150 µl of 10 mM Tris-HCl, pH 7.9. To isolate DNA from S2 cells, 100 mg of cells were harvested, washed with 1X PBS, and resuspended in 1 ml of EB. Proteinase K was added to 0.2 mg/ml, and the samples were incubated and treated as above. The amount of DNA was estimated using a Qubit dsDNA HS kit.

Preparation of a fixing agent

4% FA (aqueous solution) was obtained from dry paraformaldehyde (PFA) (MP Biomedicals #150146) as described in (Bylino et al., 2021).

Preparation of solutions for homogenization of larvae

Larvae are homogenized in 0.5 % PFA in 1X NU-1 (0.350 mM sucrose, 20 mM KCl, 10 mM $MgCl_2$, 0.2 mM EDTA, 1 mM EGTA) supplemented with 1 mM DTT (1 M DTT stock solution is filtered through a 0.22-µm syringe filter and kept at –20 °C) and 1X PIC (100X PIC stock solution is prepared by dissolving 1 tablet of cOmplete Roche (#11873580001) in 500 µL of water sterilized by autoclaving). This working solution is convenient to obtain by combining 1:1 1% PFA solution in 1X NU-1 and 1X NU-1 supplemented with 2 mM DTT, 2X PIC. 1% PFA solution in 1X NU-1 is obtained by combining 1:1 2% PFA solution in water with 2X NU-1. Hence, 2X NU-1 stock solution is first prepared. The 2X NU-1 solution is prepared from 1M stock salt solutions sterilized by autoclaving. 0.5M EDTA, EGTA, and 2M sucrose solutions are sterilized by filtering through Corning 250 mL Vacuum Filter/Storage Bottle System (#431096). Alternatively, the 2X NU-1 solution can be prepared from unsterilized fresh stock solutions of salts, EDTA, EGTA, and sucrose and filtered afterwards through the above system.

Larvae to be homogenized are first transferred to 7 ml Dounce homogenizer containing 0.5 ml of 1X NU-1 solution supplemented with 2 mM DTT and 2X PIC, and then 0.5 ml of 1% PFA in 1X NU-1 is added to the larvae. After homogenization, the pestle and homogenizer are consecutively washed with 0.5 ml of the former and 0.5 ml of the later solution. In the case of using a 15-ml Dounce, the volumes should be doubled. It is convenient to prepare 10 ml of 1X NU-1 supplemented with 2 mM DTT and 2x PIC and 10 ml of 1% PFA in 1X NU-1 for the work.

Primer and probe design

TaqMan probes and anchor and tester primers (Table S1) were designed against the ends of ligated fragments within 100 bp from the DpnII cutting site so that the anchor primer and probe hybridize to opposite strands of the anchor fragment (Splinter et al., 2006). The TaqMan probes were labeled with FAM at the 5' end, and one T from 10 to 16 nucleotides of each probe was labeled with BHQ1.

qPCR

qPCR was performed in 4 replicates with 3C-library DNA, 0.3 µM of each primer, 0.3 µM Taq-man target probe, 1 U of Hot Start Taq DNA pol (Sibenzyme), 0.2 mM dNTP, and 2 mM $MgCl_2$ in 10-µl volumes on a CFX 96 touch machine (Bio-Rad) with the following program: initial denaturation at 94°C for 1 min followed by 45 cycles of 94°C for 10 s and 60°C for 1 min.

3C products were quantified relative to a control template (a random ligation library), which was made from a Sau3A (BspI) (Thermo) digest of an equimolar mixture of BAC clone #CH321-43A11 (BACPAC Genomics) overlapping the *Drosophila Dad* genomic locus and the pCasper-AUG-βgal plasmid (Thummel et al., 1988) carrying the reporter $P_{lexAop\text{-}hsp70}$-lacZ gene (Shidlovskii et al., 2021). The relative cross-linking frequency was calculated against a calibration curve, which was obtained

from 10-fold dilutions of the random ligation library template. To correct for differences in quality and quantity of 3C templates, ligation efficiencies of experimental samples were normalized to ligation efficiencies detected between two adjacent DpnII fragments within the constitutively expressed *RpII* locus (Comet et al., 2011). The experimental design of primers and probes to estimate the regeneration efficiency (regeneration of the DpnII site) and the amount of the uncut site in the *RpII* locus is presented in the Fig. S6E. The parameters were calculated against a calibration curve, which was constructed using 10-fold dilutions of the PCR product obtained with the primers RpII_1_Forward and RpII_3_Reverse#2 (Table S1). The PCR product was purified from gel. The results were calculated in MS Excel 2019 using the exponential function equation or the trend function.

Isolation of total RNA and RT-qPCR

Total RNA was isolated using the TRI reagent (Molecular Research Center) according to the manufacturer's protocol. Instead of chloroform, I-BCP (1-bromo-3-chloropropane) was added in a proportion of 1/10 of the volume of the TRI reagent [Chomczynski, Mackey, 1995]. Thirty 3rd instar larvae or 100 mg of S2 cells were homogenized in the TRI reagent at RT using an IKA-WERKE Eurostar power-b device with a Kontes 1.5-ml microtube Pellet Pestle Rod (#EF2486B749521-1500). Then the samples were centrifuged at 15 000 rpm at 4 °C to pellet undisrupted tissues, the supernatant was transferred to new tubes, and RNA was isolated according to the manufacturer's protocol. RNA was precipitated with isopropanol and dissolved in autoclaved mQ water. To remove genomic DNA contamination from the total RNA preparation, 20 μg of total RNA was treated with 2U (1 μL) of recombinant DNase I (DNA-free Kit DNase Treatment and Removal Reagents, Thermo) in a volume of 10 μL min at 37 °C for 30. A new DNas I portion (2 U) was added, and the incubation was repeated. The treatment was carried out in a buffer with $MgCl_2$ and autoclaved mQ water. After incubation, 2 μl of the DNase Inactivation Reagent was added to the reaction to remove DNase and buffer components and incubated with stirring at RT for 2 min. Then 2 μl of autoclaved mQ water was added, the samples were centrifuged at 15,000 g for 1.5 min, and 10 μl of the supernatant was saved. Autoclaved water (10 μl) was added to the pellet, the pellet was resuspended, the samples were centrifuged at 20,000 g for 1.5 min, and 10 μl of the supernatant was taken. The supernatants were pooled, the pellet was discarded. For reverse transcription (RT), 5 μl (~ 5 μg) of DNase-treated RNA was used. This amount of RNA allowed us to detect the transcripts of tissue-specific developmental genes in RT-qPCR with ease. RT was performed with an oligo(dT) primer in a volume of 10 μl. RNA was mixed with 20 pM of the oligo(dT) primer and autoclaved water, dNTP was added to 0.5 mM, and the mixture was incubated at 65 ° C for 5 min (melting of the RNA secondary structure). Then the tubes were immediately transferred on ice (primer annealing), supplemented with 5X buffer for RT and RT (RevertAid, Thermo), and incubated at 37 ° C for 30 min–1 h. In parallel with the main RT reaction, the following controls were performed: "–RT" (the reaction without adding RT) and well as RT without the primer. The main RT reaction and the controls were diluted with autoclaved water by a factor of 5, and 1/10 of the volume (5 μl, ~500 ng cDNA) was used for PCR or for qPCR with SYBR Green I (Sigma, S9430-1ML; the final SYBR

Green concentration in the qPCR mixture was 0.5X) in a volume of 10 µl. The PCR conditions were as follows: initial melting at 94 ° C for 1 min; 35 cycles of 94 °C for 10 sec, 60 °C for 20 sec, and 72 °C for 15 sec; and final extension for 1 min 15 sec. The quality of RT and the controls after PCR was checked by electrophoresis in 1.25% agarose gel. The samples were combined with 0.4 µl of the loading buffer with the Purple dye (NEB, #B7024S) or TriTrack (Thermo, #R1161), and 5 µl of the PCR mixture was applied onto gel. To control DNA separation, 0.9 µl (0.45 µg of DNA) of the GeneRuler DNA Ladder Mix (Thermo, #SM0331) DNA marker was mixed with 0.4 µl of the Purple or TriTrack loading buffer and 13.7 µl of 10 mM Tris-HCl, pH 8.0. The mRNA level was measured by qPCR with SYBR Green I. The PCR conditions were as above. To precisely estimate the transcript abundance, a calibration curve prepared from either S2 cell DNA was performed in parallel with the samples. The following DNA concentrations in the PCR mixture were used to obtain the calibration curve: 35.4 ng, 3.54 ng, 354 pg, and 177 pg (Qubit measurement). Water and "–RT" were additionally used as controls in the qPCR experiment. The mRNA content was calculated using the ΔΔCt method (Livak and Schmittgen, 2001; Schmittgen and Livak, 2008; Rao et al., 2013).

DNA electrophoresis of 3C libraries

For 3C libraries from S2 cells, a Chromatin Integrity Control (control #1) lane should contain predominantly high-MW DNA (above 12 kb). A higher proportion of S-phase cells in the culture increases the amount of DNA fragments below 10 kb according to our observations (not shown). Chromatin Digestion Control (control #2) from S2 cells after efficient digestion with DpnII appears as a smear that starts from about 3–6 kb and has a density center around 1–1.5 kb in agarose gel electrophoresis pictures. A ligated 3C library from S2 cells appears as a smear with an intensity peak migrating at the level of 6- to 10-kb markers. A more concentrated smear at the top of a lane indicates more efficient ligation and should ideally be around 10 kb. The longer the DNA smear, the poorer is the ligation efficiency.

For 3C libraries prepared from larvae, Chromatin Integrity Control contains only DNA of high molecular weight (above 12 kb) and moves as a single band. Chromatin Digestion Control may look differently for different fly strains but should generally be shorter than 2–3 kb with an intensity peak between 500 bp and 1 kb.

The efficiency of DNA ligation for larvae is generally lower than for S2 cells, and ligated DNA more often looks like a smear with an intensity peak at about 3.5–5 kb.

DNA electrophoresis of 3C libraries was done in freshly prepared 1X TAE (of the following 50X composition: 24.2 g of Tris, 1.46 g of EDTA, and ~5.7 ml of glacial acetic acid (titrate until pH drops to 8.6)) and 1.25% agarose gels (TopVision Agarose, Thermo #R0492) prestained with ethidium bromide in the 1X TAE buffer in Helikon SE-2 electrophoresis camera at 100 V (3.7 V/cm) for 45–50 min. The width of a comb, number of teeth in the comb, and the length of each tooth were 1.0, 18, and 0.4 mm, respectively. DNA resolution was monitored using a MassRuller High range DNA Ladder (Thermo #SM0393, 1500-10 000 bp) mixed 1:1 with a MassRuler Low range DNA Ladder

(Thermo #SM0383, 80-1031 bp) or using a GeneRuler DNA Ladder Mix (Thermo #SM0331, 100–10 000 bp, bright bands are 500, 1000, and 3000 bp).

For routine quality testing of 3C libraries prepared from 10 mg of S2 cells (described in Fig. S6), 1 (for ligation and control #1) or 10 (for control #2) µl of a 3C library was typically mixed with 13.6 or 3.6 µl of 10 mM Tris-HCl, pH 8.0, and no more than 0.4 µl of Purple Gel loading dye 6X (NEB #B7024S) or TriTrack DNA loading dye (Thermo #R1161) (total volume 15 µl). To obtain final images of 3C libraries prepared from S2 cells, 200 ng of DNA was used for ligation and control #1 and 300–400 ng of DNA, for control #2 per lane and was mixed with 0.4 µl of the above-mentioned dyes and 10 mM Tris-HCl, pH 8.0, in a total volume of 15 µl (see Fig. 2B, left panel). To control DNA separation in gel, 0.9 µl of GeneRuler DNA Ladder Mix DNA marker was used and mixed as above (total volume 15 µl). For electrophoresis of 3C libraries prepared from *Drosophila* 3rd instar larvae, DNA was used at 25 ng for control #1, 50 ng for control #2, and 75 ng for ligation and mixed with no more than 0.4 µl of Purple or TriTrack loading dye and 10 mM Tris-HCl, pH 8.0, in a total volume not less than 15 µl (see Fig. 2B, right panel). To control DNA resolution in gel, half the amount of GeneRuler DNA Ladder Mix DNA marker was used (0.45 µl) and mixed as above (total volume 15 µl).

Preparation of plasmid and BAC DNA

DNA of the plasmid pCasper-AUG-beta-gal with the cloned lexAop-hsp70 promoter (Shidlovskii et al., 2021) was isolated from *E. coli* DH5α cells using a GeneJET Plasmid Miniprep kit (Thermo #K0503) according to the manufacturer's instructions. BAC DNA was prepared as described in the *Isolation of BAC* subsection.

Table S1. List of primers and TaqMan probes

Abbreviation	Destination	Sequence
Primers for 3C analysis		
1	Point 1 (*Dad* gene)	5'- CTCACACCAGCAGGCAATCA
2	Point 2 (*DadInt52* enhancer)	5'- GGGGAGAGGCAAAAGAGAGTG
3	Point 3 (*Dad* gene)	5'- CCAGAACCCAACACCCAGA
4	Point 4 (*Dad* gene)	5'- CCAGACGCAAACATAGACACC

5	Anchor primer at *Dad13* enhancer	5’- TTCCCCCTTACCAATCGTTCC
6	Point 6 (*Dad* gene)	5’- ATACGGTTTATGTGGAGGGAATGAA
7	Point 7 (*Dad* promoter)	5’- CGTCGTAAACAACAAACAAGTGC
8	Point 8 (*lacZ* ORF)	5’- ACGCCATCCCGCATCTG
9	Anchor primer at $P_{lexAop\text{-}hsp70}$-*lacZ* promoter	5’-CAAAGTGAACACGTCGCTAAGC
10	Point 10 (*mini-white* promoter)	5’- ACAGAGAAGGAGGCAAACAGC
11	Point 11 (*mini-white* CDS)	5’- GGATAGGAGTTGAGATGTAATGTAATGC
12	Point 12 (5’ end of P-element transgene)	5’- GGCTACTCCTTGCGTCGG
13	Point 13 (intergenic spacer)	5’- GGACACACACGCTACTCAGAT
5_test	*Dad13* enhancer_test primer	5’- CCCGCCCTCTTTCACCAA
9_test	$P_{lexAop\text{-}hsp70}$-*lacZ* promoter_test primer	5’- CAGACCAATGCCTCCCAGAC
5 probe	probe at *Dad13* enhancer	5’- (FAM)CCCACCATCG(T-BHQ1)CCGTCTCTTTCTCGCTG-P
9 probe	probe at $P_{lexAop\text{-}hsp70}$ promoter	5’-(FAM)TAACCAGCAACCAAG(T-BHQ1)AAATCAACTGCAACT-P
RpII_1_Forward	normalization to RpII, DpnII site regeneration	5’- GGGGCGACCAGAAGAAGGC

RpII_2_Reverse#1	normalization to RpII, DpnII site regeneration	5'-CCGCAAATGGGAAAGAGTAGAGG
RpII_3_Reverse#2	normalization to RpII, DpnII site regeneration	5'- GCGATGGCAGAAGGAGCAAT
RpII_0 probe	normalization to RpII, DpnII site regeneration	5'- (FAM)TCAAGCGAT(T-BHQ1)CAACACCTGGGAGACACCG-P
Primers for RT-qPCR		
lexA-DBD_F	Estimation of LexA fusion expression	5'- AGGGTGTCATTGAAATCGTGTCC
lexA-DBD_R		5'- CGGCTTGAACTCGCTATTCTCC
lacZ_ORF_F	Estimation of *lacZ* reporter expression	5'- GGCAGGCGTTTCGTCAGTATC
lacZ_ORF_R		5'- GCGGTAGTTCAGGCAGTTCAATC
oligo(dT)	cDNA synthesis	5'-TTTTTTTTTTTTTTTTTTTTTTTTTTTTTTVN
Dad_F	Estimation of *Dad* expression	5'- GAAGGTGTTATGGCGTTATGCG
Dad_R		5'- CGGATGGCTGTTGTTGTTGTTG
beta-tubulin 56D_F	Estimation of *tub* expression	5'- GCCACGGACGCTACCTTAC
beta-tubulin 56D_R		5'- GCCTCGGTGAACTCCATCTC

2 Preparation of the control library (random ligation library) for constructing a calibration curve

2.1 Cultivation of BAC

CHORI321 library plasmids of the pCC1BAC series (attB_P[acman]-CmR-BW) contain *Drosophila* genomic DNA inserts of ~100 kb in the EPI300 *E. coli* host cell strain (Venken et al., 2009) (Fig. S1A). pCC1BAC has two origins of replication, an F-factor origin for single-copy maintenance and OriV (the RK2 minimal replication origin) for high-copy number plasmid replication. The host strain genome contains the inducible *trfA* gene, which is required for OriV-dependent replication. The inductor of *trfA* expression is unknown. Therefore, we first examined the inductors of the two most common *E. coli* induction systems, the tetracycline and arabinose systems (Bujard and Gossen, 1997; Better, 2004). To induce the Tet promoter, we used tetracycline hydrochloride or chlortetracycline inactivated by autoclaving. After autoclaving, the inductors lose their properties to inhibit protein synthesis, but retain the properties to induce the Tet promoter (Bochner et al., 1980; de Cristóbal et al., 2006). To induce the *araBAD* operon promoter, we used L-arabinose. It was found that only the addition of L-arabinose led to an increase in BAC copy number (Fig. S1B) and was accompanied by a delay in cell growth upon induction. We concluded that *trfA* is under the control of the *araBAD* promoter.

A large amount of BAC is necessary for the preparation of a calibration curve. However, the possibility to grow in the presence of a replication inducer is limited to 2 hours because of the possible instability of BAC with a large copy number. An experiment (Fig. S1B) showed that BAC was present in cells in a sufficient amount after 2 hours of cultivation at 37 °C even without the addition of a replication inducer (Fig. S1B, lane 1). Hence, we decided to check whether it is possible to obtain sufficient amounts of BAC by growing a large number of cells overnight without using a replication inducer. To prevent the culture from overgrowing and to slow down the replication rate, cells were grown at room temperature overnight. Cultivation was done in the presence of different amounts of Cm (12.5 and 25 µg/ml; lane 1 in Fig. S1C and D, respectively). We found after overnight cultivation that a far greater BAC amount was maintained in the cells that were grown in the presence of a twice higher Cm concentration (compare lane 1 in Fig. S1C and D). We concluded that an increased Cm concentration allowed the cells to maintain a greater BAC amount in overnight cultures. The pCC1BAC series plasmids contain the Cm resistance marker CAT, which inactivates Cm by acetylation. Hence, Cm is a consumable antibiotic. Thus, a higher starting Cm concentration ensures a longer maintenance of BAC in the culture medium.

The overnight culture was then diluted with the fresh LB medium to 1 OD, and cells were returned to the usual mode of cultivation at 37 °C for 2 hours to increase the cell yield. Cell cultivation was continued at 37 °C either without (Fig. S1C, lane 2) or with an additional portion of Cm (Fig. S1D, lane 2). We observed that BAC was lost very quickly upon cultivation at 37 °C without Cm (Fig. S1C, compare lanes 1 and 2). Cultivation in the presence of Cm made it possible to increase the BAC amount in the culture (Fig. S1D, compare lanes 1 and 2). The effect was unexpected. BAC contains an F-factor origin, which should ensure its stable maintenance in a single-copy state. However, BAC

behaves like a multicopy plasmid in EP300 cells and is lost once Cm is consumed. We concluded that stable maintenance of BAC in the medium of overnight cultures without induction of replication is possible only when a sufficient Cm amount is used at the start of cultivation or additional portions of this consumable antibiotic are added during cultivation.

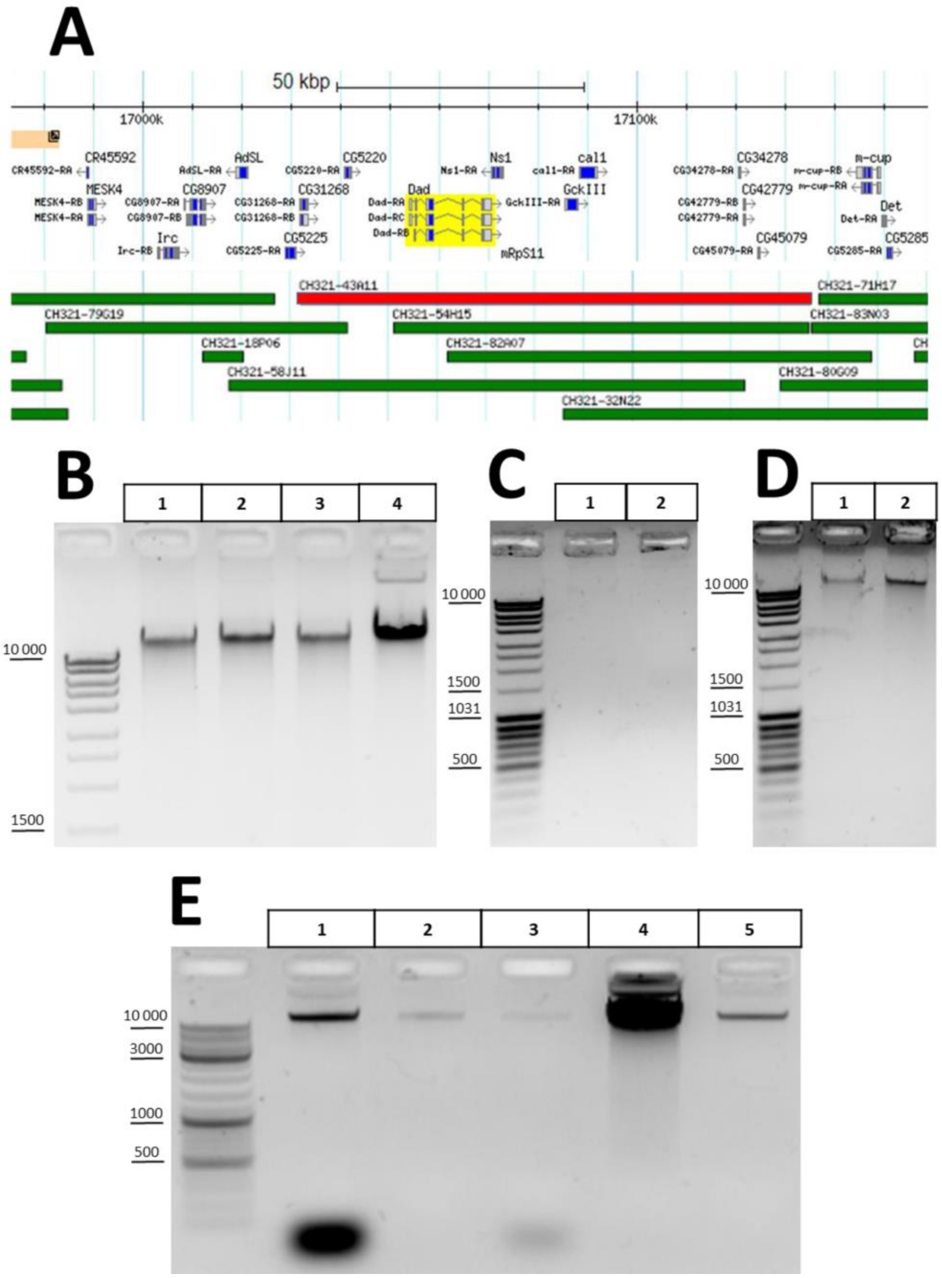

Figure S1. Cultivation and purification of BAC.

(A) The *D. melanogaster* genomic region that includes the model gene *Dad* is presented. The BAC in use is marked red. The *Dad* gene is highlighted yellow.

(B) Cells of the *E. coli* strain EPI300 were grown on 25 µg/ml chloramphenicol (Cm) LB-agar plates overnight and then inoculated into the liquid LB medium and grown to 1 OD in the presence of 25 µg/ml Cm at 37 °C. A cell batch was divided into four replicates, each replicate was diluted by a factor of 10, Cm was added to 25 µg/ml, and replication inductors were added or not. The cells were allowed to grow for 2 hours at 37 °C and harvested, and BAC was isolated from 1 ml of cells. 1, no inductor (control); 2, inactivated tetracycline hydrochloride (conc. 20 µg/ml); 3, inactivated chlortetracycline (conc. 20 µg/ml); 4, L-arabinose (20 mM). Stock solutions of inductors 2 and 3 (10 mg/ml) were autoclaved in mQ water for 30 minutes before the experiment.

(C) Cells of the *E. coli* strain EPI300 were grown in the liquid LB medium in the presence of 12.5 µg/ml Cm at RT overnight. The culture was diluted with a fresh LB medium to 1 OD, and BAC was isolated from 1 ml of cells (lane 1). The rest of the culture was further incubated at 37 °C for 2 hours. Then the culture was diluted again to 1 OD, and BAC was isolated from 1 ml of cells (lane 2).

(D) Cells of the *E. coli* strain EPI300 were grown in the liquid LB medium in the presence of 25 µg/ml Cm at RT overnight (lane 1). The culture was diluted with a fresh LB medium to 1 OD, and BAC was isolated from 1 ml of cells. Cm was added to the rest of the culture to 25 µg/ml, and cultivation was continued at 37 °C for 2 hours (lane 2). Then the culture was diluted again to 1 OD, and BAC was isolated from 1 ml of cells.

(E) Purification of BAC on magnetic AMPure XP beads. BAC isolated with a column kit (lane 1) was precipitated with ethanol, dissolved in 10 mM Tris-HCl at RT, and transferred into new tubes. The old tubes were rinsed with 50 µl of 10 mM Tris-HCl, and 1 µl of the washing was subjected to electrophoresis (lane 2). The pooled BAC was treated with bovine RNase A (1 µl of 10 mg/ml RNase A per 200 µl of BAC) at RT for 30 min and mixed with 1.5 volumes of AMPure XP beads. After separation of the beads with a magnet, material contained in the supernatant was precipitated with ethanol and dissolved in 300 µl of 10 mM Tris-HCl. Elution of BAC from the beads was done with the same volume of 10 mM Tris-HCl at RT for 10 min. Aliquots (1 µl) of the BAC eluate and the supernatant precipitate were resolved in gel (lanes 4 and 3, respectively) to see how much of BAC remained in the supernatant. After the first BAC elution from the beads, the beads were incubated with 50 µl of 10 mM Tris-HCl at an elevated temperature (50 °C) for 5 min to see how much of BAC remained absorbed on the beads (lane 5).

2.2 Isolation of BAC

We concluded from the above experiments that BAC behaves like a multicopy plasmid. Hence, we assumed that standard plasmid preparation procedures might be employed in BAC isolation. Indeed, a GeneJET plasmid miniprep kit (# K0503, Thermo) was successfully used with small culture volumes. Preparative volumes were processed using a GeneJET plasmid midiprep kit (# K0481, Thermo) or Plasmid midiprep 2.0 (#BC124, Evrogen).

2.3 Purification of BAC

DNA preparations isolated with column kits are usually inferior in purity to preparations obtained via phenol/chloroform (Ph/Chl) extraction. Therefore, additional purification of the column-isolated BAC was performed, taking advantage of Beckman Coulter AMPure XP paramagnetic beads (solid phase reversible immobilization technology, SPRI). These beads are routinely used to perform size selection and to purify PCR products and Hi-C libraries (Rao et al., 2014; Belaghzal et al., 2017; Canela et al., 2017). The purification process was studied. We found the following. (i) Upon dissolution of the ethanol-precipitated BAC, all accompanying RNA passed into the solvent, while part of the BAC selectively remained on the walls of the tubes (Fig. S1E, lane 2). (ii) Only BAC was absorbed on AMPure XP beads, while low-molecular-weight RNA was not, and only traces of BAC and low-molecular-weight RNA were present in the supernatant after absorption on beads (Fig. S1E, lanes 3,4). (iii) Incubation of beads with 10 mM Tris-HCl at an elevated temperature (50 °C) did not cause BAC degradation (Fig. S1E, lane 5). We also observed that isolation on AMPure XP beads is accompanied by lower losses of BAC DNA as compared with ethanol precipitation. The difference in qPCR between the BAC DNA isolated with AMPure XP beads and the routinely ethanol-precipitated BAC DNA was 2.7-3.75 Ct.

Thus, the step of additional purification of BAC on magnetic beads does not lead to its degradation and occurs without large losses of BAC DNA.

2.4 Restriction digestion and ligation of BAC to prepare random ligation library

To obtain a high-resolution 3C library, it is appropriate to use a 4-bp cutter RE, which cuts DNA approximately every 256 bp. At least four different REs can be used for the purpose of restriction digestion of BAC and genomic DNA at the GATC site. These REs have different specificities for DNAs of eukaryotes and bacteria (Table S2). For example, DpnI does not cleave fly genomic DNA at all (Fig. S2A) and usually serves to digest adenosine-methylated bacterial DNA. In our experiments, DpnII was used to digest fly genomic DNA in nuclei as the RE that is most commonly used in *Drosophila* studies (Comet et al., 2011; Sexton et al., 2012; Eagen et al., 2015, 2017; Li et al., 2015; Ulianov et al., 2016, 2021; Cubeñas-Potts et al., 2017; El-Sharnouby et al., 2017; Rowley et al., 2017; Chathoth and Zabet, 2019) (Fig. S2A). However, DpnII is unable to digest BAC and plasmid DNAs isolated from bacteria (Fig. S2B). Therefore, to prepare a random ligation library from BAC, we used endonuclease Sau3AI (BspI), which is insensitive to adenosine methylation of bacterial DNA and efficiently digests BAC and plasmid DNAs (Fig. S2C). Moreover, genomic DNA isolated from S2 *Drosophila* embryonic cells was efficiently cleaved with Sau3AI (BspI) as well (Fig. S2C) since methylation of *Drosophila* DNA is extremely rare at all developmental stages (Deshmukh et al., 2018). However, Sau3AI (BspI) preparations are unavailable in a high-concentration form (e.g., 50 U/µl), in contrast to DpnII, and their use to digest DNA in nuclei is therefore not advisable since a substantial amount of an enzyme preparation should be added into the reaction mixture to create a high concentration of the enzyme and may lead to inhibition of the reaction by a high glycerol concentration.

Thus, DpnII was used at high concentrations for nuclear DNA digestion and Sau3AI (BspI), for preparation of a random ligation library.

Preparative restriction digestion of BAC DNA was done to obtain a sufficient amount of a random ligation library preparation for constructing a calibration curve (Fig. S2D). We found that dilution of the ligation reaction mixture (Fig. S2E) and addition of fresh ATP (Fig. S2F) or incubation in the presence of a new portion of T4 DNA ligase and ATP (Fig. S2G) did not further improve DNA ligation and the yield of higher-molecular-weight DNA. After purification on beads and double elution of DNA, traces of DNA were still present on the beads (Fig. S2H).

Thus, 25 μg of BAC DNA can be successfully ligated in conditions described in Fig. S2E after restriction digestion and additional ligation rounds do not improve the overall picture. We recommend additionally that elution be repeated twice when purifying the library of randomly ligated BAC fragments.

Table S2. Specificities of REs recognizing the GATC site

Restriction enzyme	Digestion site	End	Dam methylatio n	CpG methylatio n	Decision
DpnI	GA / TC	Blunt	+	–	Nuclei: no<br>BAC: yes (recognizes only methylated DNA)
DpnII	/ GATC	5’	–	+	Nuclei: yes<br>BAC: no
Sau3AI (BspI)	/ GATC	5’	+	–	Nuclei: no<br>BAC: yes (fly, yes; mammals, no)
MboI	/ GATC	5’	–	+/–	Nuclei: +/- (fly, yes; mammals, no)<br>BAC: no

«+», cleaves; «–», does not cleave

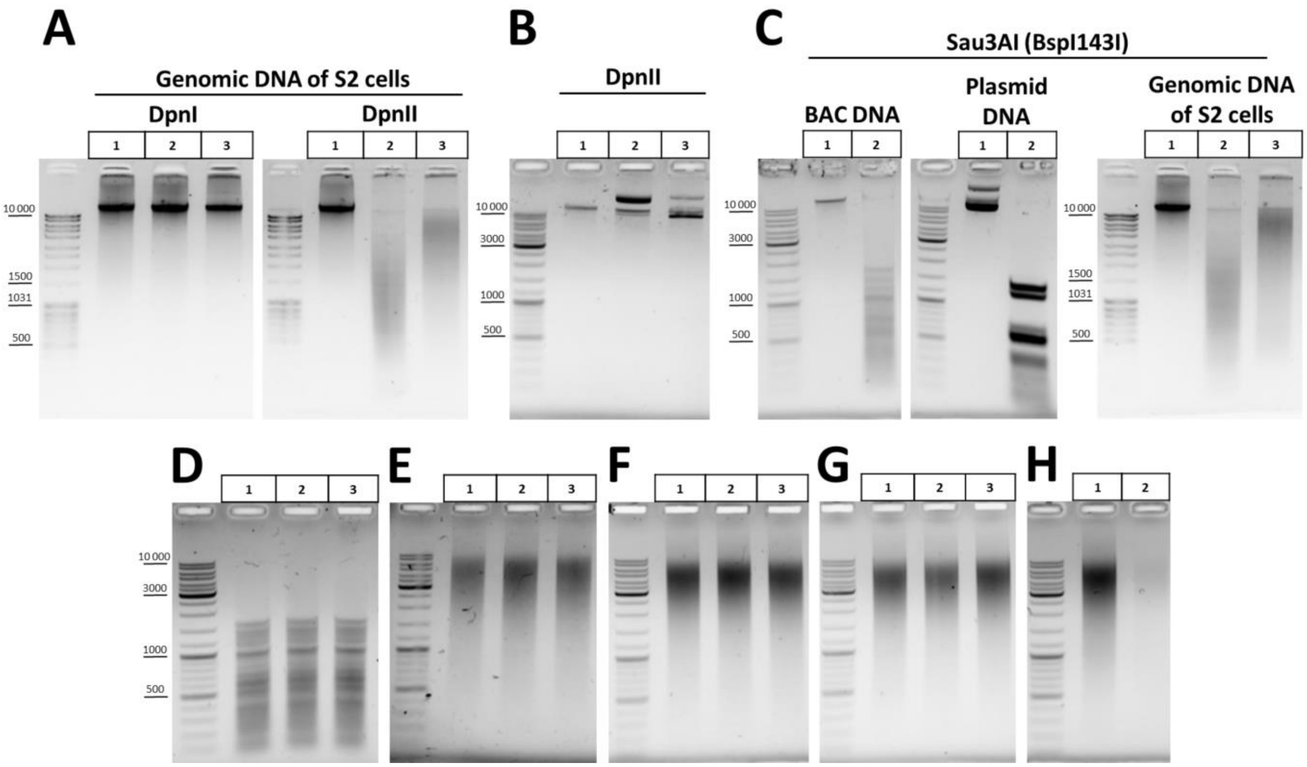

Figure S2. Comparison of GATC RE activity on bacterial and fly DNAs.

(A) Genomic DNA of S2 cells was isolated as follows: S2 cells (100 mg) were harvested, washed with 1X PBS, and resuspended in 1 ml of EB. The cells were incubated at 56 °C for 30 min, and DNA was extracted, precipitated (w/o glycogen), washed as in Fig. 1D, dissolved in 150 µl of Tris-HCl pH 7.9, treated with 50 U of RNase I (Thermo) at RT for 30 min, purified using 1.5X AMPure XP beads, and eluted with 150 µl of 10 mM Tris-HCl (pH 7.9). Genomic DNA was digested with either 10 U of DpnI in the Tango buffer or 10 U of DpnII in the DpnII buffer (pH 6.0). The REs were heat inactivated, and DNA was precipitated with ethanol and ligated. Lane 1, nondigested DNA; lane 2, digested DNA; lane 3, DNA ligated in solution after digestion.

(B) BAC was cultivated as described in Fig. S1D and was isolated as described in the section *Isolation of BAC*. Plasmid DNA was isolated as described in *Materials and methods*. BAC and plasmid DNAs were digested with 10 U of DpnII in the DpnII buffer (pH 6.0) and directly subjected to electrophoresis. Lane 1, nondigested BAC; lane 2, BAC digested with DpnII; lane 3, the plasmid digested with DpnII.

(C) BAC, plasmid, and genomic DNAs (indicated at the top) were digested with 10 U of Sau3AI (BspI) in the Sau3AI buffer. After digestion, Sau3AI was inactivated by heating and DNA was resolved in gel (left and central panels) or precipitated with ethanol and ligated (right panel). Lane 1, nondigested DNA; lane 2, digested DNA; lane 3, DNA ligated in solution after digestion.

(D-E) Preparative restriction digestion and ligation of BAC. (D) Restriction digestion was performed using 25 µg of total DNA of a BAC (104 276 bp) + plasmid (12 617 bp) equimolar mixture (Miele and Dekker, 2009). The restriction reaction was done in a volume of 100 µl in three replicates (75 µg of the BAC + plasmid mixture in total), with 100 U of Sau3AI (BspI) per replicate, overnight. An aliquot (1 µl) of the restriction reaction mixture was resolved in gel. After digestion, DNA was purified using a 3X volume of AMPure XP magnetic beads and eluted with 10 mM Tris-HCl (pH 8.0) at RT for 10 min. (E) DNA ligation was performed using 25 µg of DNA from the BAC + plasmid digestion mixture purified on the beads, in a volume of 50 µl in the presence of 0.4 U/µl T4 DNA ligase (Sibenzyme, 10 U/µl conc.) and 1 mM ATP at 16 °C overnight, in three replicates.

(F) The ligation mixture from (E) was diluted by a factor of 2, fresh ATP was added to 1 mM, and the reaction in three replicates was incubated for more than 1.5 hours at 16 °C.

(G) Fresh T4 DNA ligase (10 U) and fresh ATP (to 1 mM) were added to the ligation mixture from (F), and incubation in three replicates was continued at 16 °C overnight.

(H) The ligation reaction mixtures from (G) were pooled. DNA was purified using a 3X volume of AMPure XP beads. DNA was eluted from the AMPure XP beads with 150 µl of 10 mM Tris-HCl twice: first, at RT for 10 min and second, at 50 °C for 5 min. Two sequential elutions were pooled (lane 1), and 1 µl of pooled BAC was subjected to electrophoresis. The supernatant was saved after separation of the beads with a magnet. Its material was precipitated with ethanol and dissolved in 25 µl; 1 µl was taken for electrophoresis (lane 2).

3. Preparation of the calibration curve based on a random ligation library

After restriction digestion and ligation of BAC, the preparation contains ligation products formed between all possible DpnII fragments of the genomic locus of interest, i.e., it is a random ligation library. The ligation frequency of the target fragments in the mixture is quite high, which makes it possible to detect the signal using qPCR even after multiple dilutions of the library. This favorably distinguishes the preparation of a BAC random ligation library from preparation of a randomly ligated mixture of genomic DNA fragments, where target ligated fragments occur at an extremely low frequency and are practically impossible to detect using PCR. To achieve a higher efficiency of detecting the ligation products in a random ligation library, we optimized the qPCR conditions before doing the main PCR experiments.

3.1 Optimization of the TaqMan real-time PCR conditions

To increase the sensitivity of the assay and to eliminate the possibility of nonspecific amplification and amplification of primer dimers, which is often observed in SYBR green qPCR (Ruiz-Villalba et al., 2017), we chose the TaqMan probe-based detection system. To achieve a maximum efficiency of TaqMan PCR, we optimized the concentrations of primers and TaqMan probes, the PCR mode (three step or two step), and the reaction volume of the TaqMan PCR reaction (Table S3). The optimal

primer concentration was determined to be 0.3 pM/µl; the TaqMan probe concentration was chosen to be 0.3 pM/µl since this provided a good balance between costs and qPCR performance (Fig. S3A). A two-step PCR mode was chosen since it was slightly more efficient than the classical three-step mode. In this mode, the annealing and primer extension are carried out at the same temperature. These conditions provided an increase in the efficiency of TaqMan PCR by an average of 2.5 Ct (Table S3). A halving of the reaction volume, to 5 µL, increased the qPCR efficiency up to 4.5 Ct; however, reactions in such a volume were not always successful in a 96-well plate, and we therefore chose a reaction volume of 10 µL. A representative experiment is shown in Fig. S3B.

Table S3. Comparison of the TaqMan real-time PCR conditions and gain in PCR Ct

Parameter	Basic conditions	Maximum efficiency	Maximum reliability (reproducibility)
Probe concentration, pM	0.16 pM / µl	0.5-1 pM / µl	0.3 pM / µl
Primers concentration, pM	0.4 pM / µl	0.1-0.2 pM / µl	0.3 pM / µl
Reaction volume, µl	20 µl	5 µl	10 µl
Number of PCR steps	3 (94° 10’’, 60° 25’’, 72° 35’’)	2 (94° 10’’, 60° 1’)	2 (94° 10’’, 60° 1’)
Gain in PCR, Ct	0	4.5	2.5

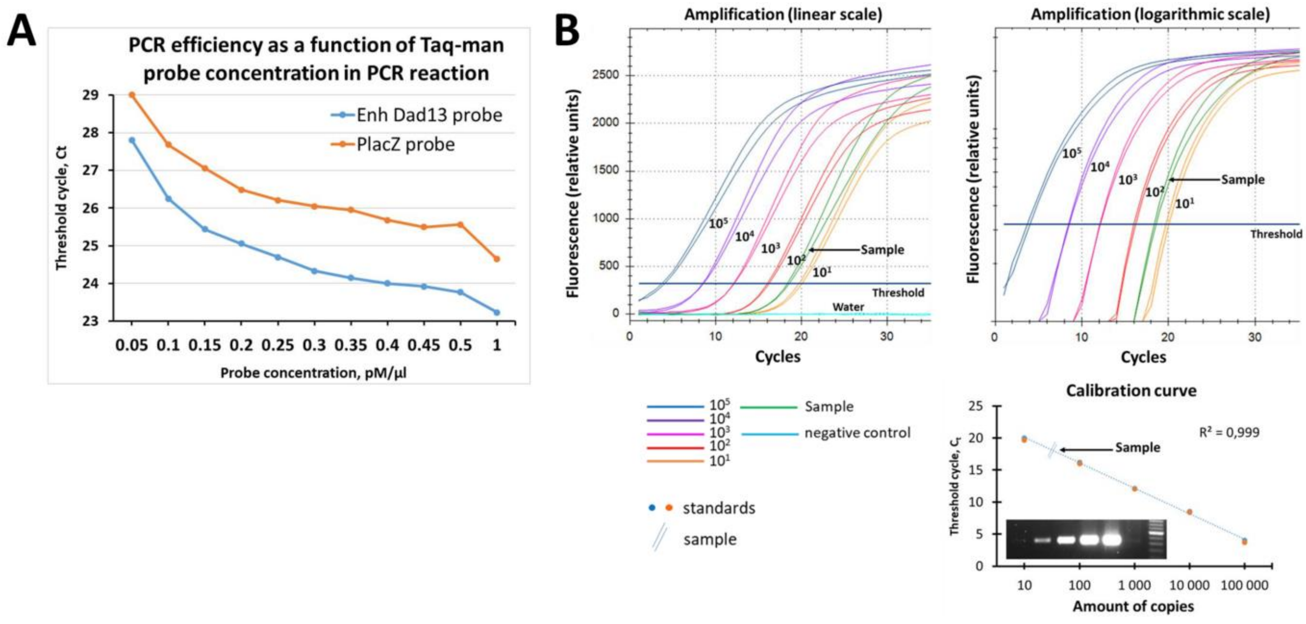

Figure S4. Titration of the TaqMan probe and qPCR experiment arrangement with TaqMan probes. (A) Ct as a function of Taq-man probe concentration in PCR. The relationship was studied for two probes using 10 ng of *Drosophila* genomic DNA as a template. The source fly strain carried the reporter cassette LexAop-hsp70-lacZ^{Dad} inserted in the endogenous *Dad* promoter region about 5 kb upstream of the mapped *Dad13* enhancer (Shidlovskii et al., 2021). The probe at *Dad13* enhancer (probe 5 in Table S1) with the anchor primer at *Dad13* enhancer (primer #5 in Table S1) and the *Dad13* enhancer test primer (5_test in Table S1) and the probe at $P_{lexAop\text{-}hsp70}$-lacZ promoter (probe 9 in Table S1) with the anchor primer at $P_{lexAop\text{-}hsp70}$-lacZ promoter (primer #9 in Table S1) and the $P_{lexAop\text{-}hsp70}$-lacZ promoter test primer (9_test in Table S1) were used, respectively, to estimate the efficiency of amplification.

(B) An example of the qPCR experiment arrangement with TaqMan probes. Fluorescence growth curves (linear and logarithmic scales) are presented at the top. Green curves show the amplification results for the sample; blue curves, for the negative control (water or genomic DNA digested with DpnII and randomly ligated in solution); and curves of different colors, for the DNA standards. A standard curve and an electrophoretic picture of the amplified standards are presented at the bottom right.

3.2 Determination of ligation frequencies in a random ligation library

To determine that the ligation products of different sites are equally represented in the random ligation library, we chose the developmental gene *Dad* as a model locus, in which a reporter P-element transgene containing the $P_{lexAop\text{-}hsp70}$-lacZ expression cassette was integrated upstream of the *Dad* promoter region (Fig. S4A) (Shidlovskii et al., 2021). We estimated the ligation frequency of a chosen DpnII fragment (#7) with an anchor fragment in relation to the other fragments of interest, taking the ligation frequency of fragment #7 with the anchor as 100%.

We found that the ligation frequency with the anchor DpnII fragment (#5) did not differ significantly between different DpnII fragments in our random ligation library and was within the statistical error (Fig. S4B). The experiment was repeated with another anchor (#9), and comparable relative ligation frequencies were observed with different anchors (Fig. S4B). This shows that the overall picture is reproduced when "viewed" from different points.

The between-fragment differences in ligation frequency, which were found by PCR, may be a consequence of (i) fluctuations in the number of ligation events during ligation, (ii) a slight discrepancy in the actual amount of plasmid and BAC DNAs after their equimolar mixing, or (iii) differences in PCR efficiency between different combinations of the anchor primer and the test primer (difference in PCR efficiency between different experimental points). In turn, different amplification kinetics observed for different amplicons can be a consequence of their different lengths and temperatures of primer annealing. These reflections suggest that determination of the ligation frequency in an experimental sample with a certain pair of primers should be carried out in parallel with a calibration curve, which is to be tested with the same pair of primers. This makes it possible to allow and correct for the difference in PCR efficiency between different experimental points (primer pairs).

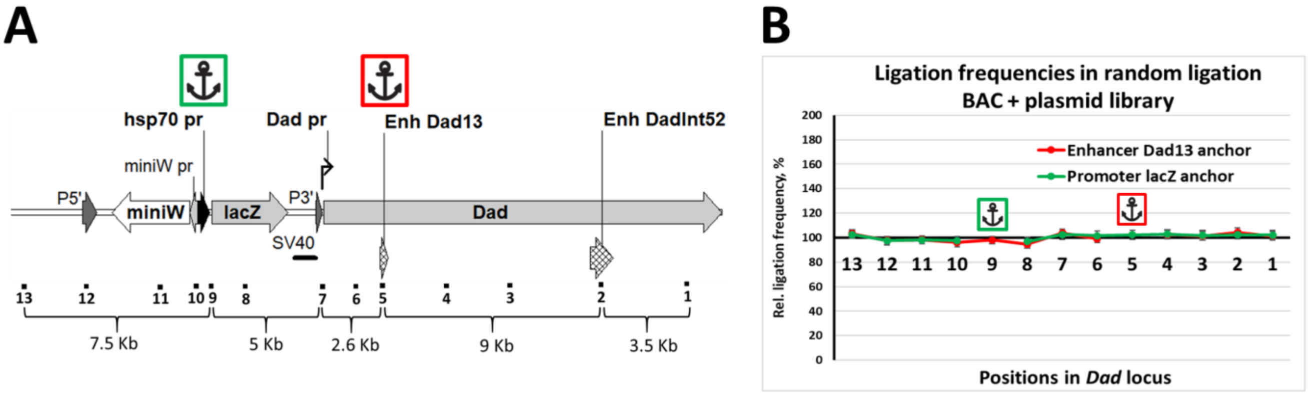

Figure S4. Determination of ligation frequencies in a random ligation library.

(A) The model locus *Dad* is shown with the adjacent P-element reporter transgene and the positions of two anchors, one on the enhancer *Dad13* and the other on the $P_{lexAop\text{-}hsp70}$-lacZ promoter. The numbers of experimental points and distances between the main points are indicated with digits below the picture. (B) The relative ligation frequencies with an anchor (point #5 or #9) were determined for different experimental points in relation to the frequency of ligation of the anchor with fragment #7 (*Dad* endogenous promoter), which was arbitrarily taken as 100%. Error bars indicate SDs of 4 PCR technical measurements for each experimental point.

3.3 Determination of linear range of amplification of random ligation library

To ensure that dilutions of the random ligation library amplify linearly, we prepared dilutions of the library and tested them with several primer pairs. For this purpose, we chose 4 primers from the list of all primers presented in Table S1. In combination with anchor primer #5, these ones give the greatest variation in the GC composition and amplicon length (Table S4). Using these pairs of primers, the efficiency of amplification of the target PCR products was evaluated using 10X dilutions of the random ligation library.

We found that the ligation products of BAC fragments (#5-7) as well as BAC and plasmid fragments (#5-9, 5-10, 5-12) were easily detectable in the random library. The number of ligation products was sufficient for making several 10X dilutions starting from 10 ng/µl (Fig. S5). Dilutions from 10 ng/µl to 100 fg/µl provided linear amplification. Under our PCR conditions, the dilutions in the linear part of the calibration curve differed by 3.5 Ct. Dilutions below 100 fg/µl no longer allowed linear detection of the product and often gave a large scatter in replicates; i.e., they were beyond the sensitivity of the method in our conditions (Fig. S5).

We concluded that a calibration curve based on dilutions from 10 ng/µl to 1 pg/µl can be routinely used to determine the cross-linking frequency in an experimental 3C library. The point of 100 fg/µl can also be used to obtain additional depth of the calibration curve.

Table S4. Lengths and GC compositions of amplicons for determination of the dilutions in a calibration curve

Point number	Primer pair	Amplicon length, bp	GC composition, %
1	5-1	112	57.1
2	5-2	296	52.4
3	5-3	152	55.3
4	5-4	136	58.1
6	5-6	118	54.7
**7***	5-7	333	45.6
8	5-8	232	53.4
**9**	5-9	280	43.2
**10**	5-10	183	50.8
11	5-11	219	52.1

**12**	5-12	189	59.3
13	5-13	290	46.9

* - The primer pairs analyzed are in bold.

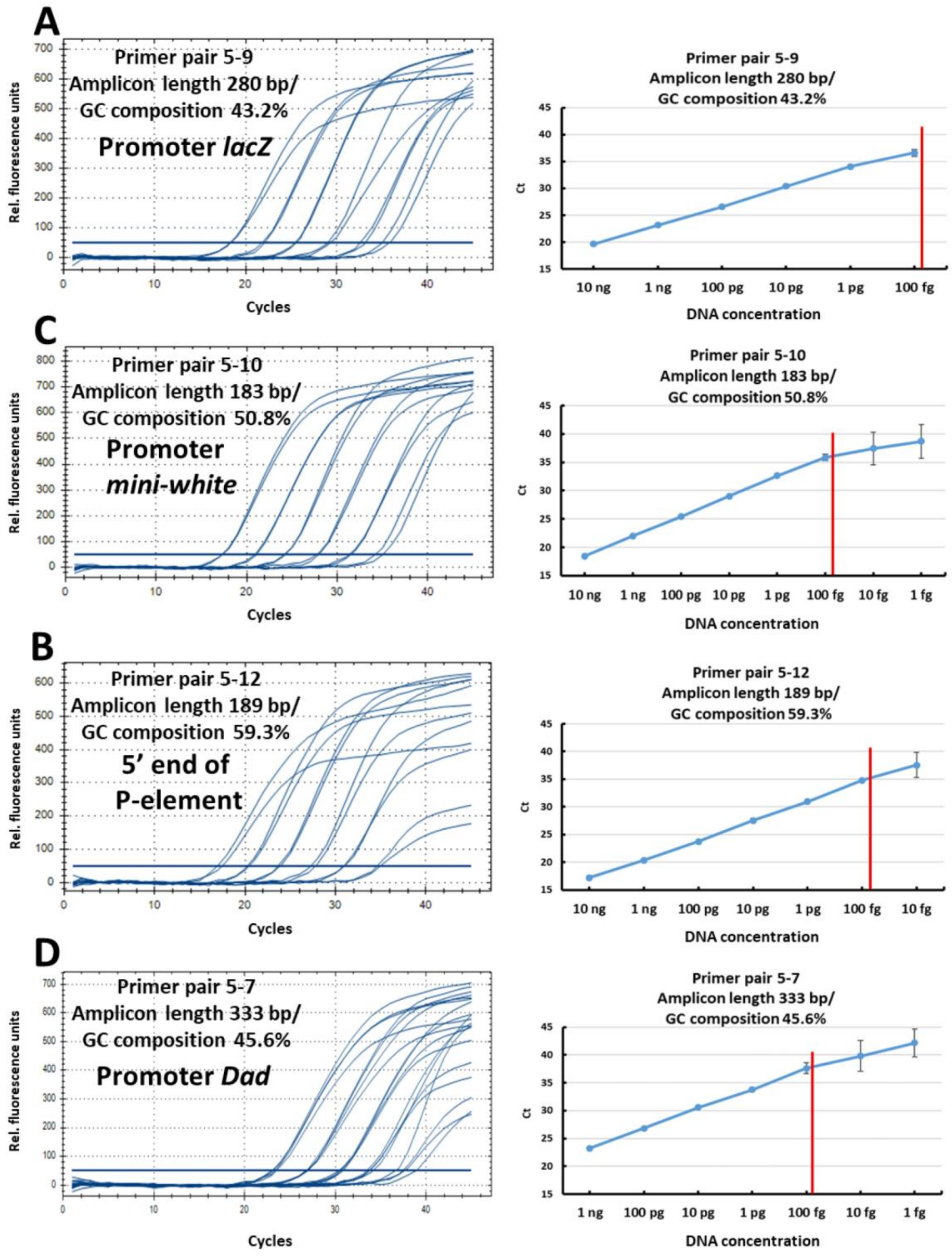

Figure S5. Calibration curves obtained with 10X dilutions of the random ligation library for four primer pairs with different GC compositions and amplicon lengths.

(A-D) Amplification curves of qPCR are shown for each primer pair on the left. On the right, PCR efficiency (Ct) as a function of library dilution is shown for each primer pair. (A) Primer pair 5-9, which anneals to the *lacZ* promoter; (B) primer pair 5-12, which anneals to the 5’ end of the P-element; (C) primer pair 5-10, which anneals to the *mini-white* promoter; (D) primer pair 5-7, which

anneals to the *Dad* promoter. A red line designates the last dilution in which linearity of amplification is still observed, as well as a low scattering of values. Error bars indicate SDs of four PCR technical measurements for each experimental point.

3.4 Establishment of the amount of 3C DNA libraries required to fit the calibration curve

Further, we checked between what dilutions of the calibration curve the amplification products of the experimental 3C library could be detected. We also studied how the dilution of the experimental 3C library or, conversely, an increase of its amount in PCR could affect the time of appearance of the amplification product. For this experiment, we used the pair of primers (#5-7), which detects the interaction of the enhancer *Dad13* with the *Dad* promoter. 3C libraries were prepared from 10 mg of S2 cells (~12 million cells) in 12 replicates. The concentrations of the libraries were measured using a Qubit 2.0 and dsDNA HS Assay kit, #Q33230. The concentrations varied from 62.3 ng/µl to 85.1 ng/µl. The libraries were pre-aligned for DNA concentration and diluted by a factor of 10 or 100 times or left undiluted and subjected to qPCR (62.3 ng/µl–6.23 ng/µl–0.623 ng/µl).

The analysis showed a linear dependence of the Ct values of experimental 3C samples on their concentration (Fig. S6A,B). We found that at least a tenfold dilution was possible for the experimental 3C library for the results of amplification to fall between the points 1 pg and 10 pg of the calibration curve. Dilution of the library by a factor of 100 led to a drop in the qPCR signal below the last calibration point of 100 fg. Thus, a 100-fold dilution is not recommended since reliable interpretation of the results is impossible when a sample Ct is below the last calibration point. We conclude that, if the procedure described in Figure 2D-H is used to prepare the random ligation library, the concentrations of experimental libraries from S2 cells that can be reliably measured range from 6.23 to 62.3 ng. Our results are in good agreement with the results by (Gavrilov and Razin, 2008), who showed that 30-60 ng of a 3C template prepared from chicken cells is enough to obtain a strong PCR signal.

We also found that amplification products of experimental 3C libraries are detected approximately simultaneously when equalization of concentrations was performed (Fig. S6C,D). Nevertheless, this result suggests that an internal control is required to normalize the absolute DNA amounts in experimental 3C samples and to correct for differences in ligation efficiency between them. The same control can be used to normalize the ligation efficiency between different cell lines or experimental materials of different sources (Gavrilov and Razin, 2008). Such a normalization of the data has no effect on the profile of the 3C curves, although slightly changing the absolute values (Gavrilov and Razin, 2008). If interactions are measured in different technical replicates within the same tissue or cell line (biological replicate, biological sample) and the entire 3C procedure is carried out the same way for these technical replicates, then the amount of the 3C amplicon product can be normalized relative to an amplicon that does not span a DpnII site and gives a measure of the total DNA in the reaction (Bieli et al., 2015). Comet et al. proposed to use *RpII* locus for normalization in *Drosophila* (Comet et al., 2011). The primers and probes that we designed for normalization to *RpII* and the equation used to calculate the ligation yield in *RpII* are shown in Fig. S6E,F and Table S1 (for

details, see caption to Fig. S6E). The primer pair designated as REF can be used for normalization within technical replicates of one biological sample.

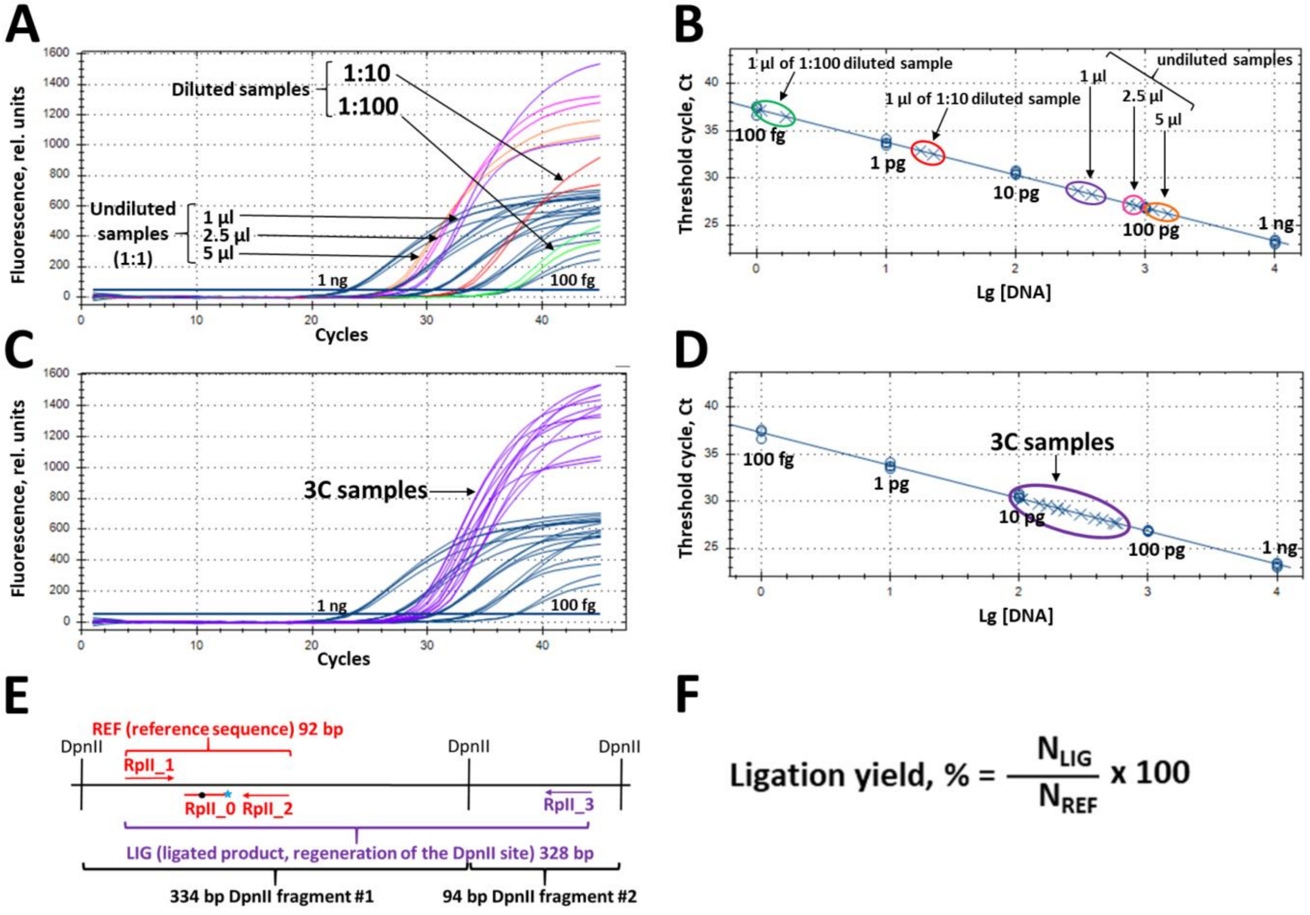

Figure S6. Testing of dilutions of experimental 3C libraries prepared from S2 cells with the calibration curve made from a BAC random ligation library.

S2 cells (10 mg, ~12 x 10^6 cells) were fixed with 0.5% PFA in 1X PBS at RT for 10 min, quenched with equimolar glycine at RT for 5 min, and washed twice with ice-cold 1X PBS. Then the cells were lysed. Nuclei were washed, heated in the presence of SDS/Triton X-100, washed with 1X RB as in Fig. 1D, resuspended in 1X RB, and incubated in the presence of 2 U/µl DpnII at 37 °C overnight with agitation. After restriction digestion, the nuclei were centrifuged; the supernatant was removed; and the nuclei were washed three times with 1X T4 DNA ligase buffer, resuspended in 1X T4 DNA ligase buffer, and incubated in the presence of 0.25 U/µl T4 DNA ligase (SibEnzyme) at 16 °C overnight with agitation. To control DNA quality, aliquots (1/10 of the sample volume) were taken after chromatin treatment with SDS/Triton X-100 followed by washing of nuclei with 1X RB (undigested chromatin, chromatin integrity control before RE digestion, control #1) and after the restriction reaction followed by washing with 1X T4 DNA ligase buffer (digested chromatin, chromatin restriction control after RE digestion, control #2). In addition, one more separate tube was

included into the procedure (control #3, DNA integrity control throughout all stages of the procedure). This sample was subjected to all treatments and incubations together with the experimental samples except that reconstructed RE and T4 DNA ligase storage buffers were added to this sample during the procedure instead of true RE and T4 DNA ligase. The sample was analyzed at the end simultaneously with the experimental samples to ensure that DNA integrity was maintained throughout all stages of the procedure until the very end. The volumes of the control and experimental samples were adjusted with 1X T4 DNA ligase buffer to 250 µl, and Proteinase K (PrK), SDS, and EDTA were added to 0.2 mg/ml, 1%, and 30 mM, respectively. Cross-links were reversed, and DNA was extracted, precipitated, and washed as in Fig. 1D. Then DNA was dissolved in 20 (for ligation) and 25 (for controls #1, 2, and 3) µl of 10 mM Tris-HCl pH 8.0. Dissolved DNA was treated with 1 U/µl RNase I at RT for 30 min and purified using 1.5 volumes of AMPure XP paramagnetic beads (SPRI). Before elution, the pellet of beads was washed three times with 1 ml of 75% ethanol with vortexing. DNA was eluted with 10 mM Tris-HCl (pH 8.0) at RT for 5 min, using the initial volumes of 25 µl for controls #1, 2, and 3 and 20 µl for ligation. The DNA eluate (1 µl) was resolved in gel in the case of the experimental samples and control #3, or 10 µl of the DNA eluate was taken for electrophoresis in the case of controls #1 and #2. The final 3C libraries were adjusted to 83 µl in volume, equalized for concentration (62.3 ng/µl), and introduced into PCR mixtures in volumes indicated in panels A,B (62.3, 155.75, and 311.5 ng DNA per PCR mixture, respectively) or diluted by a factor of 10 or 100 and taken in PCR in a volume of 1 µl per mixture. Amplification was carried out with primers #5-7 (Table S4).

(A) The qPCR amplification curves were obtained for different volumes of the undiluted experimental 3C library (62.3 ng/µl) and its 10- and 100-fold dilutions and analyzed against the calibration curve made from 10X dilutions of a random ligation library. Dilutions and different volumes of the experimental 3C library are indicated by different colors and arrows. Calibration curves are blue.

(B) The same as in (A), but the samples are shown on a logarithmic calibration curve.

(C) The qPCR amplification curves for all undiluted experimental 3C samples equalized for concentration (62.3 ng/µl). A 1-µl aliquot of a sample was subjected to amplification. Six individual 3C libraries were analyzed.

(D) The same as in (C), but the samples are shown on a logarithmic calibration curve.

(E) Experimental design of the primers and probes directed to the *RpII* locus and used to normalize the samples for ligation efficiency (regeneration of the DpnII site) and to estimate the absolute DNA quantities in experimental 3C samples. The frequency of the intact (uncut and religated) DpnII site can be estimated before and after ligation by using PCR-stop analysis with a combination of the RpII_1, RpII_2, and RpII_3 primers and the RpII_0 probe against the calibration curve constructed with a PCR product obtained with the RpII_1 and RpII_3 primers. The frequency of the regenerated (ligated) original DpnII site is calculated as the difference between the percentage of the intact

(uncut) restriction site in the samples before and after ligation and is termed the ligation yield. To estimate the digestion efficiency, one-third of the sample after overnight digestion was recommended to take when setting up the 3C (Louwers et al., 2009). In our experience, up to half of the digested sample is required to reliably and conveniently measure the digestion efficiency when the 3C procedure was started from 10 mg of S2 cells .

(F) Equation used to calculate the yield of ligation products. N_{LIG} is the copy number of the ligated product; N_{REF} is the copy number of each of the two restriction fragments.

4 Analysis of the 3C libraryPreparation of the PCR master mix and the general arrangement of a qPCR experiment

Preparing a single uniform PCR master mix (MM) for the detection of interactions at all experimental points under study is an important step of the 3C procedure (3). The step follows the selection of dilutions of 3C samples (1) and equalization of their concentration (2) and precedes the normalization of values of different 3C samples (4). The calibration curve for one pair of primers (anchor primer + test primer) constitutes one experimental point and consists of 4–5 10X dilutions of the BAC random ligation library (BAC1–5) (i.e., 1 ng/µl, 100 pg/µl, 10 pg/µl, 1 pg/µl, and 100 fg/µl), usually in 3–4 technical replicates for each dilution. Experimental 3C samples are taken in at least 2–4 technical replicates depending on the experimental material availability (the more, the better). In our case, each experimental sample is a 3C library obtained from cell material prepared from larvae of a particular genotype.

A typical 3C experiment with one probe is shown in Fig. S7. The experiment includes 7 experimental points (7 regions of potential interactions in genomic DNA) and point #8, which serves as a reserve in case of repeating one PCR run. Each experimental point is arranged in a row from left to right (a green rectangle), and experimental points 1–8 (rows) follow one another from top to bottom (green numbers). Each experimental point includes 4 BAC random ligation library dilutions (BAC templates, a yellow rectangle); 6 individual 3C samples (3C templates, a blue rectangle); and a primer mix (PM), which contains a probe, an anchor primer, and a target test primer (a pink rectangle).

To set up the reactions, an enzyme mix (EM) containing DNA-pol, a PCR buffer, dNTPs, and water in required proportions was freshly prepared for all + 1 (reserve) of the yellow and blue templates of one experimental point (one row). According to our observations, an EM containing DNA-pol could be prepared at once for all of the experimental yellow and blue templates in an experiment with one probe (all rows in Fig. S7 + 0.5 rows as a reserve). This EM was stored on ice without losing its activity for at least a week. The EM was added to the PM in the last pink tube, and then the complete reconstructed MM was distributed to the tubes of one experimental point in each green row.

The PM was prepared as follows. First, a pre-primer mix (prePM) was made, consisting of a probe and an anchor primer in a volume that corresponded to the number of yellow and blue templates of

one green row + 1 (reserve) multiplied by the number of green rows + 0.5 rows (reserve). After distributing the prePM into the pink tubes, different test primers are individually added to each pink tube according to the experimental point. A test primer is not added to the last tube with prePM (pink tube #8 in Fig. S7). This tube serves as a reserve for repeating one PCR run in case of an unsuccessful run with some of test primers.

The volumes of the BAC templates and experimental 3C templates were prepared according to the number of expected technical replicates. The DNA amount in a BAC random ligation library usually does not limit the number of technical replicates. However, the DNA amount is limiting in the case of experimental 3C samples, and, therefore, a dilution of the original experimental 3C preparation and a number of appropriate technical replicates should be carefully selected. After an appropriate dilution, the amount of the available DNA template should be sufficient to measure all experimental points with the required number of technical replicates (+ reserve). The number of experimental points and the corresponding number of technical replicates need to be increased proportionally to the number of anchor primers (+ reserve) if potential interactions are to be determined for more than one genomic site. However, experimental 3C templates should not be diluted too much: they might not fall into the calibration curve otherwise.

In the case of the experiment presented in Fig. S7, one yellow tube of the BAC calibration curve or one blue tube of the experimental 3C sample contains 21 μl of the DNA template (the template amount for 4.2 technical replicates provided that the PCR mixture volume is 10 μl). However, the DNA amount and ligation efficiency may differ among different biological models (e.g., live larvae or cultured cell lines). Therefore, in order to estimate the real amount of the ligation product in experimental samples and to understand the number of technical replicates that can be done, we recommend running a preliminary PCR experiment using one experimental 3C sample (the most abundant in DNA) and a pair of primers. If the real amount of ligated DNA will be low, then the number of technical replicates for experimental 3C samples can be reduced to 3 or even 2 (2.1. technical replicates). This gives a more concentrated template DNA. However, if the amount of ligated products is enough, the volumes of experimental 3C templates are prepared in the same way as for BAC templates (21 μl, 4.2 technical replicates).

BAC templates, experimental 3C templates, and prepared PMs are stored at −20 °C. The EM, freshly prepared or kept on ice, is added first to the PM, and then the reconstructed MM is added to the thawed BAC and 3C templates. In the case of the experiment presented in Fig. S7, 21 μl of the MM was combined with 21 μl of the template, giving the volume of 42 μl (4.2 technical replicates). This volume was distributed over 4 wells of a PCR plate to produce 4 technical replicates for each experimental point.

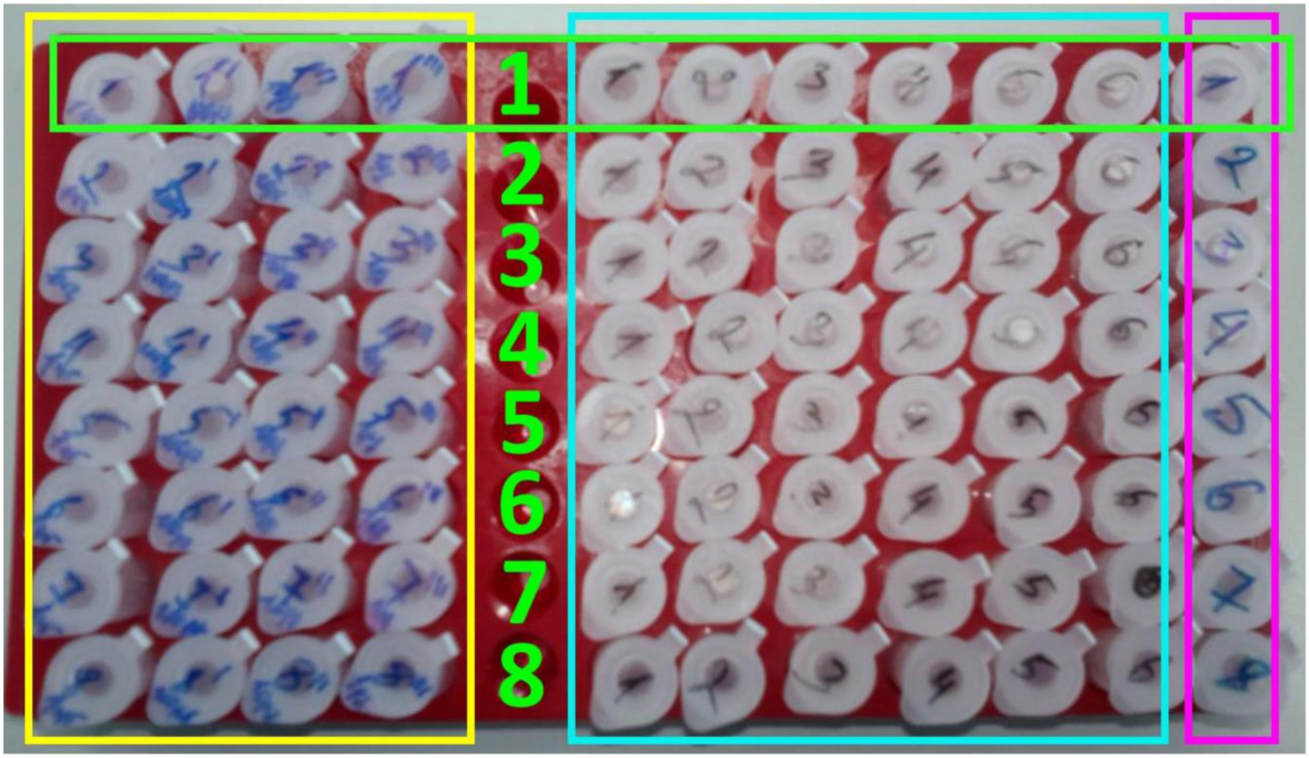

Figure S7. A typical arrangement of a 3C qPCR experiment with one anchor primer and 7 (+1) experimental points (regions of potential interactions in genomic DNA).

Green rectangle, one experimental point, which consists of four yellow BAC templates (four 10X dilutions of a random ligation library to prepare a calibration curve), six blue 3C experimental templates (six individual biological experimental replicates), and one pink tube with PCR master mix (consists of a primer mix + an enzyme mix). Green figures, experimental points (rows) from 1 to 7 according to the number of test primers (row #8 serves as a reserve in case of repeating a PCR run). Yellow rectangle, a set of BAC templates. Blue rectangle, a set of experimental 3C templates. Pink rectangle, a set of PCR master mix tubes for all experimental points from 1 to 7 (+1).

4.2 Calculation of the 3C experiment results

The number of ligated fragments in experimental samples can be estimated using either MS Excel, based the Ct values of the calibration curve, or the algorithm built into the software (we used Bio-Rad CFX manager software for CFX 96). The values of the experimental samples are determined based on the calibration curve in both cases by solving the exponential function equation.

The values of the experimental samples can also be calculated using the TREND function of MS Excel as follows. For the Ct value of an experimental point (e.g., 19), Ct values of the two calibration curve points that bound the interval where the experimental Ct falls (e.g., 17 and 21) are taken as the known *Y* values in MS Excel (e.g., 19). The values of the relative copy number assigned to the two

calibration points (e.g., 100 and 10) are taken as the known *X* values. The Ct of the experimental point should be taken as a new *x* value. As a result, this function will calculate the copy number of the target ligated fragment (e.g., 20.6) for the given experimental point on the basis of the two surrounding points of the calibration curve.

To validate our procedure, we first measured the ligation frequencies for the developmental locus *Dad* in S2 cells (Fig. S8A and S7). Interactions of *Dad13* enhancer were studied (point #5 in Fig. 3A). Experimental 3C samples were amplified simultaneously with dilutions of the calibration curve. The averaged results are presented in Fig. S8B. The results were calculated by the above two methods, using either exponential function equation or the TREND function in MS Excel. When analyzing the results, we noticed that the values calculated using the exponential function equation slightly differed from the values calculated using the TREND function, but the overall picture was essentially the same (Fig. S8B). Six independent biological replicates gave essentially the same 3C profile in both the case of using the exponential function equation (Fig. S8C) and that of using the TREND function (Fig. S8D).

The ligation frequencies of the *Dad13* enhancer region did not significantly vary along the gene in S2 cells but were significantly lower upstream and downstream of the gene (Fig. S8A and S8B). We hypothesized that the *Dad* gene may have a compact conformation in S2 cells, where the gene is inactive.

Since setting a calibration curve every time is time and reagent consuming, we tried to use other approaches to calculate the quantities of the ligation products in experimental 3C samples. The first approach that we used was an adapted principle of the $2^{-\Delta\Delta Ct}$ method (Livak and Schmittgen, 2001; Schmittgen and Livak, 2008; Rao et al., 2013). As a second approach, we tried to normalize the Ct values of 3C samples to the ratios between the anchor primer and different test primers. The ratios were obtained after amplifying the BAC library in a way similar to that in Fig. S3B. Neither approach gave a picture similar to that obtained using the calibration curve (Fig. S9A,B). We concluded that preparation of a calibration curve simultaneously with the analysis of 3C samples is the only option to obtain a reproducible result.

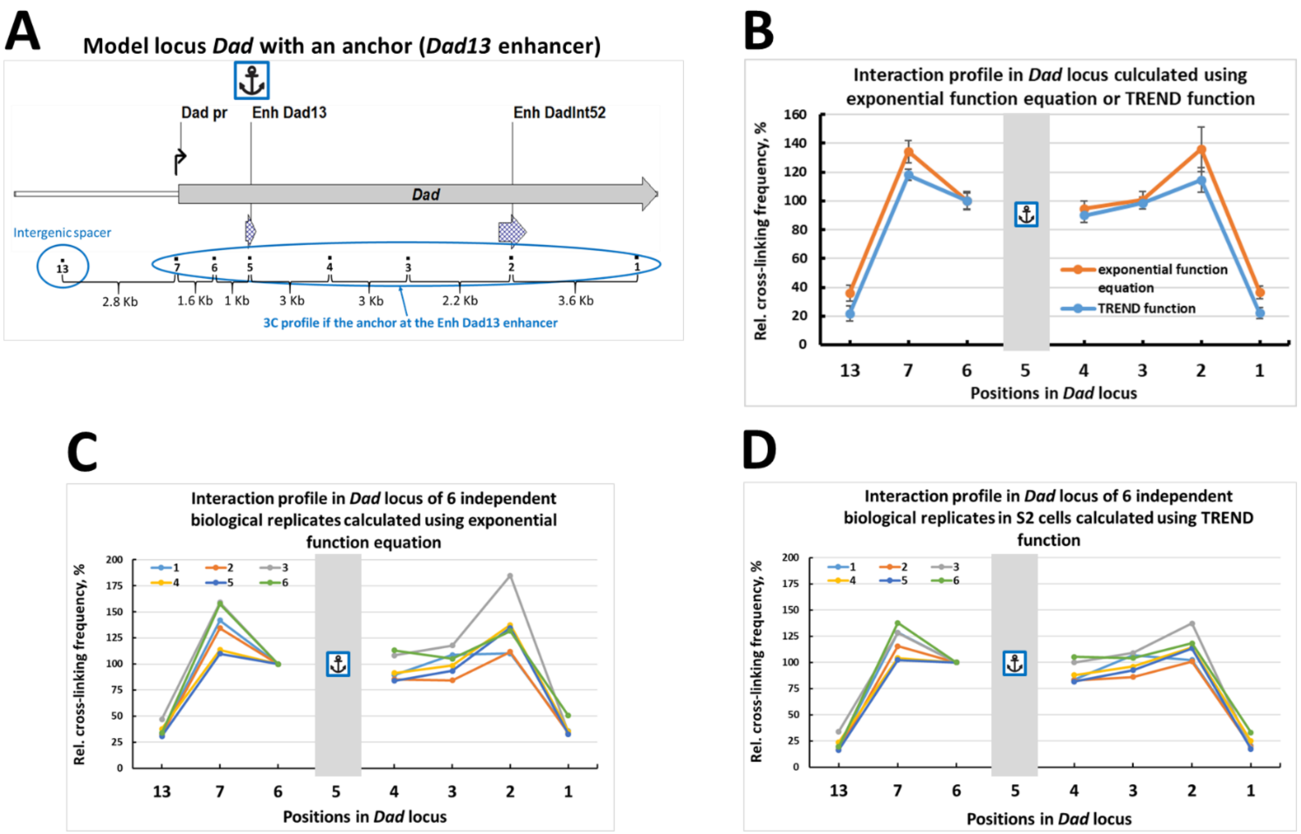

Figure S8. Calculation of the 3C results using the simultaneously measured calibration curve and alternative approaches.

(A) The model wild-type locus *Dad* is shown. The numbers of experimental points and distances between the points are indicated below the scheme. Point #13 is in an intergenic spacer upstream of the gene. The blue oval and circle represent the points under study. The anchor icon indicates the location of the anchor primer. (B) Interaction profile in *Dad* locus as calculated using the BAC calibration curve measured simultaneously. 3C libraries and controls were prepared, treated with RNase, and purified on AMPure XP beads as in Fig. S6. The results were calculated against 10X dilutions of a BAC random ligation library using the exponential function equation or the TREND function in MS Excel. The Y-axis values correspond to the frequencies of cross-linking between the test fragments with normalization to the frequencies of cross-linking between fragments of the control *RpII* locus. Six independent 3C libraries were analyzed. Error bars indicate the SDs of four PCR measurements from six independent biological replicates of the 3C library. (C, D) Interaction profile of the *Dad* locus was calculated using six independent biological replicates with the exponential function equation (C) or TREND function (D). Panel (B) shows the averaged result of the two calculations.

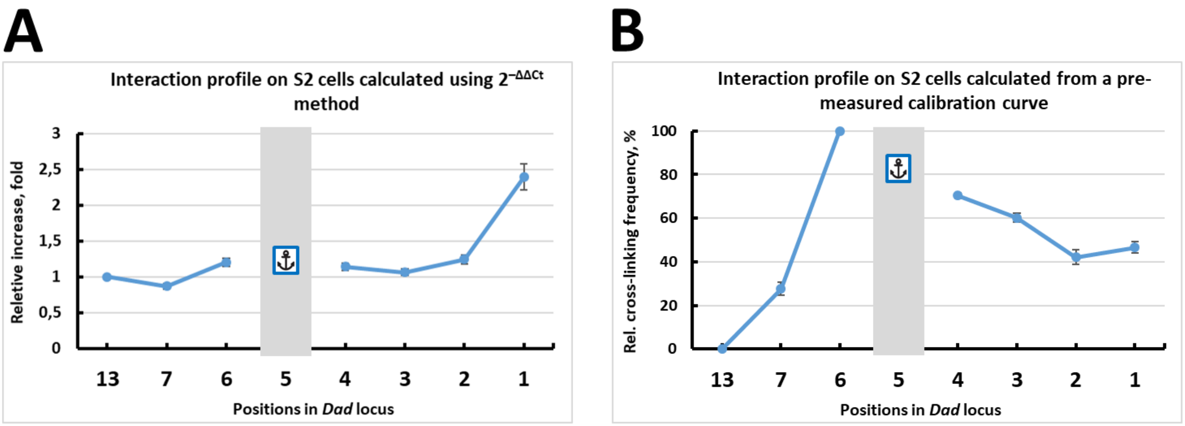

Figure S9. 3C profiles obtained using alternative calculation methods.

(A) Interaction profile of experimental 3C samples as calculated using the $2^{-\Delta\Delta Ct}$ method (Livak and Schmittgen, 2001; Schmittgen and Livak, 2008). See supplementary text for details. (B) Interaction profile of experimental 3C samples as calculated using a pre-determined ratios between fragments in a random ligation BAC library. See supplementary text for details.

4.3 Statistical analysis of the 3C results

We used the arithmetic mean to calculate the average Ct value. In the case of outliers or a large scatter of values, the arithmetic mean can be replaced with a geometric mean, which can be calculated using GEOMEAN function in MS Excel (Smothers et al., 1999; Manikandan, 2011; Martinez and Bartholomew, 2017).

To assess the variation of the mean between independent biological replicates, it is more relevant to use the Standard Error of the Mean (SEM) rather than the Standard Deviation. The SEM reflects the certainty with which the average can be estimated, incorporates the Standard Deviation (SD), and takes into account the number of measurements (biological replicates). A large SEM indicates that the value of a given data point is very uncertain, and it may be necessary to perform additional biological replicates to increase the precision of estimating the Mean (Naumova et al., 2012). The SEM of three biological replicates should not be higher than 15%; additional replicates are otherwise necessary to reduce the error (Miele et al., 2006).

To examine the variation of the Mean between technical replicates of an individual biological replicate, it is relevant to use the Standard Deviation (SD) since the SD better reflects the potentially relevant variation between technical replicates (Naumova et al., 2012).

To make pairwise comparisons of the Mean relative ligation frequency between individual fly lines and between differentially treated cell lines, one-tailed Student’s t-test can be routinely used and the P-value based on the *t*-criterion can be determined.

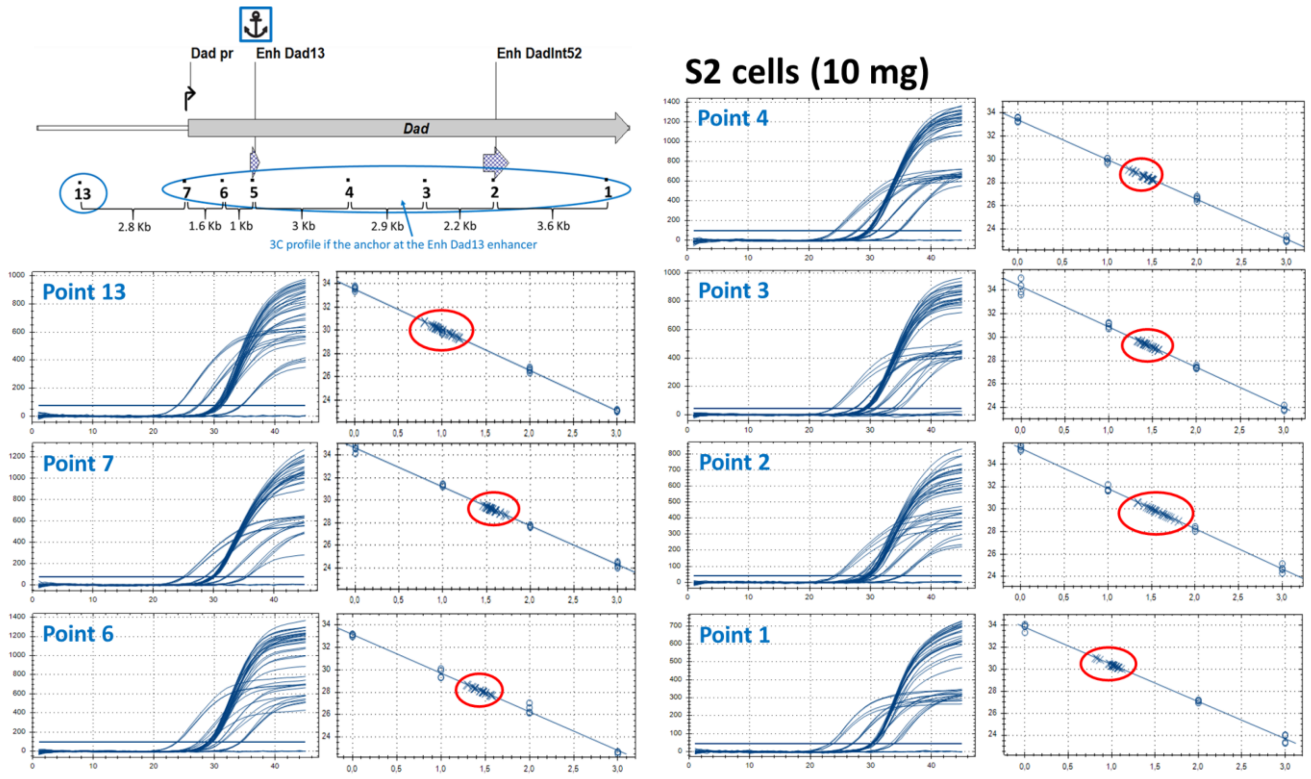

Figure S10. Distribution of experimental 3C samples over the calibration curve in an experiment with S2 cells.

The model while-type locus *Dad* is presented. Positions of 3C samples on the logarithmic calibration curve are shown with red circles. Designations of the X and Y axes are as in Fig. S5. Other designations are as in Fig. S6. Six independent 3C libraries we analyzed. Dilutions to obtain the calibration curve were 100 pg/µl, 10 pg/µl, 1 pg/µl, and 100 fg/µl. Templates were used at 5 µl per PCR mixture.

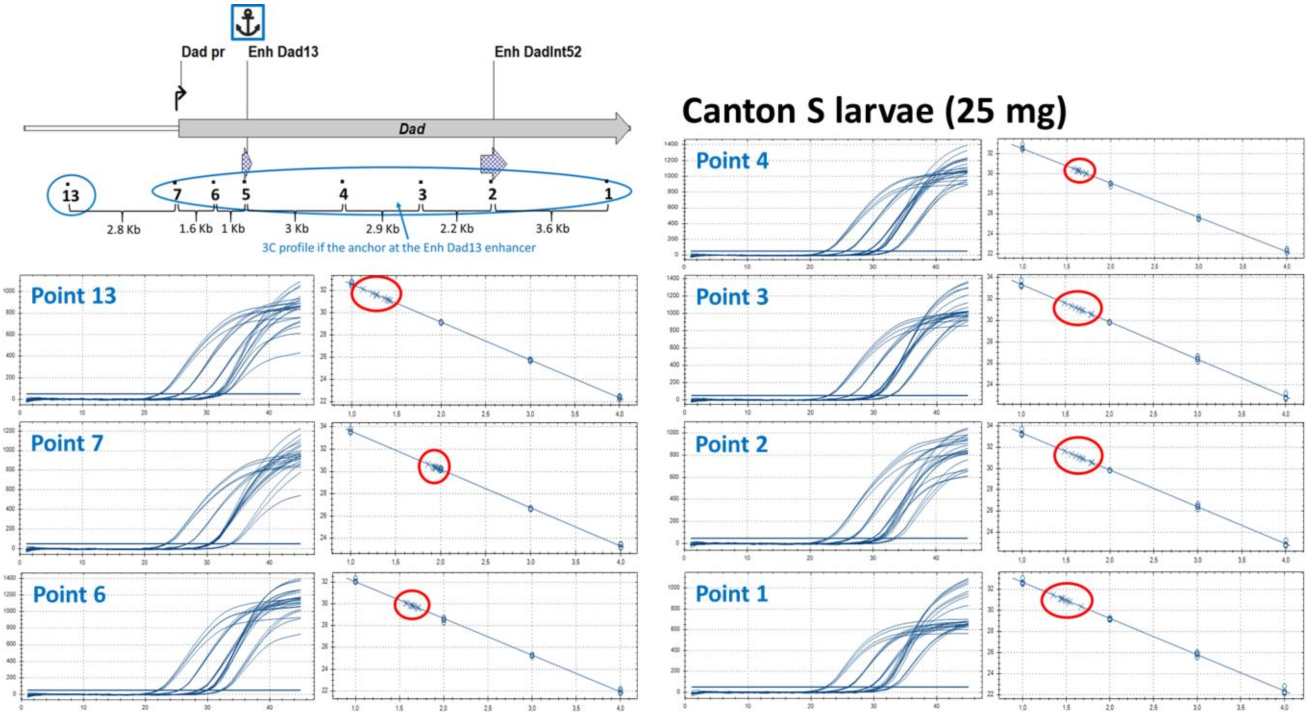

Figure S11. Distribution of experimental 3C samples over the calibration curve in an experiment with wild-type Canton S larvae.

A library was prepared from about 25 mg of larval cell material. The model wild-type locus *Dad* is presented. Positions of 3C samples on the logarithmic calibration curve are shown with red circles. Designations of the X and Y axes are as in Fig. S5. Other designations are as in Fig. S6. Six independent 3C libraries we analyzed. Dilutions to obtain the calibration curve were 100 pg/µl, 10 pg/µl, 1 pg/µl, and 100 fg/µl. Templates were used at 5 µl per PCR mixture.

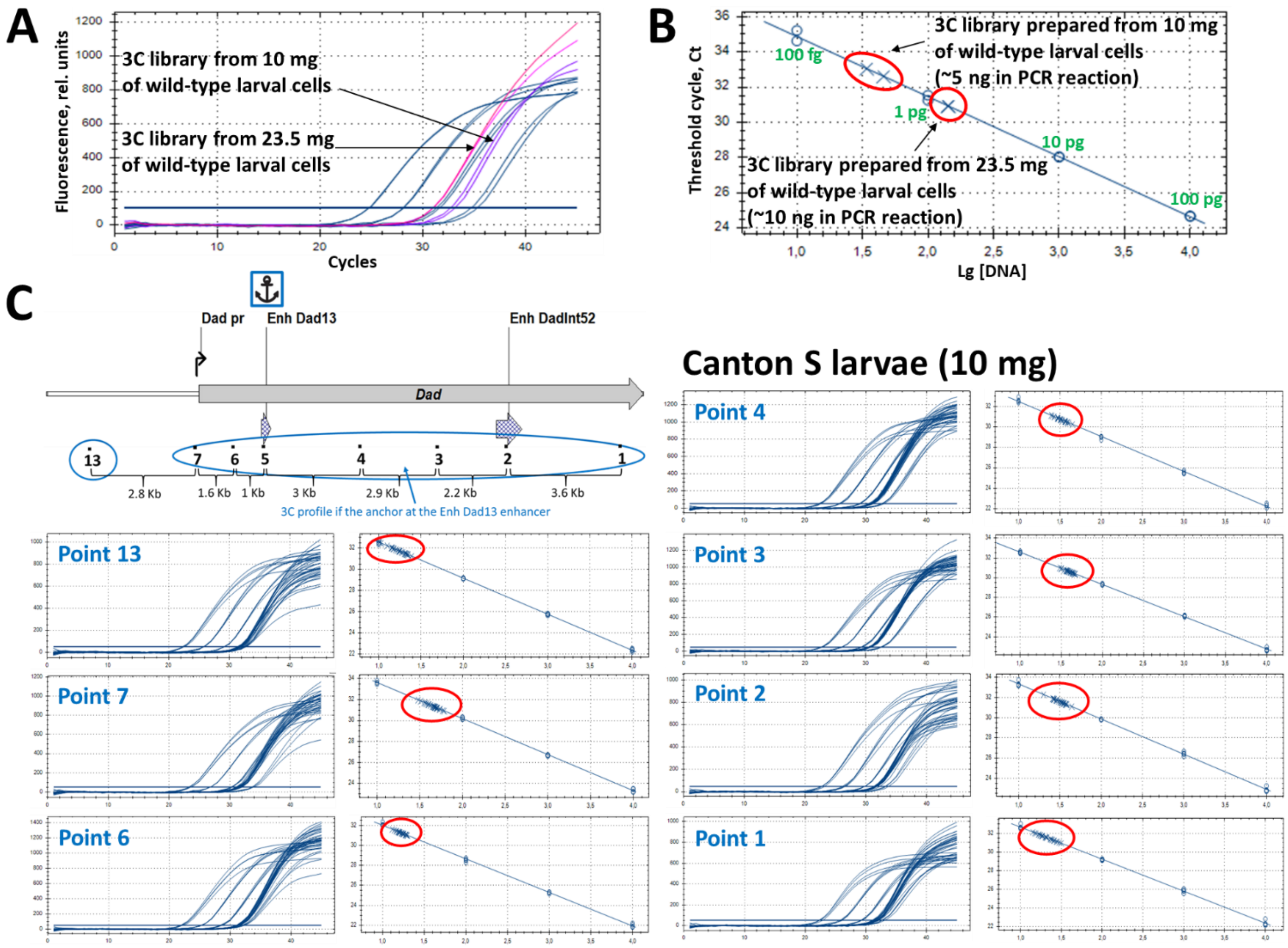

Figure S12. Comparison of the 3C libraries prepared from different amounts of wild-type larval cell material.

(A) The qPCR amplification curves for 3C libraries prepared from 10 (purple lines) and 23.5 mg (pink lines) of larval cell material are shown against a calibration curve made from 10X dilutions of a random ligation library. Amplification was carried out with primers #5-7 (Table S4). The 3C libraries were prepared as in Fig. S6 and were resuspended in 180 µl. The final concentration of 3C samples were 1.017 and 1.99 ng/µl for 10 and 25 mg of larval cell material, respectively. The 3C templates were added at 5 µl per PCR mixture, giving the DNA amounts of 5.085 and 9.96 ng per PCR mixture, respectively. Designations of the X and Y axes are the same as in Fig. S5. Six independent 3C libraries were analyzed. (B) The same as in (A), but the positions of the 3C libraries made from 10 and 25 mg of larval cell material on a logarithmic calibration curve are shown with red circles. (C) Distribution of experimental 3C samples over the calibration curve in an experiment with wild-type Canton S larvae. Model wild-type locus *Dad* is shown. A library was prepared from 10 mg of larval cell material. Positions of 3C samples on a logarithmic calibration curve are shown with red circles. Designations of the X and Y axes are as in Fig. S5. Other designations are as in Fig. S6. Six independent 3C libraries we analyzed. Dilutions to obtain the calibration curve were 100 pg/µl, 10 pg/µl, 1 pg/µl, and 100 fg/µl. Templates were used at 5 µl per PCR mixture.

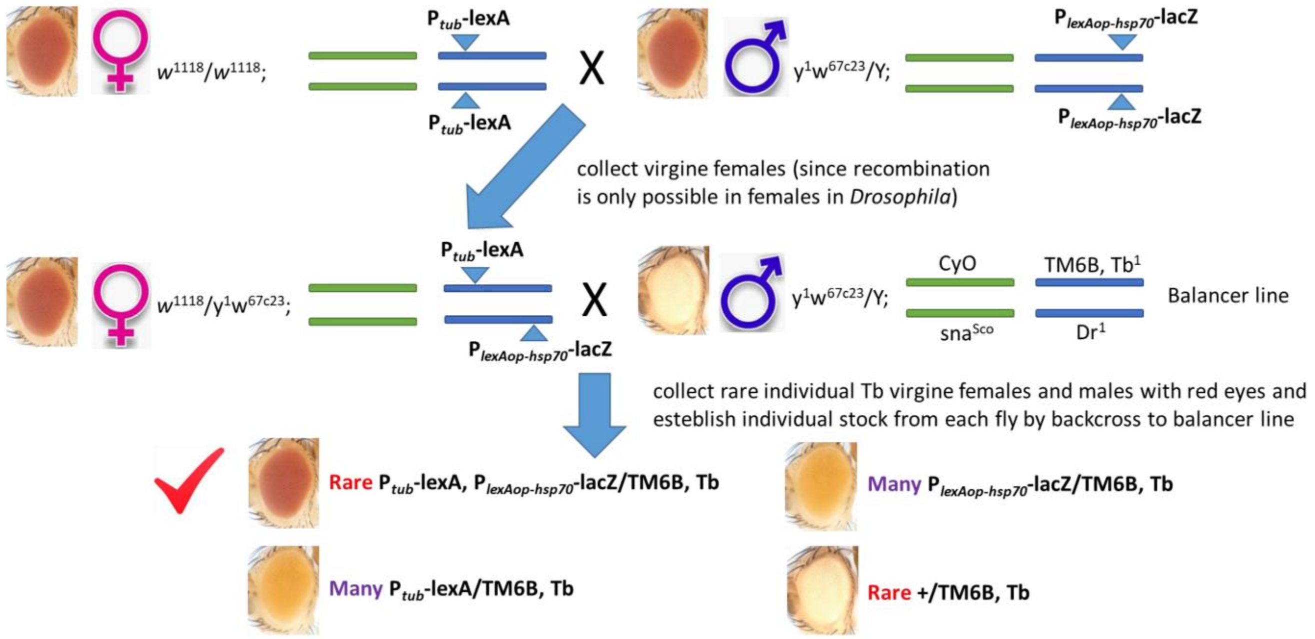

Figure S13. Recombination scheme for obtaining the P$_{tub}$-lexA, P$_{lexAop-hsp70}$-lacZ control line.

Females of a fly line carrying the P-element transgene that contained a coding sequence for the DNA-binding domain of the LexA protein under the control of the α-tubulin gene promoter and the marker gene *mini-white* in homozygote were crossed to males carrying the P-element transgene that contained the reporter *lacZ* gene under the control of the lexAop-hsp70 promoter and the marker gene *mini-white* in homozygote. Virgin females were collected from the offspring of this cross (crossing over occurs in the germline of these females) and were crossed to males of a balancer line, which contained the second and third chromosomes marked with dominant mutations. Virgin females and males with a bright eye color, which indicated the presence of two P-elements, were selected from the offspring of the second cross. Recombinant flies with a bright eye color are rarer in the offspring than flies that carry only one transgene and have yellow eyes. Recombinant flies that carry a wild-type chromosome recovered after recombination have white eyes and are also rare in the offspring. Rare crossover offspring with a bright eye color were individually backcrossed to balancer line flies. Third-cross offspring that had the same eye color as the parents (bright eye color) were crossed to each other, and individual fly lines were established from the offspring of the fourth cross. Forty lines were obtained and were checked for the presence of both transgenes by PCR. A representative example of PCR checking is shown in Fig. 3E.

5 Protocol of the 3C experiment with Drosophila larvae

The initial stages of the protocol, including the isolation of larvae from food, their separation by age, homogenization, fixation, inactivation of FA, and preparation of a larval cell material, are carried out as described in detail in sections 2.1–2.4 of the main part of this article.

To prepare a single 3C library, 25 mg of cell material obtained from wild-type 3rd instar larvae, 50 mg of cell material obtained from mutant larvae (contain less DNA than wild-type larvae), or 10 mg of S2 cells (polyploid cells, contain a lot of DNA) was taken. To homogenize 100 larvae, a 7-ml Dounce homogenizer was used (the yield was ~50 mg of cell material); to homogenize 200 larvae, a 15-ml homogenizer was used (the yield was ~100 mg). Several replicates are generally recommended to perform. This protocol suggests the use of one fixing agent (formaldehyde) and DpnII. A cell material can be frozen at −80 °C after preparation; however, it is better to proceed to the lysis stage to avoid cell material over-fixation, as experiments have shown (Bylino et al., 2021). Protocol steps 1 to 25 resemble the protocol for S2 cells published in (Bylino et al., 2021). Differences from the protocol for S2 cells that are important in the case of larvae are in bold.

Approximate yield of 3C library DNA in the procedure:

A 10-mg quantity of Canton S wild-type larvae yields 156±17 ng of 3C library DNA (ligation) when 1/10 of the initial amount of cell material is taken to obtain control #1 (chromatin integrity control) and 1/10, control #2 (restriction digestion control). The data are represented as M±SD, n = 6. This DNA concentration allowed us to study 7 experimental points in 4 technical replicates. The results fell within the range of a calibration curve made from a random ligation library between the 1 pg/µl and 100 fg/µl dilutions (5 µl of template were taken into the reaction).

A 23.5-mg quantity Canton S wild-type larvae yields 376±57 ng of 3C library DNA (ligation) when 1/20 of the initial amount of cell material is taken to obtain control #1 and 10.5 mg, control #2 (discussed in the text of the protocol). The data are represented as M±SD, n = 6. This DNA concentration allowed us to study 7 experimental points in 4 technical replicates. The results fell within the range of a calibration curve made from a random ligation library between the 1 pg/µl and 100 fg/µl dilutions.

A 10-mg quantity of S2 cells (Sg4 clone) yields 2168±219 ng of 3C library DNA (ligation) when 1/10 of the initial amount of cells is taken to obtain control #1 and 1/10, control #2. The data are represented as M±SD, n = 6. This DNA concentration allowed us to study 7 experimental points in 4 technical replicates. The results fell within the range of a calibration curve made from a random ligation library between the 10 pg/µl and 1 pg/µl dilutions.

With mutant (transgenic) larvae, the yield varies significantly from line to line and apparently reflects the degree of polytenization depending on the genetic background:

A 50-mg quantity of mutant larvae yields 312±236 ng of 3C library DNA (ligation) when 1/20 initial amount of cell material is taken to obtain control #1 and 10.5 mg, control #2. The data are represented as M±SD, n = 12. This DNA concentration allowed us to study 7 experimental points in 2-4 technical replicates. The results generally fell within the range of a calibration curve made from a random ligation library between the 1 pg/µl and 100 fg/µl dilutions and sometimes between the 10 pg/µl and 1 pg/µl dilutions.

I. *Cell lysis*

1. Resuspend the cell material pellet of each replicate in 250 µl of ice-cold Lysis Buffer (50 mM Tris–HCl, pH 8.0, 10 mM NaCl, 0.5% NP-40, 1% Triton X-100, protease inhibitors), incubate in ice for 10-15 min.
2. Centrifuge at 5 k*g* at 4°C for 1 min, carefully remove the supernatant completely.
3. Wash the pellet with 250-500 µl of ice-cold Lysis Buffer once **or twice**. Centrifuge at 5 k*g* at 4°C for 1 min, carefully remove the supernatant completely.

Possible stopping point: At this stage, the nuclei can be left in the washing buffer to stay overnight in ice **in the first or in the second washing** (this does not lead to DNA degradation). If this option is chosen, sediment the nuclei at 7.5 k*g* after overnight incubation in ice and wash once with 250-500 µl of ice-cold Lysis Buffer again.

Possible stopping point: The nuclear pellet could be flash frozen now in liquid nitrogen and stored at −80°C, if required (optional stopping point). However, we recommend proceeding to the next step immediately.

II. *Nucleoplasm release and chromatin treatment with heat*

4. Resuspend the nuclear pellet in 250 µl of 0.1-0.3% SDS in water. Incubate at 65°C for 5 min (alternatively, incubate at 37°C for 10 min–1 h) (shake at **1,100 rpm** to prevent sedimentation of the nuclei).
5. Add 25 µl of 20% Triton-X100 (1.8% final). Incubate min at 37°C for 15 (alternatively incubate at 37°C for 1 h) (shake at **1,100 rpm**).
6. Centrifuge at 5 k*g* at 4°C for 1 min.
7. Wash the nuclei three times with 100–200 µl of ice-cold 1X restriction buffer (RB) for DpnII (50 mM Bis-Tris-HCl, pH 6.0, 100 mM NaCl, 10 mM $MgCl_2$, 1 mM DTT). Centrifuge at no more than **2.5 k*g*** at 4°C for 1 min between washings. Resuspend the pellet by pipetting or vortexing between washings. During the final washing, resuspend the nuclei in 100–200 µl of 1X RB, centrifuge at a low speed, and remove the supernatant so that 50 µl of the supernatant remains in the tube with the nuclei. Gently resuspend the nuclei by pipetting if necessary.

Possible stopping point: At this stage, the nuclei can be left in 1X RB to stay in ice overnight (this does not lead to DNA degradation).

8. Take 1/10 (5 µl) or 1/20 (2.5 µl) of the volume as a Chromatin Integrity Control (Control #1) (we recommend taking 1/10 of the volume when using 10 mg of larval starting cell material and 1/20 when using 25–50 mg of cell material). Add **217.5 µl** of 1X T4 DNA Ligase Buffer (a buffer without ATP is recommended) to the control sample and keep the control in ice or freeze at −20°C. However, we recommend processing the control immediately and proceeding to step 19.

III. *Digestion of DNA in nuclei*

13. Add 2 µl of DpnII (100 U) (NEB). Incubate at 37°C for at least 3 h (overnight incubation is recommended) (shake at **1,200 rpm** to prevent sedimentation of the nuclei).

14. Centrifuge at 7.5 k*g* at 4°C for 1 min.

15. Wash the pellet three times with 100 µl of 1X T4 DNA Ligase Buffer (a buffer without ATP is recommended). Centrifuge at 7.5 k*g* at 4°C for 1 min between washings. Discard the supernatant.

IV. *Ligation of DNA in nuclei*

16. Resuspend the nuclei in 47.5 µl of 1X T4 DNA Ligase Buffer. **Take a suspension volume corresponding to initial 10.575 mg of cell material** as a Digestion Control (Control #2) (we recommend taking exactly this number of cells at this step rather than 1/10 of the volume, even if the starting amount of material is 25 or 50 mg, since an electrophoretically undetectable amount of DpnII-digested DNA was obtained from 1/10 of the sample volume when a 3C library was prepared from 10 mg of starting larval cell material, and 10.575 mg gives reproducible easily detectable result). Adjust the volume of the control sample to **220 µl** using 1X T4 DNA Ligase Buffer and keep the control in ice or freeze at −20 °C. However, we recommend processing the control immediately and proceeding to step 19.

17. Adjust the volume of the ligation reaction mixture to **40 µL**. Add 1 µl (10 Weiss U) of highly concentrated T4 DNA ligase (Sibenzyme) (0.25 Weiss U/µl final concentration), 0.4 µl of 100 mM ATP (1 mM final), and 0.2 µl of 20% Triton X-100 (0.1% final) (prevents adhesion of nuclei on the walls of the tubes during shaking). Incubate at 16°C or at RT overnight (shake at 1,400 rpm to prevent sedimentation of the nuclei). The T4 DNA ligase concentration at this stage should be between 0.025 and 0.25 Weiss U/µl to maintain efficient ligation).

V. *Reversion of cross-links and isolation of a 3C library*

18. Add 179 µl of 1X T4 DNA Ligase Buffer to the ligation sample.

19. Process the controls collected previously from this step on. Add 2.5 µl of Proteinase K (200 µg/ml final), 12.5 µl of 20% SDS (1% final), and 15 µl of 0.5M EDTA (30 mM final) to the control samples #1, 2 and to the ligation sample (a premix of SDS, EDTA, and Proteinase K in 1X T4 DNA Ligase Buffer can be done). Incubate at 56°C overnight, cool to RT.

20. Add an equal volume of phenol/chloroform (1:1, pH 8.0), mix intensely by vortexing. Centrifuge at a maximum speed at RT for 5 min. Collect the aqueous phase and add an equal volume of chloroform. Centrifuge at a maximum speed at RT for 3 min. Collect the aqueous phase (approximately 250 µl). Avoid taking the interphase at this step, as it can lead to DNA degradation after precipitation and dissolution of the samples.

21. Add 27.5 µl of 3M NaOAc and 5 µl of glycogen (100 µg/ml final). Add 750 µl of 96% EtOH, incubate at −80°C for 0.5 h. Centrifuge at a maximum speed at 4°C for 20 min. Dissolve the pellet in 20 µl (**20 µl** for control samples) of 10 mM Tris-HCl pH 8.0.

VI. *Treatment of the 3C library with RNase*

22. Add 1 µl (10 U) of RNase I (Thermo). Alternatively, 0.5–1 µl (0.5–1 µg) of rRNase A or 0.5 µl (5 µg) of bovine RNase A (Thermo) could be used. Incubate at RT for 30 min.

VII. *Purification of the 3C library on magnetic beads and DNA analysis*

23. Purify DNA using magnetic beads (Beckman Coulter AMPure XP beads) according to the manufacturer's protocol with the following modifications: add a 1.5X volume of a bead suspension to the 3C library sample and Chromatin Integrity Control and a 2X volume to the Chromatin Digestion Control, wash the bead pellet three times with 1 ml of 75% EtOH with vortexing, elute with 10 mM Tris-HCl, pH 8.0, using **30 µl** for the 3C library sample and **20 µl** for the control samples, at RT. If magnetic beads are not available do not use EDTA in DNA extraction buffer during reversion of cross-links because EDTA will inhibit PCR after DNA precipitation with EtOH (use SDS only).

24. Measure the DNA concentration using Qubit (dsDNA HS kit) according to manufacturer's recommendations and 1 µl of the samples. Incubate for at least 15 min after adding the dye to the control and experimental samples.

25. Analyze the control samples and the 3C library by electrophoresis now. Load **25 ng** of DNA for Control #1, **50 ng** for Control #2 and **75 ng** for ligation per lane. For these amounts of DNA, use an increased exposure when shooting the gel after electrophoresis. However, we recommend using a DNA ladder in half the amount that is usually used for routine quality control of 3C libraries prepared from 10 mg of S2 cells to prevent overexposure of the DNA ladder lane during shooting (use 0.45 µl of GeneRuler DNA Ladder Mix DNA marker (Thermo #SM0331, conc. 0.5 µg/µL) mixed with 0.4 µl of Purple Gel loading dye 6X (NEB #B7024S) or TriTrack DNA loading dye (Thermo #R1161) and 14.15 µl of 10 mM Tris-HCl, pH 8.0; total volume 15 µl).

The Chromatin Integrity Control lane should contain only high-MW DNA (more than 10-12 kb) and ideally move as a single band without significantly smearing downward. Chromatin Digestion Control may look slightly different for different strains of flies, but should generally correspond to the following description: (i) should be shorter than 3 kb (wild type, see Fig. 2B), shorter than 2 kb

(mutants, Fig. 3B), or even shorter than 1 kb (mutants, see Fig. 3F) with an intensity peak at 1 kb, 0.5 kb, or a smaller size, respectively. The peak intensity of the ligated DNA should ideally be close to 10 kb (in general, the better the ligation is, the more fragments are closer to 10 kb), but depends on the efficiency of chromatin digestion. The peak is observed around 8–10 (wild type, see Fig. 2B), 3.5–5-kb (mutants, Fig. 3B), or 2–4 kp (mutants, see Fig. 3F).

VIII. *Preparation of a random ligation BAC library for constructing a calibration curve*

In the next two sections, we describe in a concise form the steps of growing, isolating, purifying, restricting, and ligating the BAC to generate a random ligation library. For a more detailed description of the experiments, see section 2 (subsections 2.1–2.4) of the Supplement.

26. Electroporate the BAC into the *E. coli* strain EPI300; plate cells on LB agar with 25 μg/ml chloramphenicol (Cm) and grow at 37 °C overnight.

27. Inoculate individual colonies (or material taken from the lawn) into a liquid LB medium and grow in the presence of Cm (25 μg/ml) to a density of 1 OD. Add a BAC replication inducer (arabinose at up to 20 mM) and grow the culture for no more than 2 hours to prevent BAC instability. Alternatively, grow the culture overnight as for an ordinary plasmid in the presence of Cm (25 μg/ml) (in this case, the copy number will be lower and a larger volume of culture will be needed).

28. Isolate the BAC as a common plasmid from small culture volumes using a GeneJET plasmid miniprep kit (Thermo #K0503) or, for preparative isolation, use a GeneJET plasmid midiprep kit (Thermo #K0481) or Plasmid midiprep 2.0 (Evrogen #BC124).

29. Extract the isolated BAC with Ph/Chl and then with Chl alone; precipitate with EtOH in the presence of 0.3M NaOAc, pH5.2 (w/o 100 μg of glycogen); and dissolve in 10 mM Tris-HCl, pH 8.0. Then purify the BAC with AMPure XP beads according to the manufacturer's instructions with modification (use 1 ml of 75% EtOH for washing, wash three times). Elute with 10 mM Tris-HCl, pH 8.0 at RT for 10 min.

30. Measure the DNA concentration using a Qubit HS DNA kit according to the manufacturer's recommendations with modification (the kit measures in a range of 0.2–100 ng/μl according to the manufacturer. In practice, a linear correspondence to 260/280 spectrophotometer readings is observed in a range of 10 ng/μl–100 ng/μl–1 μg/μl. Outside this range, either readings are not measurable or the DNA concentration relates to the readings nonlinearly). Incubate for at least 15 minutes after adding the dye and do not leave to stay until the next day.

31. For restriction digestion, take 25 μg of total DNA (BAC + plasmid containing the required transgenic sequences). Mix in equimolar proportion and digest with 100 U of Sau3AI (BspI), which has the same GATC cleavage site as DpnII, but is insensitive to adenine methylation in contrast to DpnII. Perform digestion in a volume of 100 μl in triplicate. Incubate overnight. Load a gel with 1 μl of the restriction mixture.

32. Purify DNA from the restriction mixture using 3 volumes of AMPure XP beads according to the manufacturer's instructions with modification (wash 3 times with 75% EtOH). Elute DNA with 50 µL of 10 mM Tris-HCl, pH 8.0 at RT for 10 min.

33. Ligate purified DNA in a volume of 50 µl in the presence of 0.4 U/µl T4 DNA ligase (Sibenzyme) and 1 mM ATP at 16 ° C in triplicate. Incubate O/N (the success of ligation after O/N incubation is not affected by the addition of fresh ATP to 1 mM or a new portion of DNA ligase).

34. Pool the three DNA ligation mixtures and purify DNA using 3 volumes of AMPure XP beads. Elute DNA with 150 µl of 10 mM Tris-HCl twice, at RT for 10 min and then at 50 °C for 5 min. Pool the eluates. Use 1 µl of ligated DNA for electrophoresis.

IX. *Preparation of the calibration curve based on a random ligation library*

In the next two sections, we describe in a concise form the steps of 3C library analysis, starting from preparation of a calibration curve. Then we describe how to preliminarily test the 3C samples against the calibration curve. For a more detailed description of the experiments, see section 3 (subsections 3.3, 3.4) of the Supplement.

Predesign TaqMan probes and anchor and test primers so that they target the DNA ends intended for ligation, are as far apart as possible at a distance of no more than 100 bp from the DpnII sites, and the anchor primer and the probe hybridize to different DNA strands [Splinter et al., 2006]. The anchor primer and all test primers should be directed in one direction. The probes are labeled at the 5' end with the FAM dye, and one of the T nucleotides in the interval from the 10th to the 16th base from the 5' end is labeled with the BHQ1 quencher. Optimal TaqMan PCR conditions for detecting the 3C products are as follows: 0.3 µM each primer, 0.3 µM TaqMan probe, 1 U of Hot Start Taq DNA pol (Sibenzyme) or Dream Taq Hot Start (Thermo), 0.2 mM dNTP, 2 mM $MgCl_2$, total reaction volume 10 µl. Perform PCR according to the following program: initial denaturation at 94 °C for 1 min and 45 two-step cycles of 94 °C for 10 sec and 60 °C for 1 min. If it is necessary to make a calibration curve from PCR product(s) (for the purpose of normalization to the control locus or in the case where the BAC is unavailable), the PCR product(s) is prepared as described in section XII of the protocol.

35. To prepare the calibration curve, make a series of 10-fold dilutions from the purified random ligation library (RLL). As a first dilution, take a 10 ng/µL RLL and add 5 µl into the reaction mixture (reaction volume 10 µl); i.e., use 50 ng of RLL per reaction. Linear DNA amplification is observed to a dilution containing 100 fg/µL RLL, i.e., to 500 fg of RLL per reaction. To determine the relative ligation frequency (RLS), assign values of 1, 10, 100, etc. to the calibration dilutions. Assess quality of the prepared calibration curve experimentally for 3-4 pairs of primers with maximum differences in the lengths and GC compositions of the amplicons. Evaluate the efficiency of amplification at the given calibration dilutions (ideally R = ~ 0.999). For routine measurement of RLS in experimental 3C samples, use a calibration range of 1 ng/µl−100 pg/µl−10 pg/µl−1 pg/µl 10X dilutions in 4

technical replicates each. A 100-fg point can be used to increase the depth of the calibration curve at low RLS values but in 8 technical replicates.

X. *Preliminary testing of dilutions of 3C samples against the calibration curve*

To preliminarily assess the content of ligation products in experimental 3C samples, set up an experiment with dilutions of 3C samples. Select the 3C sample dilutions so that there is enough DNA to measure all of the interaction points of interest and, at the same time, the dilutions fall into the calibration range. Introduce an additional calibration point of 10 fg/μL if there is a lack of material. Set the 10 fg/μL point in 8, rather than 4, technical replicates.

36. (*an example for S2 cells*) Adjust the volumes of 3C samples prepared from 10 mg of S2 cells to 83 μl (enough to measure the experimental points). The concentration range of the adjusted samples is from 62.3 to 85.1 ng/μl. Equalize the DNA concentration in different 3C libraries using Qubit readings. Use the sample with the lowest concentration and adjust all other samples to its concentration. Add 1, 2.5, or 5 μl of an undiluted sample with a concentration of 62.3 ng/μl and its 10- and 100-fold dilutions (6.23 and 0.623 ng/μl, respectively; take 1 μl of the diluted samples) to a PCR mixture. Determine the concentration range that fits the calibration curve prepared. Efficient amplification should be observed at a given dilution of the 3C samples, and their volume should be sufficient to measure all experimental points. For example, the concentration range from 62.3 ng/μL to 6.23 ng/μL in the reaction is expected to fit into the calibration curve 100 pg/μl−10 pg/μl−1 pg/μl–100 fg/μl between the 10-pg/μl and 1-pg/μl dilutions and ensures efficient detection of RLS.

(*an example for larvae*) Adjust the volume of 3C samples prepared from wild-type third instar larvae to 180 μl (enough to measure the experimental points). Prior to the experiment, equalized the concentrations of the 3C libraries prepared from larvae of different fly strains according to Qubit readings. Use the sample with the lowest concentration and adjust all other samples to its concentration. Libraries prepared from wild-type larvae have much lower DNA concentrations than the ones prepared from S2 cells, even with a higher dilution. Mutant larvae yield 2–3 times less DNA of the 3C libraries than wild-type larvae do. Therefore, use 50 mg of starting larval cell material to prepare 3C libraries from mutants. Supposing the concentrations of 3C libraries prepared from 10 and 23.5 mg of larval cell material are 1.017 and 1.99 ng/μl, respectively. Take 5 μl of a library for a PCR mixture (5.085 and 9.96 ng of DNA per reaction, respectively). Due to the smaller DNA amount from larvae than from S2 cells, use a calibration curve from 100 pg/μl−10 pg/μl−1 pg/μl−100 fg 10X dilutions. The indicated DNA amounts are expected to fit into the calibration curve between the 10- and 1-pg and between the 1-pg and 100 fg-dilutions of the calibration curve, respectively.

XI. *Preparation of the PCR master mix and the general arrangement of a qPCR experiment with 3C samples*

Once the required dilutions of the 3C samples are found and the range of the calibration curve is determined, proceed to the main qPCR experiment. A scheme for preparing the qPCR mixes for the calibration curve and 3C samples to study 7 experimental points is described in detail in section 4

(subsection 4.1) of the Supplement. Briefly, use 4–5 dilutions of the calibration curve (in the basic version, 1 ng/μl, 100 pg/μl, 10 pg/μl, 1 pg/μl, and 100 fg/μl). The total volume of a PCR mixture is 10 μl; take 5 μl of the template for one PCR sample. First, mix the pre-primer mix (the probe and the anchor primer), distribute it according to the number of points, and add the test primer for each point, getting the primer mix according to the number of points under study. Then mix the master (enzyme) mix (all components except templates) and add the master mix to the primer mix for each point. Distribute the combined mix among the template tubes. Each tube with a template contains 21 μL of the template. Add 21 μL of the combined mix to the templates and dispense 4 technical replicates for each point.

XII. *Normalization of ligation frequencies*

To allow for variations in cross-linking, restriction, and ligation efficiencies; the quality of 3C libraries; and the amount of DNA taken for analysis and because different fly lines are studied to obtain the ligation frequencies, the ligation frequencies measured for the experimental samples are normalized to the ligation frequency of the point that is in the intergenic spacer between the gene of interest (GOI) and an adjacent gene (point #13, see Fig. S3A, Fig. 4A,C). This point is covered with the BAC. Since this point is far from the GOI (~2.5 kb), its ligation with the enhancer situated inside the GOI and with other parts of the GOI is probably meaningless and can be perceived as a background interaction. The cross-linking frequency in this point can be arbitrarily taken as a cross-linking frequency value of 1. At the same time, despite the fact that there is no reason to believe that the spacer region, together with the GOI itself, can be in different conformation in the control and experimental fly lines, this possibility cannot be completely excluded. In this regard, the ligation frequencies measured in the control and experimental fly lines are additionally normalized to the ligation frequency between adjacent DpnII fragments in the *RpII* gene (RNAP II subunit A) (Comet et al., 2011). The experimental design of primers and probes for estimating the ligation frequency at the *RpII* locus in presented in the Fig. S6E. Thus, the ratios between the experimental and control lines are normalized twice for each point. The values are averaged between the two normalizations. The normalization does not affect the nature of the profile, but can slightly change the absolute values [Gavrilov, Razin, 2008]. To prepare the calibration curve for normalization to *RpII* do as follows:

37. First, construct a calibration curve from 10-fold dilutions of the PCR product obtained with the primers RpII_1 and Rp_3 (Fig. S6E). Perform preparative PCR in 100 μL in several tubes as follows: initial denaturation at 94 °C for 1 min, 30 cycles (94 °C for 10 sec, 60 °C for 20 sec, and 72 °C for 15 sec), and final extension at 72 °C for 1 min. A PCR mixture contained 0.2 μM each primer, 2 mM $MgCl_2$, 1 U of Hot Start Taq (SibEnzyme, #E352) or Dream Taq Hot Start (Thermo) DNA polymerase in a volume of 10 μl.

38. Excise and purify the PCR products for calibration from gel (use combs with wide wells, a Monarch Gel extraction Kit, or a NEB or GeneJET Gel Extraction Kit, Thermo; wash the column with an isopropanol-containing buffer during isolation). Then additionally purify the PCR products

two times from agarose traces using a 3X volume of AMPure XP beads according to the manufacturer's instruction with modification (beads were washed with 1 ml of 75% EtOH 3 times with vortexing) and dry in the open vials at RT for 2 min. Elute DNA with 10 mM Tris-HCl, pH 8.0 at RT for 10 min.

39. Determine the amount of the ligated product at the *RpII* locus using the calibration curve. First, create a calibration curve from 10-fold dilutions of the purified PCR product. Then, determine the amount of the common fragment (reference sequence) in the 3C samples with the primers RpII_1 and Rp_2, probe RpII_0 (Fig. S6E), and equation in Fig. S6F. Determine the amount of the 3C ligation product with the primers RpII_1 and RpII_3 and the same probe. The amount of the product obtained with the primers RpII_1 and RpII_3 is usually 6-10% of the amount of the reference fragment. At the same time, when the 3C signal is measured within the same tissue or cell line, the amount of the 3C products can be normalized relative to an amplicon that does not overlap the DpnII site (Bieli et al., 2015). In this case, the total amount of DNA is determined with the primers RpII_1 and RpII_2.

XIII. Calculation and statistical analysis of the 3C experimental results

The issues of calculating the results of a 3C experiment are discussed in detail in section 4 (subsections 4.2, 4.3) of the Supplement. Briefly, calculate the relative ligation frequencies based on the calibration curve for the main experiment and normalization. Perform calculations using the exponential function equation or the trend function built into Excel. Calculate the arithmetic mean. Use the standard deviation (SD) for technical replicates and the standard error of the mean (SEM) for biological replicates. Use tailed Student's *t*-test to calculate the reliability of the differences revealed. Calculate the P-value based on the values of the *t*-test.

6 References

Belaghzal, H., Dekker, J., and Gibcus, J. H. (2017). Hi-C 2.0: An optimized Hi-C procedure for high-resolution genome-wide mapping of chromosome conformation. *Methods* 123, 56–65. doi: 10.1016/j.ymeth.2017.04.004.

Better, M. D. (2004). Methods and cells for expression of recombinant protein products under the transcriptional control of an inducible promoter. Available at: https://patents.google.com/patent/US6803210B2/en [Accessed November 30, 2020].

Bieli, D., Kanca, O., Requena, D., Hamaratoglu, F., Gohl, D., Schedl, P., et al. (2015). Establishment of a Developmental Compartment Requires Interactions between Three Synergistic Cis-regulatory Modules. *PLOS Genetics* 11, e1005376. doi: 10.1371/journal.pgen.1005376.

Bochner, B. R., Huang, H. C., Schieven, G. L., and Ames, B. N. (1980). Positive selection for loss of tetracycline resistance. *Journal of Bacteriology* 143, 926–933.

Bujard, H., and Gossen, M. (1997). Tetracycline-inducible transcriptional activator and tetracycline-regulated transcription units. Available at: https://patents.google.com/patent/US5654168A/en [Accessed November 27, 2020].

Bylino, O. V., Ibragimov, A. N., Pravednikova, A. E., and Shidlovskii, Y. V. (2021). Investigation of the Basic Steps in the Chromosome Conformation Capture Procedure. *Front Genet* 12, 733937. doi: 10.3389/fgene.2021.733937.

Canela, A., Maman, Y., Jung, S., Wong, N., Callen, E., Day, A., et al. (2017). Genome Organization Drives Chromosome Fragility. *Cell* 170, 507-521.e18. doi: 10.1016/j.cell.2017.06.034.

Chathoth, K. T., and Zabet, N. R. (2019). Chromatin architecture reorganization during neuronal cell differentiation in Drosophila genome. *Genome Res* 29, 613–625. doi: 10.1101/gr.246710.118.

Comet, I., Schuettengruber, B., Sexton, T., and Cavalli, G. (2011). A chromatin insulator driving three-dimensional Polycomb response element (PRE) contacts and Polycomb association with the chromatin fiber. *PNAS* 108, 2294–2299. doi: 10.1073/pnas.1002059108.

Cubeñas-Potts, C., Rowley, M. J., Lyu, X., Li, G., Lei, E. P., and Corces, V. G. (2017). Different enhancer classes in Drosophila bind distinct architectural proteins and mediate unique chromatin interactions and 3D architecture. *Nucleic Acids Research* 45, 1714–1730. doi: 10.1093/nar/gkw1114.

de Cristóbal, R. E., Vincent, P. A., and Salomón, R. A. (2006). Multidrug resistance pump AcrAB-TolC is required for high-level, Tet(A)-mediated tetracycline resistance in Escherichia coli. *J Antimicrob Chemother* 58, 31–36. doi: 10.1093/jac/dkl172.

Deshmukh, S., Ponnaluri, V. C., Dai, N., Pradhan, S., and Deobagkar, D. (2018). Levels of DNA cytosine methylation in the Drosophila genome. *PeerJ* 6. doi: 10.7717/peerj.5119.

Eagen, K. P., Aiden, E. L., and Kornberg, R. D. (2017). Polycomb-mediated chromatin loops revealed by a subkilobase-resolution chromatin interaction map. *PNAS* 114, 8764–8769. doi: 10.1073/pnas.1701291114.

Eagen, K. P., Hartl, T. A., and Kornberg, R. D. (2015). Stable Chromosome Condensation Revealed by Chromosome Conformation Capture. *Cell* 163, 934–946. doi: 10.1016/j.cell.2015.10.026.

El-Sharnouby, S., Fischer, B., Magbanua, J. P., Umans, B., Flower, R., Choo, S. W., et al. (2017). Regions of very low H3K27me3 partition the Drosophila genome into topological domains. *PLOS ONE* 12, e0172725. doi: 10.1371/journal.pone.0172725.

Gavrilov, A. A., and Razin, S. V. (2008). Spatial configuration of the chicken α-globin gene domain: immature and active chromatin hubs. *Nucleic Acids Res* 36, 4629–4640. doi: 10.1093/nar/gkn429.

Hug, C. B., Grimaldi, A. G., Kruse, K., and Vaquerizas, J. M. (2017). Chromatin Architecture Emerges during Zygotic Genome Activation Independent of Transcription. *Cell* 169, 216-228.e19. doi: 10.1016/j.cell.2017.03.024.

Li, L., Lyu, X., Hou, C., Takenaka, N., Nguyen, H. Q., Ong, C.-T., et al. (2015). Widespread rearrangement of 3D chromatin organization underlies Polycomb-mediated stress-induced silencing. *Mol Cell* 58, 216–231. doi: 10.1016/j.molcel.2015.02.023.

Livak, K. J., and Schmittgen, T. D. (2001). Analysis of Relative Gene Expression Data Using Real-Time Quantitative PCR and the 2−ΔΔCT Method. *Methods* 25, 402–408. doi: 10.1006/meth.2001.1262.

Louwers, M., Splinter, E., van Driel, R., de Laat, W., and Stam, M. (2009). Studying physical chromatin interactions in plants using Chromosome Conformation Capture (3C). *Nature Protocols* 4, 1216–1229. doi: 10.1038/nprot.2009.113.

Manikandan, S. (2011). Measures of central tendency: The mean. *J Pharmacol Pharmacother* 2, 140–142. doi: 10.4103/0976-500X.81920.

Martinez, M. N., and Bartholomew, M. J. (2017). What Does It “Mean”? A Review of Interpreting and Calculating Different Types of Means and Standard Deviations. *Pharmaceutics* 9. doi: 10.3390/pharmaceutics9020014.

Miele, A., and Dekker, J. (2009). “Mapping Cis- and Trans- Chromatin Interaction Networks Using Chromosome Conformation Capture (3C),” in *The Nucleus: Volume 2: Chromatin, Transcription, Envelope, Proteins, Dynamics, and Imaging* Methods in Molecular Biology., ed. R. Hancock (Totowa, NJ: Humana Press), 105–121. doi: 10.1007/978-1-60327-461-6_7.

Miele, A., Gheldof, N., Tabuchi, T. M., Dostie, J., and Dekker, J. (2006). Mapping chromatin interactions by chromosome conformation capture. *Curr Protoc Mol Biol* Chapter 21, Unit 21.11. doi: 10.1002/0471142727.mb2111s74.

Naumova, N., Smith, E. M., Zhan, Y., and Dekker, J. (2012). Analysis of long-range chromatin interactions using Chromosome Conformation Capture. *Methods* 58, 192–203. doi: 10.1016/j.ymeth.2012.07.022.

Rao, S. S. P., Huntley, M. H., Durand, N. C., Stamenova, E. K., Bochkov, I. D., Robinson, J. T., et al. (2014). A three-dimensional map of the human genome at kilobase resolution reveals principles of chromatin looping. *Cell* 159, 1665–1680. doi: 10.1016/j.cell.2014.11.021.

Rao, X., Huang, X., Zhou, Z., and Lin, X. (2013). An improvement of the 2ˆ(–delta delta CT) method for quantitative real-time polymerase chain reaction data analysis. *Biostat Bioinforma Biomath* 3, 71–85.

Rowley, M. J., Nichols, M. H., Lyu, X., Ando-Kuri, M., Rivera, I. S. M., Hermetz, K., et al. (2017). Evolutionarily Conserved Principles Predict 3D Chromatin Organization. *Mol. Cell* 67, 837-852.e7. doi: 10.1016/j.molcel.2017.07.022.

Ruiz-Villalba, A., van Pelt-Verkuil, E., Gunst, Q. D., Ruijter, J. M., and van den Hoff, M. J. (2017). Amplification of nonspecific products in quantitative polymerase chain reactions (qPCR). *Biomol Detect Quantif* 14, 7–18. doi: 10.1016/j.bdq.2017.10.001.

Schmittgen, T. D., and Livak, K. J. (2008). Analyzing real-time PCR data by the comparative CT method. *Nat Protoc* 3, 1101–1108. doi: 10.1038/nprot.2008.73.

Sexton, T., Yaffe, E., Kenigsberg, E., Bantignies, F., Leblanc, B., Hoichman, M., et al. (2012). Three-Dimensional Folding and Functional Organization Principles of the Drosophila Genome. *Cell* 148, 458–472. doi: 10.1016/j.cell.2012.01.010.

Shidlovskii, Y. V., Bylino, O. V., Shaposhnikov, A. V., Kachaev, Z. M., Lebedeva, L. A., Kolesnik, V. V., et al. (2021). Subunits of the PBAP Chromatin Remodeler Are Capable of Mediating Enhancer-Driven Transcription in Drosophila. *Int J Mol Sci* 22. doi: 10.3390/ijms22062856.

Smothers, C. D., Sun, F., and Dayton, A. D. (1999). Comparison of arithmetic and geometric means as measures of a central tendency in cattle nematode populations. *Veterinary Parasitology* 81, 211–224. doi: 10.1016/S0304-4017(98)00206-4.

Splinter, E., Heath, H., Kooren, J., Palstra, R.-J., Klous, P., Grosveld, F., et al. (2006). CTCF mediates long-range chromatin looping and local histone modification in the beta-globin locus. *Genes Dev* 20, 2349–2354. doi: 10.1101/gad.399506.

Thummel, C. S., Boulet, A. M., and Lipshitz, H. D. (1988). Vectors for Drosophila P-element-mediated transformation and tissue culture transfection. *Gene* 74, 445–456. doi: 10.1016/0378-1119(88)90177-1.

Ulianov, S. V., Khrameeva, E. E., Gavrilov, A. A., Flyamer, I. M., Kos, P., Mikhaleva, E. A., et al. (2016). Active chromatin and transcription play a key role in chromosome partitioning into topologically associating domains. *Genome Res* 26, 70–84. doi: 10.1101/gr.196006.115.

Ulianov, S. V., Zakharova, V. V., Galitsyna, A. A., Kos, P. I., Polovnikov, K. E., Flyamer, I. M., et al. (2021). Order and stochasticity in the folding of individual Drosophila genomes. *Nature Communications* 12, 41. doi: 10.1038/s41467-020-20292-z.

Venken, K. J. T., Carlson, J. W., Schulze, K. L., Pan, H., He, Y., Spokony, R., et al. (2009). Versatile P[acman] BAC libraries for transgenesis studies in Drosophila melanogaster. *Nature Methods* 6, 431–434. doi: 10.1038/nmeth.1331.
